# Atomistic Simulation‐Driven Design of STM Tips for NiCp_2_ Adsorption and Spin‐State Modulation

**DOI:** 10.1002/smll.202508320

**Published:** 2025-10-22

**Authors:** Nanchen Dongfang, Federico Totti, Marcella Iannuzzi

**Affiliations:** ^1^ Department of Chemistry University of Zurich Zurich 8057 Switzerland; ^2^ Dipartimento di Chimica “Ugo Schiff” & INSTM RU Università degli Studi di Firenze Via della Lastruccia 3 Sesto Fiorentino (FI) 50019 Italy

**Keywords:** copper tip, density functional theory, magnetic exchange coupling, multi‐reference methods, NiCp_2_, spin‐polarized STM, spin‐state modulation

## Abstract

Despite the widespread use of scanning tunneling microscopy (STM) in atomic‐scale investigations, the influence of the tip's atomic structure remains insufficiently characterized. This study addresses the issue by analyzing the electronic and magnetic properties of transition‐metal‐functionalized STM tips using both multireference wavefunction methods and density functional theory. The results demonstrate that strong electron correlations in transition‐metal‐based tips must be accounted for to accurately describe the structural and magnetic parameters involved—an essential requirement for the correct setup of inelastic and scanning tunneling spectroscopy experiments. By considering both minimal tip models and larger, more realistic pyramid structures, the approach balances computational efficiency with experimental relevance. The mechanism of spin‐state reduction in NiCp_2_‐functionalized tips is clarified, revealing the central roles of charge transfer, molecular distortion, and metal–substrate hybridization. Furthermore, selective substitution of the Cu apex atom in Cu(111)‐based tips with 3d transition metals allows controlled modulation of the NiCp_2_ spin state. This provides a practical strategy for designing STM tips with tailored magnetic properties. Overall, the findings establish a robust theoretical framework for interpreting complex molecule–substrate interactions in spintronic systems and support the development of next‐generation spin‐polarized STM tips and molecular spintronic devices.

## Graphical abstract

This work explores the atomistic and spin‐dependent properties of NiCp_2_‐functionalized STM tips using multireference and DFT methods. By modeling small and large Cu‐based tip structures, the study reveals how charge transfer, structural distortion, and apex metal substitution influence spin‐state modulation, offering practical insights for designing tailored spin‐polarized STM probes.

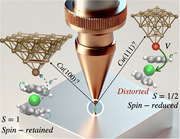

## 1 Introduction

Molecular spintronics, a rapidly advancing field, leverages molecular systems to control and manipulate electron spin, offering exciting prospects for nanoscale devices. Among these, single‐molecule magnets and organometallic compounds have drawn significant attention because of their ability to maintain well‐defined spin states and exhibit distinctive magnetic properties. A key focus within this field is the development of spin‐polarized scanning tunneling microscopy (STM) tips functionalized with magnetic molecules. Such tips enable direct probing and precise manipulation of spin‐dependent interactions at the atomic scale.^[^ ^1^, ^2^, ^3^, ^4^, ^5^ ^]^ Recent advances have proposed various methods for manipulating molecular spin states, achieving unprecedented control over molecular magnetism.^[^ ^6^, ^7^, ^8^, ^9^, ^10^ ^]^ At the same time, understanding the interaction between magnetic molecules and metallic substrates is essential for designing reliable spintronics devices. STM experiments are highly sensitive to the structure of the STM tip, which is strongly correlated with the special tip‐sample interaction mechanism.^[^ ^11^, ^12^, ^13^, ^14^ ^]^ This is even more evident when scanning tunnelling spectroscopy (STS) and Inelastic Electron Tunneling Spectroscopy (IETS) are performed.^[^ ^15^ ^]^ However, detailed atomistic knowledge of the tip, such as its exact geometry, atomic arrangement, and packing structure, remains elusive. This lack of information often results in experimental inconsistencies and hinders reproducibility. Although spin detection offers a potential means to infer details about the tip configuration and exploit this knowledge for other measurements, the lack of comprehensive insights into the tip effect remains a significant challenge for the field.

Copper tips are a common choice in STM thanks to the high electrical conductivity, ease of fabrication, and ability to be sharpened with precision. The conductive and relatively inert nature of Cu substrates make them also good candidates for molecular spintronics applications. The Cu electronic configuration (3*d* ^10^4*s* ^1^), characterized by fully occupied 3d orbitals and a delocalized 4s electron, renders it largely nonmagnetic or weakly paramagnetic. However, the structural details of the tip remain largely unknown, since there is no direct way to reveal them,^[^ ^13^ ^]^ apart from the commonly accepted fact that the apex consists of a single Cu atom. Copper nanostructures pack preferably with two orientations, Cu(100) and Cu(111), which are thermodynamically stable. However, the actual orientation around the STM tip is hardly known, mainly because its precise characterization has not been considered essential for standard STM operations.

The resolution in spin‐polarized STM measurement can be improved by decorating the tip with small magnetic molecules, preferably carrying a few unpaired electrons. In this regard, Nickelocene (NiCp_2_) stands out as a promising candidate for functionalized STM tips due to its intrinsic *S* = 1 spin state, derived from its electronic configuration of (*a*_1*g*_)^2^ (*e*_2*g*_)^4^ (*e*_1*g*_)^2^. The stability of NiCp_2_ arises from its molecular symmetry, defined by two parallel cyclopentadienyl (Cp) rings, which shield it from environmental perturbations. This resilience, coupled with the ability to fine‐tune its magnetic properties, makes NiCp_2_ a strong contender for spintronics applications, since it is expected to enhance both spatial and energy resolution in STM experiments. NiCp_2_‐terminated tips are typically prepared by approaching a bare metallic tip toward an adsorbed molecule under a negative sample voltage.^[^ ^16^ ^]^ For example, NiCp_2_ has been used to explore the spin polarization of magnetic atoms through measurements of inelastic tunneling currents, offering electrical access to its spin states.^[^ ^17^, ^18^ ^]^ When the STM tip is functionalized with a magnetic molecular species, its electronic properties become even more critical, as they directly influence spin‐sensitive measurements. Lack of full control over it introduces an additional degree of uncertainty in the interpretation of electronic and magnetic data. We will discuss how differences in the atomic arrangement between Cu(100) and Cu(111) may affect the functionalized tip, leading to redox processes, charge transfer, structural distortions, and spin interactions.

The manipulation of the magnetic properties of the NiCp_2_ functionalized tip has already been discussed in the literature. Ormaza and Bachllier and co‐workers^[^ ^6^, ^19^ ^]^ investigated a NiCp_2_‐terminated tip model made of a Cu(100) substrate with a Cu adatom, as apex. They observe that, under contact conditions, i.e., when NiCp_2_ is “squeezed” between tip and sample, the spin switching from *S* = 1 to *S* = 1/2 is induced. Competing effects, such as spin excitation (magnetic anisotropy) and Kondo resonance, were also associated to the spin reduction. Zuo et al.^[^ ^20^ ^]^ proposed a Cu(100) blunt‐tip model and obtained a similar spin‐reduction effect. Experimentally, Mohr et al.^[^ ^21^ ^]^ have reported evidence of NiCp_2_ tilting during STM measurements, which associates it with mechanical stress induced when approaching the sample.

The reported on‐going debate on the interdependence between structural and magnetic features underscores the need for accurate atomistic models to predict the tip behavior and allow for the correct interpretation of the measurements. Furthermore, deliberate modifications of tip apexes, such as replacing the Cu atom with TMs, offer opportunities to tailor the electronic and magnetic properties of NiCp_2_, allowing the design of specialized tips with desired functionalities. Addressing spin‐state transition often requires theoretical approaches capable of accurately describing strongly correlated systems and near‐degenerate spin states.^[^ ^22^ ^]^ Density functional theory (DFT) methods^[^ ^23^, ^24^ ^]^ are widely used for systems such as NiCp_2_ and other metallocenes adsorbed on substrates.^[^ ^6^, ^17^, ^18^, ^25^, ^26^ ^]^ Its limitations in describing multimagnetic center systems reside in the correct calculation of the static correlation. Post‐Hartree‐Fock (post‐HF) methods, such as the Complete Active Space Self‐Consistent Field (CASSCF)^[^ ^27^ ^]^ and the N‐electron Valence Perturbation Theory (NEVPT2),^[^ ^28^, ^29^, ^30^ ^]^ provide a robust framework for addressing these challenges by accounting for both static and dynamic electron correlations. On the other hand, the choice of the active space may become especially critical for systems involving transition metal complexes, demanding high computational costs. The combined use of both DFT and post‐HF methods provides a synergistic approach, leveraging the computational efficiency of DFT with the high accuracy of multiconfigurational methods to achieve a comprehensive understanding of complex magnetic systems.

In this work, we shed light on the electronic and magnetic properties of the NiCp_2_‐functionalized STM tip, proposing atomistic models that can be studied at the CASSCF and DFT level. Our primary objective is to elucidate the effects of Cu(100) and Cu(111) substrates on the stability, geometry, and magnetic properties of NiCp_2_. We further explore the functionalization of tips by replacing Cu apex atoms with other TMs, analyzing how these modifications alter the molecular properties and the exchange coupling patterns. By comparing a combination of the CASSCF, NEVPT2, and DFT‐based PBE0‐D3 methods, we evaluate strengths and limitations and provide information on the behavior of the NiCp_2_ functionalized tip systems. This study contributes to the fundamental understanding of molecular spintronics and offers practical guidelines for the advancement of nanoscale magnetic sensing technologies.

## 2 Results

### 2.1 Cu‐Based Structure

**Figure 1:**
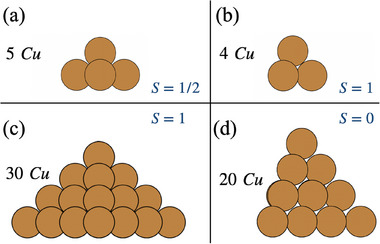
Cu tip models: a) Cu(100), b) Cu(111), c) Cu(100) pyramid model, and d) Cu(111) pyramid model, with their ground spin state.

The models of the Cu tip are pyramidal clusters, staking either Cu(100) or Cu(111) atomic planes. The smallest models, simulated both at the CASSCF and DFT level, consist of a minimal unit arrangement, square or triangle, respectively, plus the apex atom at the hollow site (**Figure** **1**a,b). The minimal Cu(100) model carries five 4s electrons leading to the expected 4s based‐MO wavefunctions governed by the C_4*v*_ symmetry: one doubly occupied bonding orbital, one unoccupied anti‐bonding orbital at high energy, and three intermediate states, of which one doubly occupied, one singly occupied, and one unoccupied. This electronic configuration (Figure S1 at the CASSCF level and Figure S2 at DFT level, Supporting Information) results in a ground spin state of *S* = 1/2. In the Cu(111) minimal model, the 4s semi‐occupied orbitals are delocalized following a C_3*v*_ symmetry, leading to one doubly occupied bonding orbital at low energy, two singly occupied non‐bonding orbitals at intermediate energy levels, and one unoccupied anti‐bonding orbital at high energy. This arrangement produces a ground spin state of *S* = 1.

To introduce more substrate effects, the tip models were expanded by adding two additional layers beneath each structure, creating pyramid models (Figure 1c,d). The calculations on these models are performed only at the DFT (PBE0‐D3) level. The Cu(100) pyramid model contains 30 Cu atoms and exhibits a ground spin state of *S* = 1, whereas the Cu(111) pyramid model contains 20 Cu atoms and has *S* = 0 in the ground state, indicative of a non‐magnetic substrate. The corresponding DFT spin‐polarized projected DOS reported in Figure S3 (Supporting Information). The general result following from the analysis of the four Cu substrate models is that a portion of their frontier bands is consistently attributed to the delocalized 4s states, which determine the spin state (the electronic structures are shown in Figures S1–S3, Supporting Information), and the apex Cu atom carries almost no spin polarization. This observation also suggests that the 4s electrons of bulkier STM tip delocalize and form a continuous metallic band across the Fermi level, further diminishing any apex spin polarization. Consequently, the pure Cu tip alone, without spin polarizarion, cannot provide the magnetic sensitivity required for precise spin detection, and requires the functionalization with magnetic elements, such as those from the 3d transition metal series, to impart a robust magnetic moment.

### 2.2 3d Transition Metal Atoms as Apexes of Cu (100) and (111) Substrates

Substituting the apex atom of a Cu STM tip with a different transition metal (TM) is far from a passive modification. Rather, it induces complex redox interactions with the underlying Cu substrate, yielding tip spin states that cannot be rationalized by simple atomic electronic configurations. These deviations are due to multiple interrelated effects. First, crystal field perturbations—trigonal on Cu(111) and tetragonal on Cu(100)—arise from second‐layer Cu atoms, altering the local electronic environment of the TM site. Second, shifts in the energy alignment between the TM 4s/3d orbitals and the Cu 4s valence band further modulate the interfacial electronic structure near the Fermi level. The degree of 3d(TM)–4s(Cu) hybridization emerges as a key factor in governing the final spin state of the tip apex.

**Figure 2:**
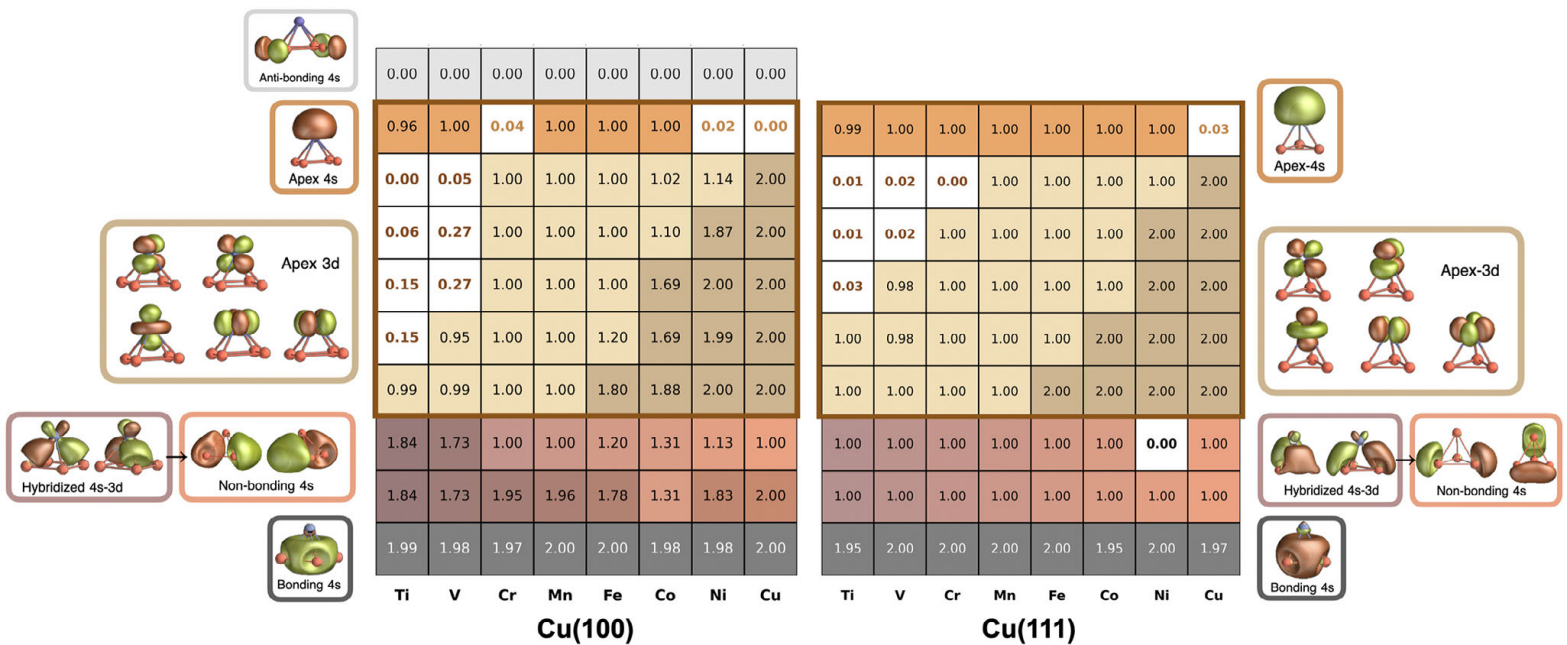
Ground state 4s and 3d orbital occupancy as predicted ab initio (CAS‐level with active space including the orbitals shown here except the anti‐bonding 4s orbital) for Cu(100) (left) and Cu(111) (right) tip models modified by the TM apex. The cell color corresponds to the frame color of the orbital illustrations on the side. The color shade, from darker to lighter, indicates larger to lower occupancy. The cells enclosed in the brown thicker frame are associated to the orbitals localized on the apex atom.

CAS calculations reveal a clear trend: as the atomic number increases from Ti to Cu, the energy of the TM 3d orbitals systematically decreases (**Figure** **2**). Initially lying above the non‐bonding Cu 4s “band”, the 3d orbitals descend below it across the series, reflecting a gradual reduction in the TM–Cu 4s energy mismatch. As a result, the degree of 3d(TM)–4s(Cu) orbital mixing diminishes from Ti to Cu, as evidenced by CAS‐derived molecular orbitals (Figures S6–S9, Supporting Information). This trend is also visually represented by a color gradient (dark to light pink) in Figure 2, and corroborated by DFT‐calculated orbital energy ladders for the structures TM@Cu(100) and TM@Cu(111) (Figures S5 and S8, Supporting Information, respectively).

Chemically, the non‐bonding Cu 4s states act as electron acceptors from the apex TM atom, leading to oxidation of the latter in both crystallographic orientations. Across the series from Ti to Ni at Cu(100), the apex atom is generally oxidized by one unit (M → M^+^), with two notable exceptions. For Ti and V@Cu(100), a double oxidation occurs (M → M^2+^). Interestingly, in these cases, the second electron is not removed from a 4s orbital but from a 3d one, yielding final electronic configurations of 4*s* ^1^3*d* ^1^ (Ti^2+^) and 4*s* ^1^3*d* ^2^ (V^2+^). Other examples underscore the variable acceptor role of the TM 3d orbitals: Cr^+^ adopts a 4*s* ^0^3*d* ^5^ configuration, while Ni^+^ yields 4*s* ^1^3*d* ^9^.

For the Cu(111) orientation, a more consistent trend is observed: All apex atoms from Ti to Co are singly oxidized (M → M^+^), with final 4*s* ^1^3*d* ^*n*^ configurations. Ni represents an exception, i.e., no redox event is observed, yet its final configuration still corresponds to 4*s* ^1^3*d* ^9^, suggesting a subtle interplay of hybridization and charge distribution unique to this case.

Assuming the Cu 4s band remains overall diamagnetic in bulk, a clear dependence of the total tip spin on the TM 3d electron count is evident. For TM@Cu(100) at CAS level, we obtain spin state minima for Ti (*S* = 1) and Ni (*S* = 1/2), and a maximum for Mn (*S* = 3), following the progression: Ti (*S* = 1), V (*S* = 3/2), Cr (*S* = 5/2), Mn (*S* = 3), Fe (*S* = 5/2), Co (*S* = 3/2), and Ni (*S* = 1/2). A similar but distinct trend is seen for TM@Cu(111): Ti (*S* = 3/2), V (*S* = 2), Cr (*S* = 5/2), Mn (*S* = 3), Fe (*S* = 5/2), Co (*S* = 1), and Ni (*S* = 1/2). These findings highlight the critical role of energy alignment, *i.e*., the chemical potential difference, between TM 3d/4s and Cu 4s states in determining the magnetic characteristics of the apex site. The choice of TM induces distinct oxidation states, electronic configurations, and spin multiplicities, offering a powerful strategy for engineering STM tips with customized spin‐dependent functionalities.

### 2.3 Structural and Electronic Properties of NiCp_2_‐Functionalized Tips

**Figure 3:**
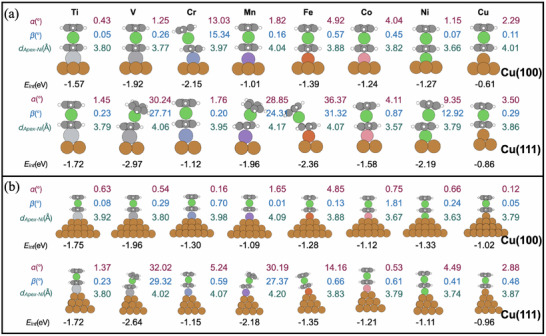
a) Optimized NiCp_2_@TM@Cu small pyramid models. b) Optimized geometries of the NiCp_2_@TM@Cu large pyramid models. In both panels, the upper row illustrates Cu(100)‐based models, the lower row Cu(111)‐based ones. The corresponding TM apex is indicated on the top. For each structure, the reported α angle (°) (red), β angle (°) (blue), the distance *d*(Apex − Ni) in Å (green), and the interaction energy between NiCp_2_ and the tip *E*_int_ in eV (black) are reported.

To further tune and improve the magnetic sensitivity of the copper‐based tips, we studied the structural and electronic properties of the functionalized TM@Cu ones, adding the NiCp_2_ molecule. All geometry optimizations are performed at the PBE0‐D3 level, from an initial configuration with NiCp_2_ vertically standing atop the apex atom. The electronic properties of the optimized structures are obtained at both the CASSCF and DFT levels for the minimal models, whereas for the larger ones only DFT is used. To describe the structure of the adsorbed NiCp_2_ we introduce three descriptors: the distance *d*_Apex − Ni_ between the apex TM and Ni in NiCp_2_; the tilting angle α between the Cu crystal plane and the plane of the topmost Cp ligands; the distortion angle β between the two Cp‐ligand planes (cf. Figure S15, Supporting Information). All the final structures are depicted in **Figure** **3**.

We observe that the apex–Ni distance and the interaction energy between NiCp_2_ and the tip change significantly depending on the Cu‐plane orientation and on the TM atop. However, for most minimal tip models, the molecule maintains an upright configuration with only minor deformation, that is, α < 5°, and β < 2°. Exceptions are Cr@Cu(100) and V, Mn, Fe, and Ni@Cu(111), when NiCp_2_ undergoes a notable distortion. In these cases, β reaches up to 30°, while the Cp ring closest to the tip remains almost coplanar with the surface planes. This behavior highlights a substrate‐ and element‐dependent modulation of molecular geometry (structural details in Table S2, Supporting Information).

A similar trend is observed after the optimization of NiCp_2_ adsorbed on the larger tip models, as shown in panel (b) of Figure 3. For all Cu(100) oriented structures, NiCp_2_ consistently retains an upright, undistorted geometry. The only minor deviation appears for Fe, where α is 5°, although β is 0.13°. The distances between Ni_Cp2_ (Ni ion belonging to Cp_2_) and the apex (3.63–4.09 Å) are in the same range as for the small‐tip models (3.66–4.04 Å). The only exception is Cr, for which the non‐distorted configuration is energetically favored on the larger model, whereas on the minimal model the topmost Cp ring bends with an angle β = 15.34°. All structural parameters are reported in Table S2 (Supporting Information). In Cu(111)‐based models, distorted geometries are obtained at the V and Mn apex, while NiCp_2_ remains undistorted when paired with Ti, Cr, Co, Ni, and Cu. In particular, in the Fe case, the molecule tilts rigidly but maintains parallel Cp rings, that is, β <1°, while α = 14.16°. The strongest interaction energies are obtained for V (2.64 eV) and Mn (2.18 eV), which suggests strong electronic coupling, associated with the distortion.

**Figure 4:**
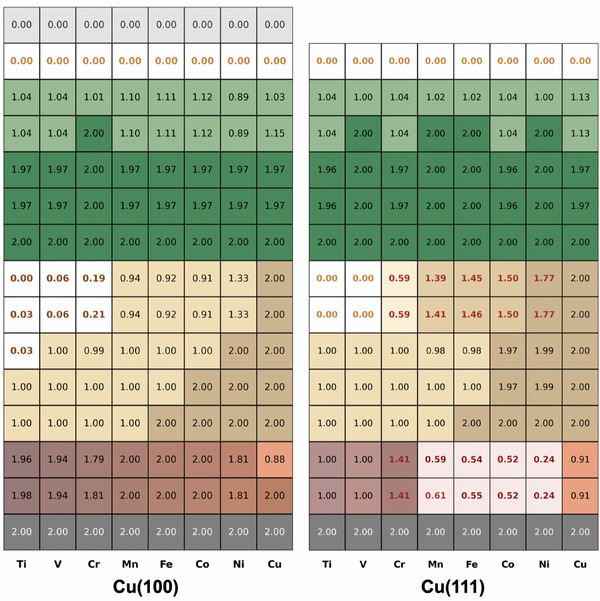
Ab initio predicted 4s and 3d orbitals occupancy calculated at the CASSCF level for the NiCp_2_@TM@Cu(100) (left) and NiCp_2_@TM@Cu(111) (right) series. The color code is the same as described in Figure 2, with the addition of the green cells representing the Ni‐3d orbitals. As above, the color shade, from darker to lighter, indicates larger to lower occupancy.

To identify clear and meaningful electrostructural correlations, we calculated the electronic properties of the functionalized minimal tip models at the CASSCF and at the DFT level for the whole NiCp_2_@TM@Cu(100) and NiCp_2_@TM@Cu(111) series. Building on the framework established for the bare TM@Cu(100) and TM@Cu(111) systems, we now extend the analysis to include the 3d orbitals of the NiCp_2_ (see **Figure** **4**). Despite structural similarities in the series, the electronic landscape is significantly more complex, particularly for NiCp_2_@TM@Cu(111) systems.

Focusing first on the NiCp_2_@TM@Cu(100) series, several processes emerge when compared to the non‐decorated tip counterparts. The first is an internal electron reorganization within the TM apex atom, such as 4s^1^ →3d^(*n* + 1)^ transitions, observed for Ti and V. For Mn through Co, a redox process analogous to that identified in TM@Cu(100) occurs, wherein electrons are transferred from TM 4s to the Cu 4s band. For Ni, which already exhibits this redox feature in the non‐decorated case, an additional transfer of approximately half an electron to the Cu 4s orbitals is observed. This results in a formal double oxidation (M^2+^) for most TMs, with Ni better described by a non‐integer oxidation state close to +1.5. In the case of a pure copper tip, no significant changes are found compared to the non‐decorated scenario; the apex Cu^+^ ion remains diamagnetic (3d^10^) and interacts weakly with NiCp_2_. Cr presents a more intricate behavior, showing triple oxidation (Cr^3+^), with one electron transferred to NiCp_2_, resulting in NiCp_2_^−^, and another to the Cu 4s band. In particular, this is the only case in which NiCp_2_ experiences a significant structural distortion.

The NiCp_2_@TM@Cu(111) series reveals a more complex electronic structure. Several electron transfer processes have been identified. These include internal 4s → 3d reorganizations within the TM apex atom (Ti and Co), 4s → Cu 4s band transfer (Cr), and 4s → NiCp_2_ transfers (V, Mn, Fe, and Ni). In parallel, a back‐donation from the Cu 4s band to the TM 3d orbitals is also observed from Mn through Ni. As a result of these complex rearrangements, the oxidation states remain formally unchanged (M^+^) for several TM (Ti, Mn, Fe, Cu), while Ni is best described as close to M^0^, Co is reduced (Co^+^ → Co^0^), and V and Cr are further oxidized to M^2+^. Moreover, NiCp_2_ is found to undergo reduction (Ni^2+^ → Ni^+^) in four systems: V, Mn, Fe, and Ni. Interestingly, these are also the systems that exhibit distortions in the NiCp_2_ geometry.

At first glance, the diversity of behaviors across the series appears difficult to rationalize. However, multiconfigurational CASSCF calculations provide useful insights. In all cases, the active space includes a doubly occupied bonding orbital (black lines in Figure S19, Supporting Information) and two unoccupied orbitals (gray lines in Figure S19, Supporting Information), corresponding to a localized TM 4s orbital and a delocalized Cu 4s antibonding combination. Two main types of orbital hybridization are identified. The first involves mixing between Cu 4s and TM 3d ($d_{\mathrm{xz}}$ and $d_{\mathrm{yz}}$) orbitals, prominent in systems with Ti, V, and Cr apex atoms (rosy‐brown lines in Figure S19, Supporting Information), and also seen in the Ni and Cu models. The second type features hybridization between the $d_{\mathrm{xz}}$ and $d_{\mathrm{yz}}$ orbitals of the Ni center in NiCp_2_ and those of the TM apex, most notably for Mn, Fe, Co, and Ni (khaki lines in Figure S19, Supporting Information). Both types of interaction are simultaneously active in NiCp_2_@Ni@Cu(100) and require a dedicated analysis (Section S4.5, Supporting Information), keeping the Cu apex model as a reference (Figure S18, Supporting Information).

For Cu(111)‐based systems, three distinct hybridization modes are identified in Figure S20 (Supporting Information). The first resembles that seen on Cu(100), involving the $d_{\mathrm{xz}}d_{\mathrm{yz}}$ orbitals and the 4s orbital of the TM apex. The second mode involves coupling between the NiCp_2_ $d_{\mathrm{xz}}d_{\mathrm{yz}}$ orbitals and the TM 4s orbital. The third involves interaction between the NiCp_2_ d orbitals, specifically $d_{x^{2}-y^{2}}$, $d_{z^{2}}$, and $d_{\mathrm{xy}}$, and those of the TM apex atom. This third mode is evident for V (Figure S27, Supporting Information), Mn (Figure S29, Supporting Information), and Fe (Figure S30, Supporting Information), and it correlates with the spin quenching in NiCp_2_ accompanying the structural distortion. Hybridization between TM $d_{\mathrm{xz}}d_{\mathrm{yz}}$, and Cu 4s is commonly observed, except for Cu, whose fully occupied 3d shell limits its participation in bonding.

Tables S3 and S4 (Supporting Information) summarize the electronic properties of the NiCp_2_‐tip models as predicted by DFT at two model sizes. The trends observed at this level of theory are in strong agreement with those obtained from CAS calculations for the small models. There exist some differences between the small and large pyramid models in a few cases, which will be discussed later.

Focusing specifically on NiCp_2_, all non‐distorted geometries retain a high‐spin *S* = 1 configuration, across all TM@Cu(100) and TM@Cu(111) systems and all model sizes. In contrast, on V@Cu(111) and on Mn@Cu(111), where β exceeds 20°, the NiCp_2_ spin state switches from *S* = 1 to *S* = 1/2. This reduction is driven by charge transfer from the tip to NiCp_2_, forming a NiCp_2_^−^ species (Ni^+^ 3d^9^ configuration). The spin moment of NiCp_2_ drops from approximately 1.9 µ_*B*_ to 1.1 µ_*B*_, and the strength of the interaction increases significantly, reaching 2.97 eV on V and 1.96 eV on Mn. This picture is also confirmed for the corresponding large models, where the interaction strengths are 2.64 eV (V) and 2.18 eV (Mn). The electronic rearrangement is well illustrated by the projected DOS in Figures S45 and S47 (Supporting Information), where the red arrow marks the shift of NiCp_2_ 3d states from the conduction to the valence regions. Being the point group associated to NiCp_2_ *D_5d_*, the reduction of NiCp_2_ leads to the electronic state degenerate ^2^ *E*_2*g*_, making it prone to Jahn–Teller effects and therefore to a stable distorted structure. The large Fe@Cu(111) model emerges as an outlier case since it features a large α angle but a small β, indicating a rigid tilt of the entire molecule without misalignment of the cyclopentadienyl rings.

### 2.4 Magnetic Properties Analysis

The most relevant property of spin‐polarized STM tips to guarantee high‐resolution detection of magnetic samples is the actual spin state emerging from the complex electronic interactions. To gain insight into this aspect, we have investigated the magnetic coupling between the TM apex atom and the NiCp_2_ molecule, by calculating both the ferromagnetic and antiferromagnetic configurations across the entire 3d series. The magnetic exchange interaction between NiCp_2_ and the TM apex exhibits a systematic evolution along the 3d row, transitioning from ferromagnetic coupling for early TMs (Ti, V) to antiferromagnetic coupling for later elements (Co, Ni). This trend correlates with the progressive filling of the TM 3d shell and is consistently reproduced by both DFT and multireference CASSCF calculations. However, significant discrepancies arise for the mid‐series elements Cr, Mn, and Fe, which are systems with a high number of unpaired electrons and closely spaced spin states that challenge single‐determinant approaches.

For Mn@Cu(111) and Fe@Cu(111), DFT predicts ferromagnetic coupling, with exchange constants (*J*) of –0.81 cm^−1^ and –4.97 cm^−1^, respectively. In contrast, multireference CAS (NEVPT2) calculations favor antiferromagnetic solutions, with corresponding *J* values of +0.48 cm^−1^ for Mn and 3.22 cm^−1^ for Fe (or +1.12 cm^−1^ and +0.92 cm^−1^, depending on the active space and orbital set). The case of Cr is particularly illustrative: the sign and magnitude of the exchange interaction depend on the geometry of the NiCp_2_ moiety. In the distorted configuration, the system exhibits ferromagnetic behavior, while in the non‐distorted geometry, the coupling becomes antiferromagnetic.

These discrepancies highlight the inherent limitations of single‐determinant DFT methods in reliably describing systems with pronounced multiconfigurational character and near‐degenerate spin states. Multireference approaches, such as CASSCF and NEVPT2, are capable of capturing subtle electronic effects and accurately resolving the ground‐state spin multiplicities. Such accuracy is indispensable not only for predicting the correct magnetic behavior, but also as a hint for initializing broken‐symmetry configurations in large‐scale DFT simulations of extended systems, where convergence and reliability often depend critically on the quality of the starting guess.

It is worth highlighting that an overall—and not necessarily expected—excellent agreement was obtained in determining the sign of the magnetic interaction across all systems. However, such agreement was not consistently achieved for the magnitude of the exchange coupling across all transition metals in both series, shown in Table S5 (Supporting Information). Very similar *J* values were computed for TM@Cu(111) systems from Ti to Mn. In contrast, for the TM@Cu(100) series, the *J* values obtained from the large models are generally smaller than those computed with the small model at the DFT level, and actually much closer to those calculated at CAS.

## 3 Discussion and Conclusions

The prototypical NiCp_2_@Cu@Cu(100) has been previously addressed by a few experimental and computational studies.^[^ ^6^ ^]^ This reference system has demonstrated distinct magnetic behaviors depending on the STM regime, i.e., tunneling versus contact. Specifically, a marked reduction in magnetic anisotropy has been reported when transitioning from the tunneling to the contact regime. In the tunneling regime, magnetic anisotropy values on the order of 30–40 cm^−1^ were observed. Combined with conductance and dI/dV spectroscopy, these results suggest a spin‐state transition from *S* = 1 to *S* = 1/2, which has been correlated with a structural rearrangement: the molecule adopts a tilted geometry in the tunneling regime, while the cyclopentadienyl rings align parallel to the Cu surface in the contact configuration. Several computational models have been considered, but most of them were restricted to a DFT+U approach, applying the Hubbard correction only to the Ni center. Our approach, combining hybrid functionals with dispersion corrections and, where needed, post‐HF treatments, is believed to be more accurate in describing multiple conformers coexisting within a few microelectronvolts and low‐lying spin states. Our results for the NiCp_2_@Cu@Cu(100) model identify the non‐tilted geometry as the most stable configuration, with an energy advantage of 24 meV over the tilted conformation. Experimentally, STM images^[^ ^21^ ^]^ have shown the coexistence of both geometries in the tunneling regime, with a higher occurrence of the tilted form. In this regime, a magnetic anisotropy parameter *D* ≈ 33 cm^−1^ was extracted from the conductance and spectroscopy data. Our calculations reproduce this value for both non‐tilted and moderately tilted geometries, specifically those characterized by a non‐zero α angle and negligible β distortion (see Figure S4). In contrast, for more distorted geometries (α and β both ≠ 0), the system adopts a lower spin state (*S* = 1/2) and exhibits a vanishing *D*, in line with experimental trends. These results indicate that a small tilt (α ≠ 0) is compatible with the experimental values *D*, provided that β remains close to zero. Indeed, to further prove that the parameter *D* is essentially a local property of NiCp_2_ and depends solely on the β values (*i.e*. removal of the degenerate electronic contribution to the Ni crystal field), we computed *D* for isolated NiCp_2_ molecules with α angles of 25°, 35°, and 45°, and with β of 0° (Figure S14, Supporting Information). The results show that *D* remains consistent with that of the non‐tilted structure (α = 0°), indicating that variations in α have a negligible impact on the magnetic anisotropy when the molecule retains mirror symmetry.

Concerning the models with modified apex atoms, our results indicate that the tendency of NiCp_2_ to adopt or not a distorted geometry is dictated by both the chemical identity of the apex TM atom and the packing structure of the underlying Cu substrate. The comparative analysis of TMs on the same surface highlights the electronic origin of these structural preferences. Among the small TM@Cu(100) tip models, the Cr and Mn apex cases (Figures S33 and S34, Supporting Information) serve as illustrative examples. In NiCp_2_@Cr@Cu(100), the molecule stabilizes in a distorted conformation (α = 13.03°, β = 15.34°), while in NiCp_2_@Mn@Cu(100), a non‐distorted structure (α = 1.82°, β = 0.16°) is energetically favored. The CASSCF molecular orbital analysis reveals that the distortion of NiCp_2_ is associated with the splitting of its *e*_*g*_ orbitals. One component is stabilized through interaction with an apex 3d orbital, while the other is lifted in energy due to hybridization. In the Cr case, this results in a net stabilization of the Ni 3d orbitals, lowering the energy of the distorted configuration. In contrast, for Mn, distortion increases the energy of the NiCp_2_ 3d orbitals, making the non‐tilted geometry energetically preferable. This effect is clearly reflected in the DFT‐derived average Ni 3d energy levels (Figure S35, Supporting Information), which decrease by 0.75 eV for Cr@Cu(100) and increase by 0.92 eV for Mn@Cu(100) with distortion. These shifts are directly correlated with the observed structural preferences and determine whether the distortion is energetically favorable.

**Figure 5:**
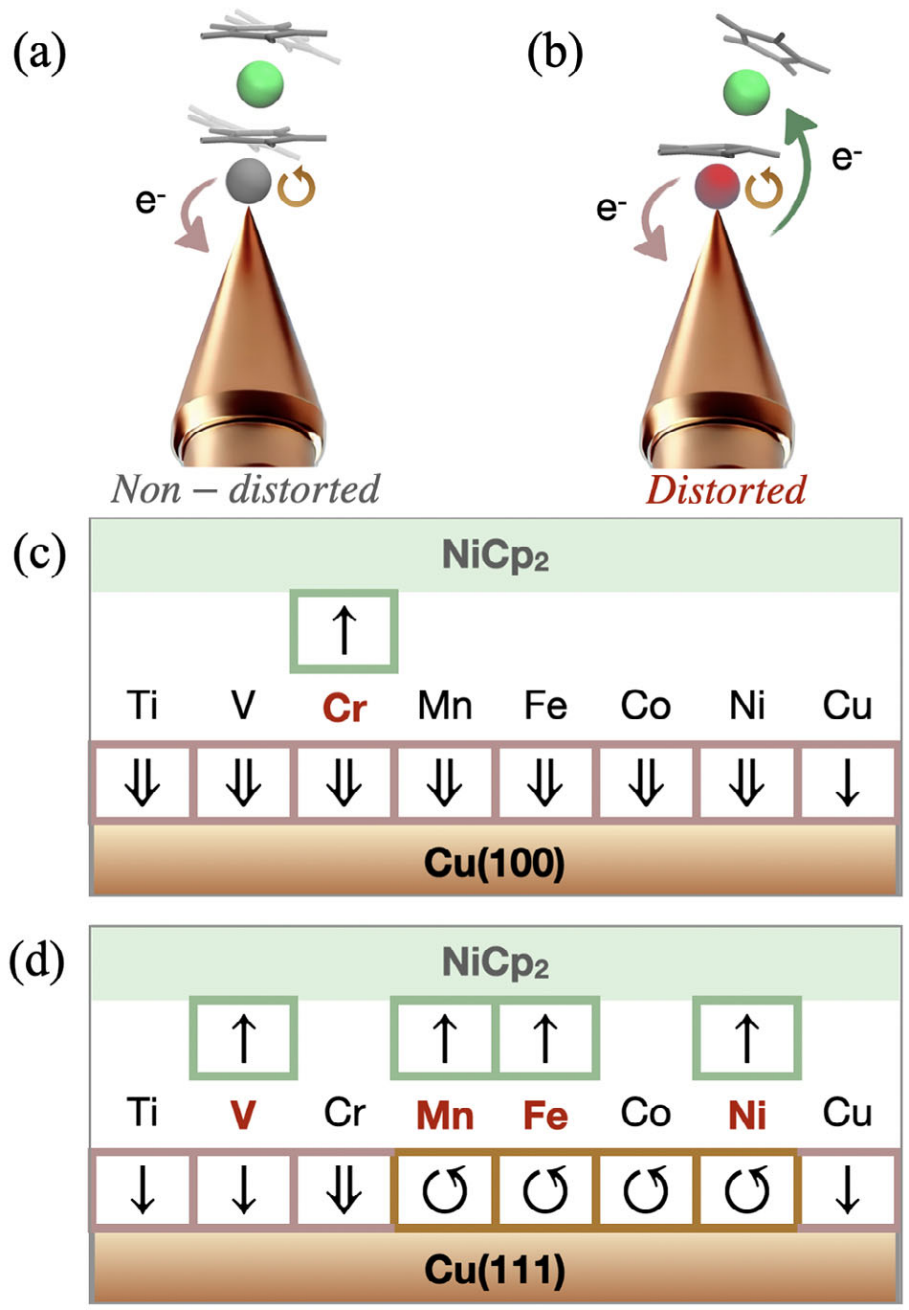
The scheme for the relationship between the geometries and electronic distributions on the NiCp_2_@TM@Cu small tip models. a) In the non‐distorted NiCp_2_ geometry including both non‐tilting and tilting cases, there are two types of changes in electronic structures: the pink arrow demonstrating the charge transfer from the apex atom to Cu substrate, the brown circle arrow representing the 3d‐4s re‐distribution. b) In the distorted NiCp_2_ with not parallel Cp rings, there is one more change that is transferring to NiCp_2_ from the apex and substrates, labeled by a green arrow. The scheme for each TM element as apex on c) Cu(100) and d) Cu(111) tip models, corresponding to the occupancy shown in Figure 4.

This trend extends to other TM@Cu systems, particularly on the Cu(111) substrate, where the distortion becomes favorable only when it lowers the energy of the NiCp_2_ frontier orbitals. In contrast, when distortion leads to destabilization of these orbitals, the molecule retains its symmetric, non‐tilted structure. Representative scans of the β angle for both distortion‐prone and distortion‐stable cases are provided in Section S4.8 (Supporting Information). These observations highlight the pivotal role of orbital alignment and electronic interactions in dictating NiCp_2_'s geometry, with the stabilization of 3d orbitals serving as the key driving force and how this process can be dependent by the β value. **Figure** **5** formally illustrates how geometric distortions relate to changes in electronic structure in small TM‐functionalized model tips. In distorted NiCp_2_ geometries, electrons are transferred from the apex TM atom and the Cu substrate to the NiCp_2_ 3d manifold (green arrows, Figure 5b). The interaction between the apex and the substrate primarily involves charge transfer from the TM apex to the Cu substrate (pink arrows in Figure 5a,b), consistent with the trends observed for nonfunctionalized tips. Furthermore, for Mn, Fe, Co, and Ni on Cu(111), internal rearrangements are observed within the 3d and 4s orbitals of TM (circular arrows in Figure 5b,d), reflecting more complex localized redistribution processes.

Overall, the size of the model plays a minor role, but for a few exceptions. For Fe/Ni@Cu(111) and Cr@Cu(100), the observed deviations can be rationalized in terms of differences in the electronic structure of the Cu substrate. Increasing the number of Cu atoms leads to a broader distribution of Cu 4s states, contributing to non‐bonding character. These 4s states form a continuous band near the Fermi level (Figure S3, Supporting Information) that can accommodate an excess electronic density, mitigating the need for charge transfer to NiCp_2_. As a result, electron accumulation remains on the Cu substrate rather than being injected into the molecule, preserving the planarity of NiCp_2_ and its original spin state. This effect is particularly evident for the Cu(100) pyramid models (30 atoms) and less so for the smaller Cu(111) (20 atoms). The density of states in Figures S52 and S53 (Supporting Information) illustrate well how the inclusion of an additional four‐layer slab further broadens the Cu 4s band. In these larger models, the NiCp_2_ moiety remains undistorted with parallel Cp rings, and no reduction to NiCp_2_^−^ is observed. Therefore, its intrinsic *S* = 1 spin state is preserved. However, in the presence of the Mn@Cu(111) and V@Cu(111) tips, NiCp_2_ adopts a distorted geometry characterized by large β values, accompanied by a reduction to NiCp_2_^−^. These findings are significant for two main reasons. First, from a methodological point of view, they demonstrate that the size of the substrate model and the degree of electronic delocalization and correlation (as treated by DFT vs. post‐Hartree–Fock methods) can critically influence molecular distortion and spin‐state quenching (determination of the true ground spin state). Second, and more scientifically relevant, the results establish a direct link between the structural distortion of NiCp_2_ and its reduction, which is predicted to be the ground state configuration in the case of Mn@ and V@Cu(111) tips. Furthermore, accurate energetic mapping of the various spin states and their ferro‐ or antiferromagnetic interactions provides valuable insights for the rational design and interpretation of IETS and STS experiments.

To reconcile the experimental dominance of tilted conformers with the predicted stability of the non‐tilted one, we conducted a forward–backward structural deformation simulation, using an Fe adatom as an STM tip model (see Section S5, Supporting Information). Starting from the most stable, non‐tilted geometry, we observed progressive molecular distortion as the tip approached the surface‐mimicking the transition from tunneling to contact regime. This distortion, involving simultaneous increases in α and β, led to a spin transition from *S* = 1 to *S* = 1/2, accompanied by a sharp reduction of the parameter *D* to nominal zero, in agreement with the experimental measurements. Upon reversing the tip–surface distance, the molecule partially restored its structure but retained a tilted configuration. This is consistent with experimental observations of NiCp_2_ molecules anchored with a tip and suggests that mechanical manipulation during tip formation can kinetically trap the molecule in a tilted metastable state.^[^ ^20^ ^]^ These findings support the reliability of our computational protocol and confirm that various NiCp_2_ conformers may coexist within a few meV. This has direct implications for the design and interpretation of future IETS and STS experiments involving NiCp_2_‐functionalized tips, and it provides a solid basis for extending our investigation to more complex NiCp_2_@TM@Cu(100)/(111) systems.

## 4 Computational Methods

Simulations of the small pyramid models were performed using the ORCA 5.0 software package.^[^ ^31^ ^]^ The geometries were optimized using hybrid density functional theory (hybrid‐DFT) at the PBE0 level, incorporating D3 dispersion corrections^[^ ^32^, ^33^ ^]^ to account for long‐range interactions. All elements were assigned the all‐electron def2‐TZVP basis set,^[^ ^34^ ^]^ with the def2/J^[^ ^35^ ^]^ and def2‐SVP/C^[^ ^36^ ^]^ auxiliary basis sets applied for integral approximations. A comparative analysis of the optimized geometries of NiCp_2_ in the gas phase, computed at different theoretical levels, is provided in Table S1 of Section S3 (Supporting Information). MO diagrams were derived from wavefunctions obtained from CASSCF calculations, based on geometries optimized at the PBE0‐D3 level, at the ground state. The active space was composed of five 3d orbitals of TM apex atom and five 3d orbitals of Ni (of NiCp_2_), and four 2π orbitals of Cp ligands (as shown in Figure S12, Supporting Information), and two or three non‐bonding (hybridized with 3d) 4s orbitals of Cu. The small tip models were constructed using a square arrangement of four Cu atoms to represent the Cu(100)‐based substrate (illustrated in Figure S15a, Supporting Information) and a triangular arrangement of three Cu atoms for the Cu(111)‐based substrate (depicted in Figure S15c, Supporting Information). To explore transition‐metal‐terminated tips, we substituted the Cu adatom with Ti, V, Cr, Mn, Fe, Co, or Ni.

Simulations of large pyramid models were carried out using the Quickstep module within the CP2K 2024 program package.^[^ ^37^ ^]^ These calculations employed Kohn–Sham density functional theory (DFT)^[^ ^23^, ^24^ ^]^ within a hybrid Gaussian and plane waves framework. The interactions between atomic cores and valence electrons were described using Goedecker–Teter–Hutter (GTH) pseudopotentials,^[^ ^38^ ^]^ while the molecular orbitals of the valence electron were expanded in Gaussian‐type basis sets. TZVP‐MOLOPT‐SR‐GTH basis sets were used for all the elements. Auxiliary basis sets included cFIT3 for light elements (H, C), cFIT9 for Cu atoms, and cFIT10 for the other transition metals. The exchange‐correlation functional used in the simulations was the PBE0 hybrid functional, accompanied by D3 dispersion corrections to account for long‐range interactions.^[^ ^39^ ^]^ The optimizations of the electronic structure were considered to have converged when the total energy difference reached an accuracy of 5 × 10^−7^ Hartree. Geometry optimizations for all structures were performed at the PBE0‐D3 functional level, using the Broyden–Fletcher–Goldfarb–Shanno (BFGS) algorithm^[^ ^40^ ^]^ with a convergence criterion for the maximum force of 1 × 10^−3^ Hartree Bohr^−1^. Spin alignment in the systems was initialized via the broken‐symmetry approach to define the spin states.^[^ ^41^, ^42^ ^]^ The reference NiCp_2_‐Cu(100) pyramid model comprised 30 Cu atoms arranged in a four‐layer square‐based pyramid, with a single NiCp_2_ molecule positioned at the apex, as depicted in Figure S15b (Supporting Information). Similarly, the Cu(111) pyramid model consisted of 20 Cu atoms configured in a four‐layer triangle‐based pyramid, with NiCp_2_ located at the apex, as shown in Figure S15d (Supporting Information). Both pyramid structures were centered within a cubic simulation cell measuring 30 × 30 × 30 Å^3^, with a 10 Å vacuum region on all sides to minimize interactions with periodic images.

The interaction energies (*E*_int_) between NiCp_2_ and the tip are obtained as

(1) $$E_{\text{int}}=E_{NiCp_{2}-\text{sub}}-\left(E_{NiCp_{2}}+E_{\text{sub}}\right)$$

where $E_{NiCp_{2}}$ and *E*_sub_ are the energies of molecule and substrate kept at the same atomic coordinates as in the complex. All the *E*_int_ are only calculated at PBE0‐D3 level of theory in ORCA and CP2K, for the small models and large models, respectively. The exchange energies (*E*_ex_) between NiCp_2_ and the tip are calculated as

(2) $$E_{\text{ex}}=E_{P}-E_{AP}$$

where *E*_*P*_ is the total energy of ferromagnetic coupling (parallel spin alignment), and *E*_*AP*_ denotes the total energy for antiferromagnetic coupling (anti‐parallel spin alignment).

The exchange constants *J* are calculated with the broken‐symmetry formalism as

(3) $$J_{12}=\frac{E_{S_{\max}}-E_{\mathrm{BS}}}{2S_{1}S_{2}}$$

where $E_{S_{\max}}$ is *E*_*P*_, and *E*_BS_ is *E*_*AP*_. The *S*_1_ and *S*_2_ are the local spins on the main magnetic centers, TM and Ni, riperspectively. The broken symmetry formalism has been shown to give reliable and accurate values in several other hybrid complex systems.^[^ ^43^ ^]^

## Conflict of Interest

The authors declare no conflict of interest.

## Supporting information

Supporting Information

## Data Availability

The data that support the findings of this study are available from the corresponding author upon reasonable request.

## References

[1] D. T. Pierce , Phys. Scr. 1988, 38, 291.

[2] O. Pietzsch , A. Kubetzka , M. Bode , R. Wiesendanger , Phys. Rev. Lett. 2000, 84, 5212. 10990905 10.1103/PhysRevLett.84.5212

[3] D. Wortmann , S. Heinze , P. Kurz , G. Bihlmayer , S. Blügel , Phys. Rev. Lett. 2001, 86, 4132. 11328113 10.1103/PhysRevLett.86.4132

[4] R. Wiesendanger , Rev. Mod. Phys. 2009, 81, 1495.

[5] M. Briganti , G. Serrano , L. Poggini , A. L. Sorrentino , B. Cortigiani , L. C. de Camargo , J. F. Soares , A. Motta , A. Caneschi , M. Mannini , F. Totti , R. Sessoli , Nano Lett. 2022, 22, 8626. 36256878 10.1021/acs.nanolett.2c03161 PMC9650780

[6] M. Ormaza , P. Abufager , B. Verlhac , N. Bachellier , M.‐L. Bocquet , N. Lorente , L. Limot , Nat. Commun. 2017, 8, 1974. 29215014 10.1038/s41467-017-02151-6 PMC5719446

[7] J. C. Oberg , M. R. Calvo , F. Delgado , M. Moro‐Lagares , D. Serrate , D. Jacob , J. Fernández‐Rossier , C. F. Hirjibehedin , Nat. Nanotechnol. 2014, 9, 64. 24317285 10.1038/nnano.2013.264

[8] P. Jacobson , M. Muenks , G. Laskin , O. Brovko , V. Stepanyuk , M. Ternes , K. Kern , Sci. Adv. 2017, 3, e1602060. 28439541 10.1126/sciadv.1602060 PMC5392040

[9] P. Jacobson , T. Herden , M. Muenks , G. Laskin , O. Brovko , V. Stepanyuk , M. Ternes , K. Kern , Nat. Commun. 2015, 6, 8536. 26456084 10.1038/ncomms9536 PMC4633813

[10] Q. Dubout , F. Donati , C. Wäckerlin , F. Calleja , M. Etzkorn , A. Lehnert , L. Claude , P. Gambardella , H. Brune , Phys. Rev. Lett. 2015, 114, 106807. 25815958 10.1103/PhysRevLett.114.106807

[11] C. J. Chen , Phys. Rev. Lett. 1990, 65, 448. 10042923 10.1103/PhysRevLett.65.448

[12] M. Nielinger , H. Baltruschat , ChemPhysChem 2003, 4, 1022. 14562452 10.1002/cphc.200300820

[13] G. Meyer , L. Bartels , S. Zöphel , E. Henze , K.‐H. Rieder , Phys. Rev. Lett. 1997, 78, 1512.

[14] J. Kröger , N. Néel , L. Limot , J. Condens. Matter Phys. 2008, 20, 223001.

[15] L. Meyer , M. Kögler , R. Henninger , N. Néel , J. Kröger , Small 2025, n/a, 2412703. 10.1002/smll.202412703 PMC12934386 40420615

[16] M. Kögler , N. Néel , L. Limot , J. Kröger , Nano Lett. 2024, 24, 14355. 39475061 10.1021/acs.nanolett.4c04075 PMC11566111

[17] B. Verlhac , N. Bachellier , L. Garnier , M. Ormaza , P. Abufager , R. Robles , M.‐L. Bocquet , M. Ternes , N. Lorente , L. Limot , Science 2019, 366, 623. 31672895 10.1126/science.aax8222

[18] G. Czap , P. J. Wagner , F. Xue , L. Gu , J. Li , J. Yao , R. Wu , W. Ho , Science 2019, 364, 670. 31097665 10.1126/science.aaw7505

[19] N. Bachellier , M. Ormaza , M. Faraggi , B. Verlhac , M. Vérot , T. Le Bahers , M.‐L. Bocquet , L. Limot , Phys. Rev. B 2016, 93, 195403.

[20] L. Zuo , Q. Zhuang , L. Ye , Y. Yan , X. Zheng , J. Phys. Chem. Lett. 2022, 13, 11262. 36448930 10.1021/acs.jpclett.2c03168

[21] M. Mohr , M. Gruber , A. Weismann , D. Jacob , P. Abufager , N. Lorente , R. Berndt , Phys. Rev. B 2020, 101, 075414.

[22] M. Radoń , Phys. Chem. Chem. Phys. 2014, 16, 14479. 24604025 10.1039/c3cp55506b

[23] P. Hohenberg , W. Kohn , Phys. Rev. 1964, 136, B864.

[24] W. Kohn , L. J. Sham , Phys. Rev. 1965, 140, A1133.

[25] M. Swart , Inorg. Chim. Acta. 2007, 360, 179.

[26] S. B. Song , Z. Wang , J. Li , R. Q. Wu , Phys. Rev. B 2022, 105, 064415.

[27] B. O. Roos , in Advances in Chemical Physics , (Ed.: K. P. Lawley ), vol. 69, 1 edition, Wiley, 1987, pp. 399–445.

[28] C. Angeli , R. Cimiraglia , S. Evangelisti , T. Leininger , J.‐P. Malrieu , J. Chem. Phys. 2001, 114, 23. 10.1063/1.2911699 18465905

[29] C. Angeli , R. Cimiraglia , J.‐P. Malrieu , J. Chem. Phys. 2002, 117, 9138.

[30] C. Angeli , R. Cimiraglia , J.‐P. Malrieu , Chem. Phys. Lett. 2001, 350, 297.

[31] F. Neese , WIREs Comput. Mol. Sci. 2022, 12, e1606.

[32] S. Grimme , J. Antony , S. Ehrlich , H. Krieg , J. Chem. Phys. 2010, 132, 154104. 20423165 10.1063/1.3382344

[33] S. Grimme , S. Ehrlich , L. Goerigk , J. Comput. Chem. 2011, 32, 1456. 21370243 10.1002/jcc.21759

[34] F. Weigend , R. Ahlrichs , Phys. Chem. Chem. Phys. 2005, 7, 3297. 16240044 10.1039/b508541a

[35] F. Weigend , Phys. Chem. Chem. Phys. 2006, 8, 1057. 16633586 10.1039/b515623h

[36] A. Hellweg , C. Hättig , S. Höfener , W. Klopper , Theor. Chem. Acc. 2007, 117, 587.

[37] T. D. Kühne , M. Iannuzzi , M. Del Ben , V. V. Rybkin , P. Seewald , F. Stein , T. Laino , R. Z. Khaliullin , O. Schütt , F. Schiffmann , D. Golze , J. Wilhelm , S. Chulkov , M. H. Bani‐Hashemian , V. Weber , U. Borštnik , M. Taillefumier , A. S. Jakobovits , A. Lazzaro , H. Pabst , T. Müller , R. Schade , M. Guidon , S. Andermatt , N. Holmberg , G. K. Schenter , A. Hehn , A. Bussy , F. Belleflamme , G. Tabacchi , et al., J. Chem. Phys. 2020, 152, 194103. 33687235 10.1063/5.0007045

[38] S. Goedecker , M. Teter , J. Hutter , Phys. Rev. B 1996, 54, 1703. 10.1103/physrevb.54.1703 9986014

[39] J. Moellmann , S. Grimme , J. Phys. Chem. C 2014, 118, 14.

[40] J. D. Head , M. C. Zerner , Chem. Phys. Lett. 1985, 122, 264.

[41] L. Noodleman , J. Chem. Phys. 1981, 74, 5737.

[42] A. Bencini , F. Totti , J. Chem. Theory Comput. 2009, 5, 144. 26609828 10.1021/ct800361x

[43] J. A. Burgess , L. Malavolti , V. Lanzilotto , M. Mannini , S. Yan , S. Ninova , F. Totti , S. Rolf‐Pissarczyk , A. Cornia , R. Sessoli , S. Loth , Nat. Commun. 2015, 6, 8216. 26359203 10.1038/ncomms9216 PMC4579601

