# Supplementary material for: Atomistic Simulation‐Driven Design of STM Tips for NiCp2 Adsorption and Spin‐State Modulation
Source: Small. 2025 Oct 22;21(52):e08320. doi: 10.1002/smll.202508320 (PMC12747638; doi:10.1002/smll.202508320)
Supplement: Supplementary file 1 — Supporting Information [file SMLL-21-e08320-s001.pdf]

NANO · MICRO
small

Supporting Information

for *Small*, DOI 10.1002/smll.202508320

Atomistic Simulation-Driven Design of STM Tips for $NiCp_2$ Adsorption and Spin-State Modulation

Nanchen Dongfang, Federico Totti and Marcella Iannuzzi**

Supporting Information: Atomistic Simulation-Driven Design of STM Tips for $NiCp_2$ Adsorption and Spin-State Modulation

Nanchen Dongfang Federico Totti Marcella Iannuzzi**

Nanchen Dongfang, Prof. Dr. Marcella Iannuzzi
Department of Chemistry, University of Zurich, 8057 Zurich, Switzerland
Email Address: marcella.iannuzzi@chem.uzh.ch
Prof. Dr. Federico Totti
Dipartimento di Chimica "Ugo Schiff" & INSTM RU, Università degli Studi di Firenze, Via della Lastruccia 3, 50019 Sesto Fiorentino (FI), Italy
Email Address: federico.totti@unifi.it

Contents

S.1 Electronic structures for different kinds of Cu substrates 2

S.2 TM-apex on Cu substrates 4
S.2.1 TM-apex on Cu(100) substrates . . . 4
S.2.2 TM-apex on Cu(111) substrates . . . 6

S.3 Isolated $NiCp_2$ properties 8
S.3.1 The MOs of $NiCp_2$ at CASSCF . . . 8
S.3.2 $NiCp_2$ in gas phase optimized at different levels of theory . . . 8
S.3.3 The calculations of D parameters for different $NiCp_2$ geometries . . . 9

S.4 $NiCp_2$@TM@Cu complexes 12
S.4.1 The structural information for $NiCp_2$@TM@Cu based models . . . 12
S.4.2 The sum of magnetic and electronic properties for $NiCp_2$@TM@Cu based models . . . 14
S.4.3 The orbital energy levels for $NiCp_2$@TM@Cu(100) tip models at PBE0-D3 level of theory . 17
S.4.4 The orbital energy levels for $NiCp_2$@TM@Cu(111) tip models at PBE0-D3 level of theory . 18
S.4.5 CASSCF MOs for $NiCp_2$@TM@Cu tip models . . . 19
S.4.6 Comparisons between non-distorted and distorted geometries and electronic structure . . . 29
S.4.7 The PDOS for the pyramid models . . . 33
S.4.8 The relationship between β values and molecular spin . . . 44

S.5 The tilted behavior for $NiCp_2$@Cu(100)-terminated tip at different DFT-based functionals 47
S.5.1 Comparison between the tilted and non-tilted $NiCp_2$@Cu(100) tips at different levels of theory 50
S.5.2 Computational details . . . 50

S.1 Electronic structures for different kinds of Cu substrates

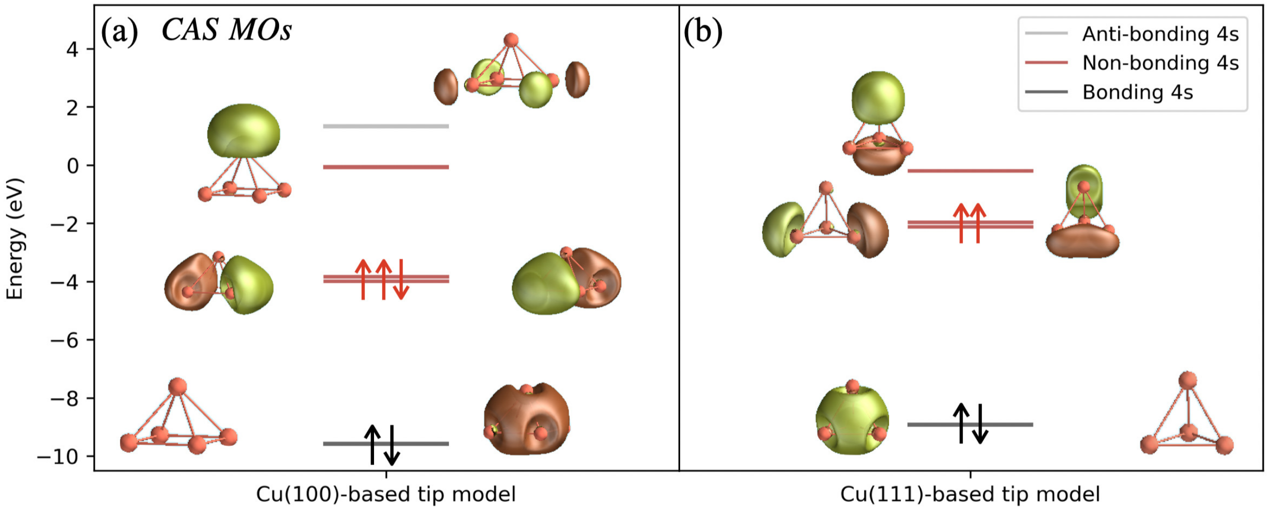

Figure S1: The CASSCF MOs for (a) Cu(100) and (b) Cu(111) packing tip models.

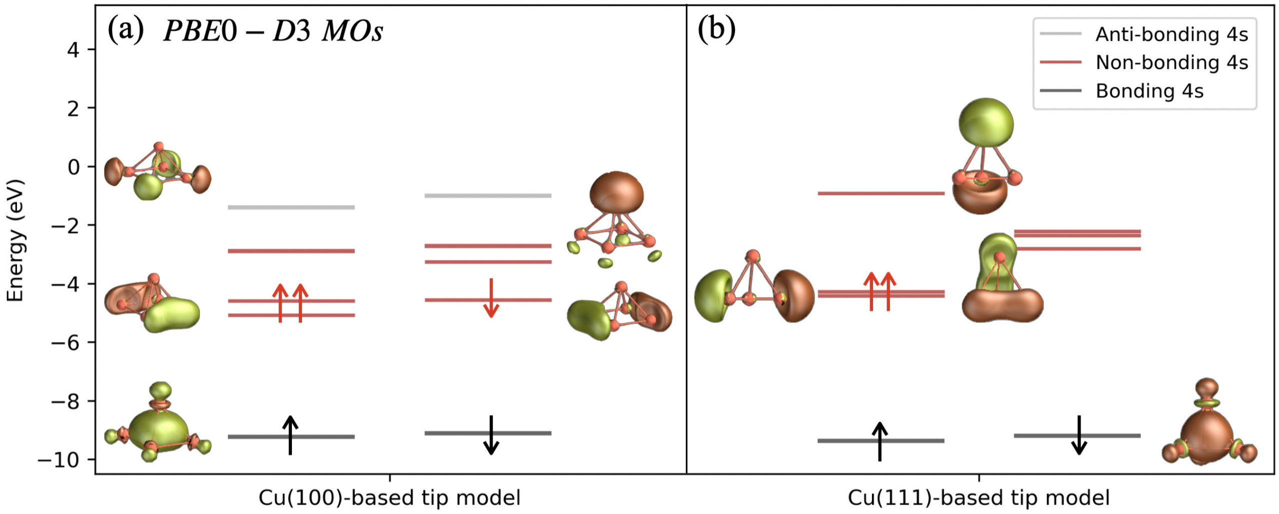

Figure S2: The DFT MOs (PBE0-D3) for (a) Cu(100) and (b) Cu(111) packing tip models.

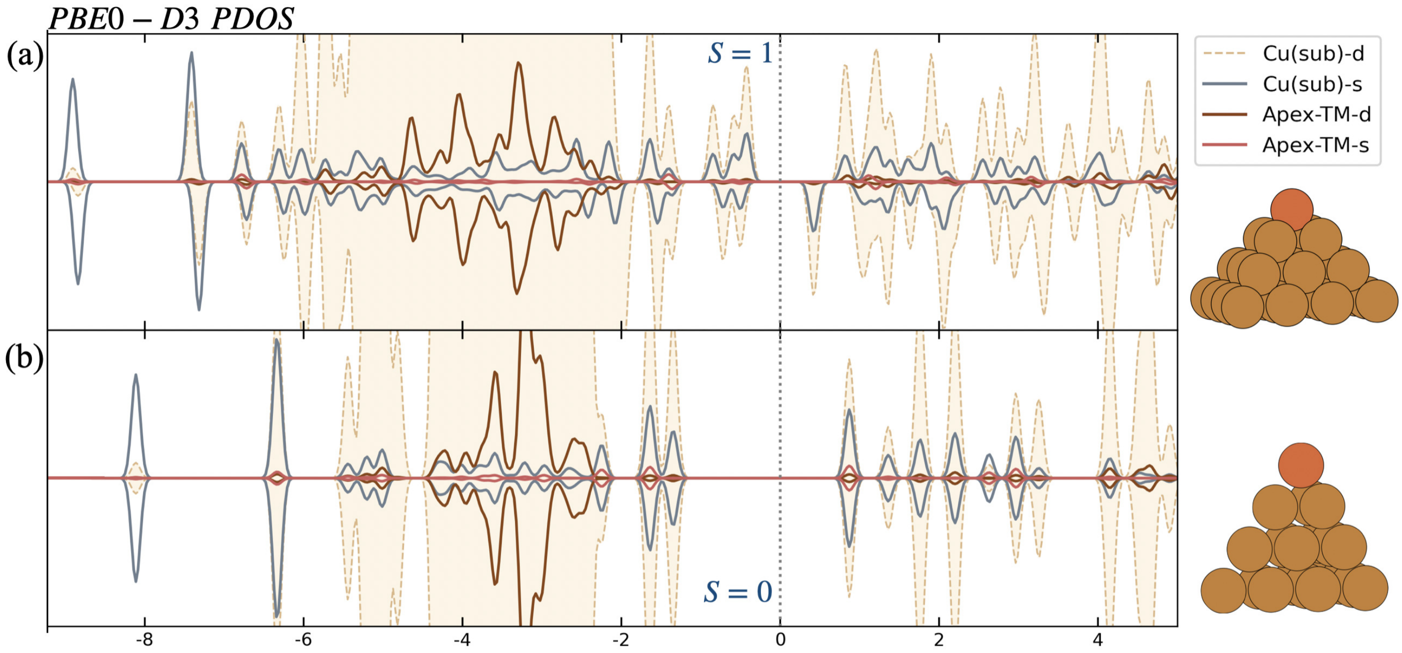

Figure S3: Projected DOS (PBE0-D3) for (a) Cu(100) and (b) Cu(111) packing pyramid models, with corresponding structures on the right side.

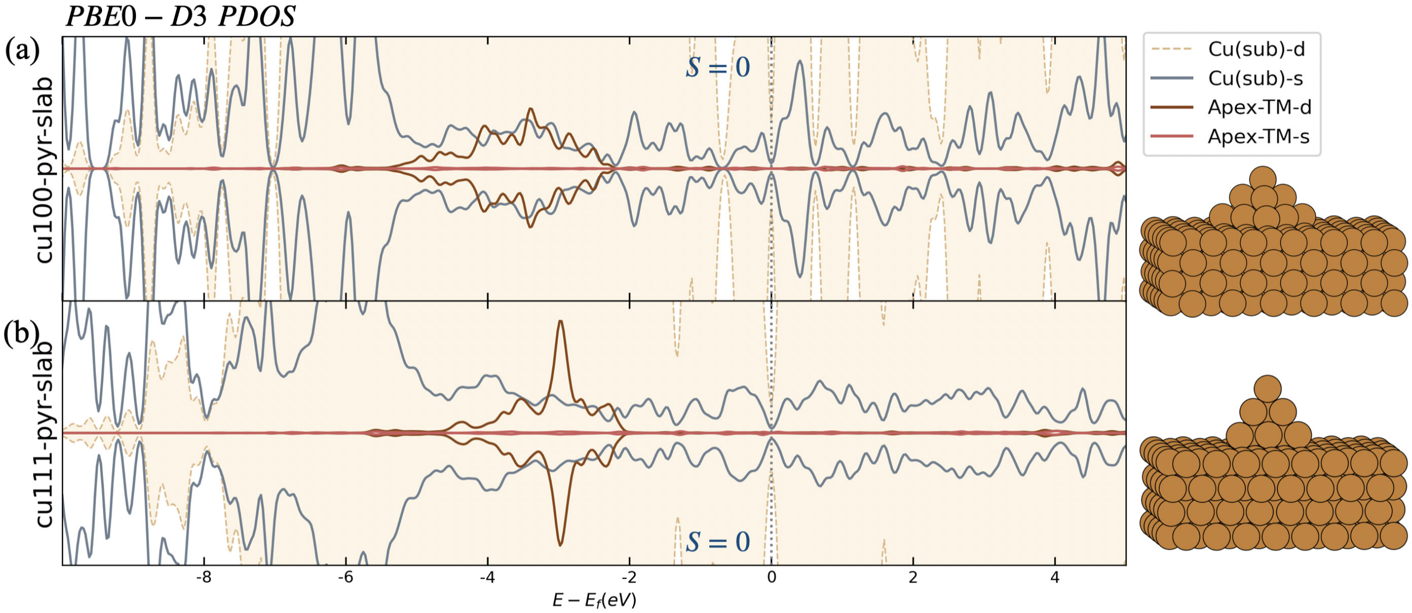

Figure S4: Projected DOS (PBE0-D3) for (a) Cu(100) and (b) Cu(111) packing pyramid placed on top of a 4-layer Cu slab with same orientation, with corresponding structures on the right side.

S.2 TM-apex on Cu substrates

S.2.1 TM-apex on Cu(100) substrates

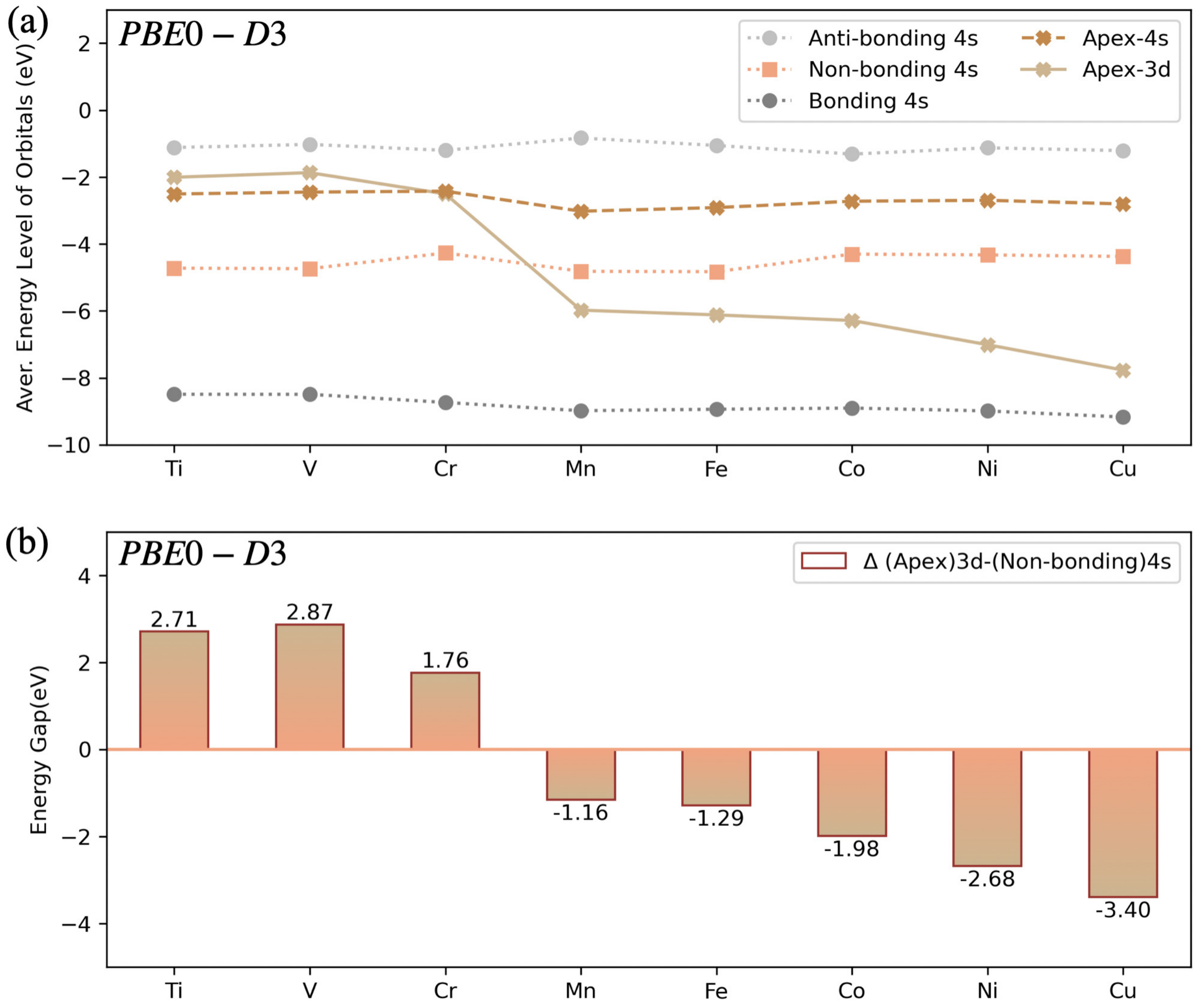

Figure S5: The energy levels (at PBE0-D3 level of theory) of 4s and 3d orbitals for a series of TM (Ti, V, Cr, Mn, Fe, Co, Ni) on Cu(100) small tips, with the Cu@Cu(100) as a reference in the end.

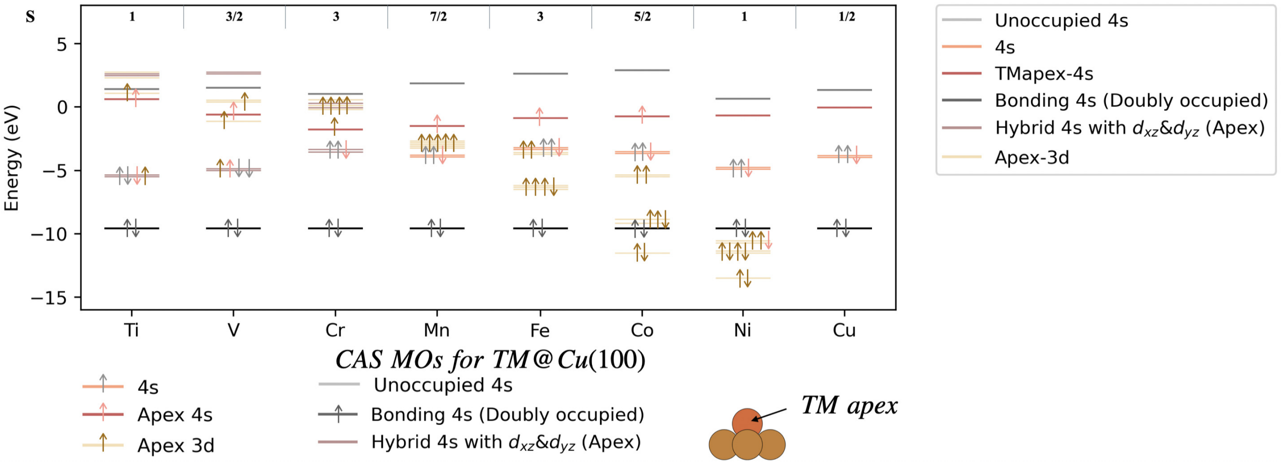

Figure S6: CASSCF MOs diagrams for a series of TM (Ti, V, Cr, Mn, Fe, Co, Ni) on Cu(100) small tips, with the Cu@Cu(100) as a reference in the end.

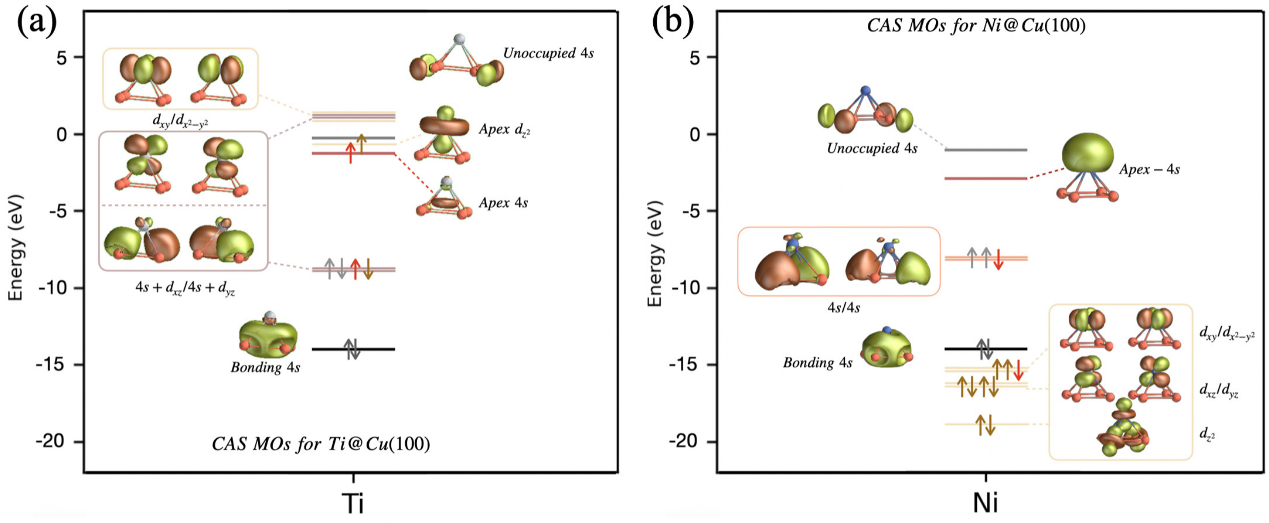

Figure S7: The CASSCF MOs diagrams for (a)Ti@Cu(100) and (b) Ni@Cu(100) small tips.

S.2.2 TM-apex on Cu(111) substrates

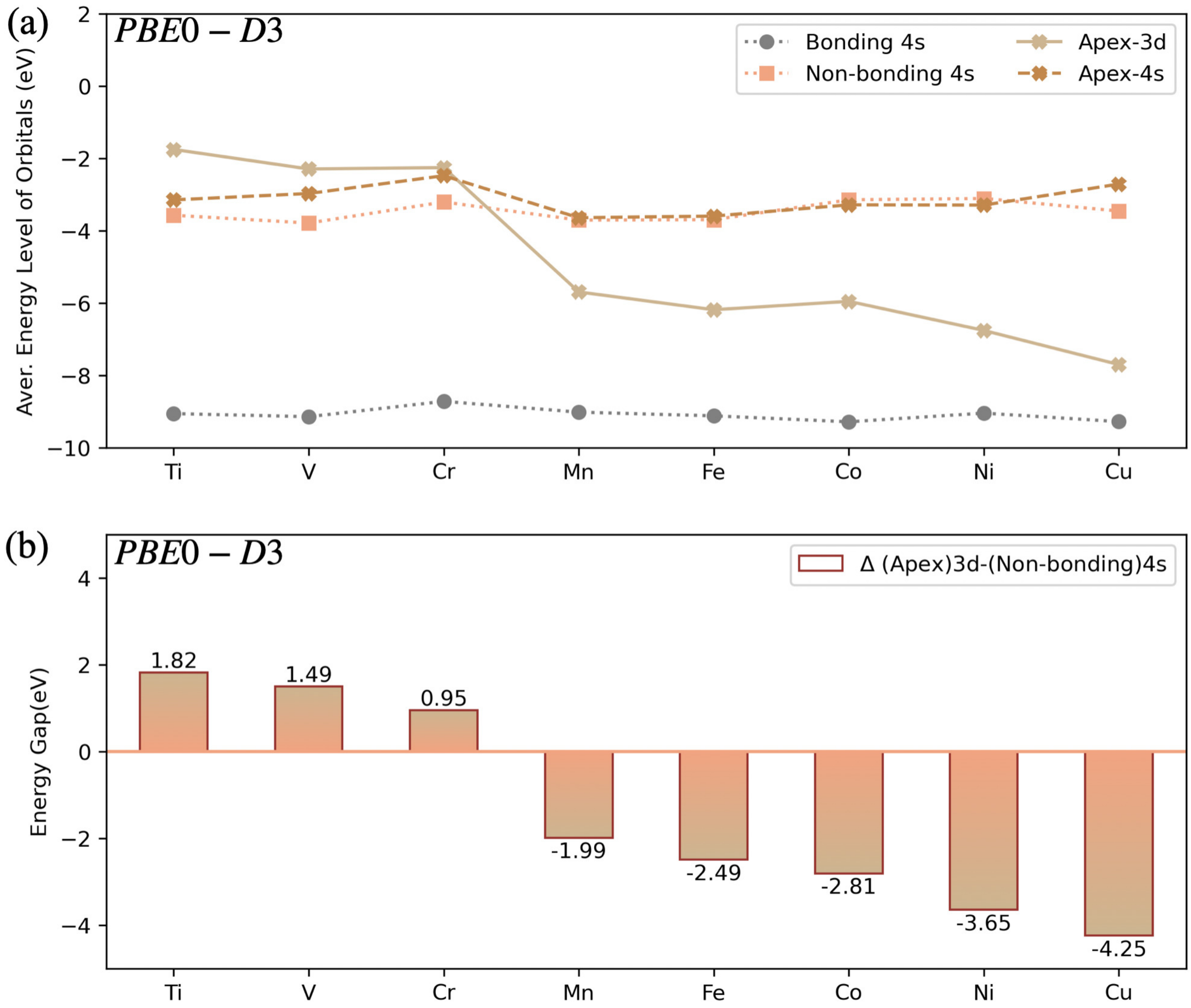

Figure S8: The energy levels (at PBE0-D3 level of theory) of 4s and 3d orbitals for a series of TM (Ti, V, Cr, Mn, Fe, Co, Ni) on Cu(111) small tips, with the Cu@Cu(100) as a reference in the end.

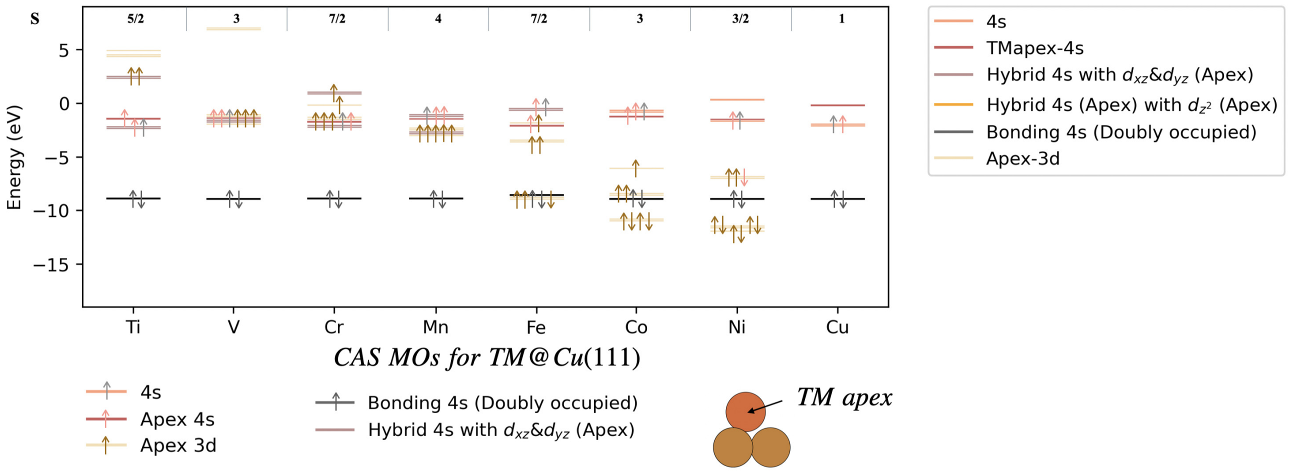

Figure S9: CASSCF MOs diagrams for a series of TM (Ti, V, Cr, Mn, Fe, Co, Ni) on Cu(100) small tips, with the Cu@Cu(111) as a reference in the end.

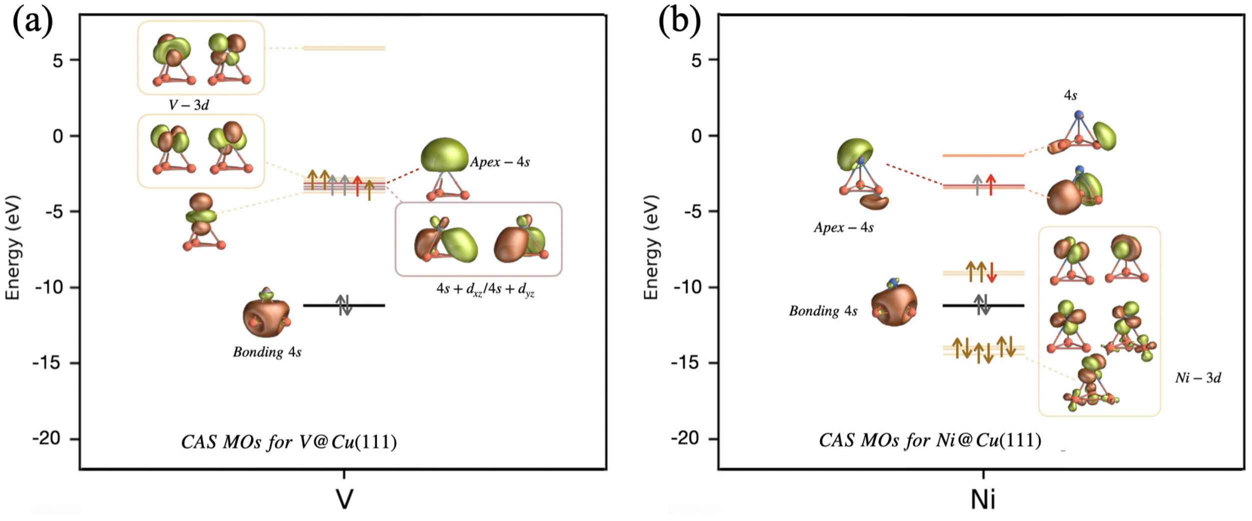

Figure S10: The CASSCF MOs diagrams for (a)V@Cu(111) and (b) Ni@Cu(111) small tips.

S.3 Isolated $NiCp_2$ properties

S.3.1 The MOs of $NiCp_2$ at CASSCF

$NiCp_2$ possesses a triplet $S = 1$ ground state and is an open-shell complex, with an electronic configuration of $(a_{1g})^2(e_{2g})^4(e_{1g})^2$. The $4s^2$ electrons of Ni are donated to the Cp rings by coupling with an unpaired electron present there, resulting in a formal Ni^{2+} oxidation state and two Cp^- ligands.

The cyclopentadienyl anion ($C_5H_5{}^-$), denoted as Cp^-, is the deprotonated form of cyclopentadiene (Cp). Its molecular orbital (MO) diagram is shown in Figure S11. Cp^- contains no unpaired electrons and is inherently a relatively stable ligand. In most CASSCF simulations of $NiCp_2$ [1, 2], the active space typically includes only the five 3d orbitals of Ni along with eight electrons (Ni-3d^8), denoted as CAS(8,5). The π-orbitals of the Cp ligands are often excluded, as they are regarded as core orbitals.

In this study, we investigate the interaction of $NiCp_2$ with various substrates. Some substrates induce structural distortions in the Cp rings, such as tilting and mismatching, involving conjugated π orbitals interacting and hybridizing with the d orbitals of Ni and other transition metals (TM). Figure S12 presents the potential reactive MOs of $NiCp_2$ derived from calculations using CAS(8,5), CAS(16,9), and CAS(20,15). The larger active spaces incorporate conjugated π-orbitals originating from the C-p_z orbitals of the Cp rings.

These calculations reveal that the two 1π orbitals mix and split into two new orbitals that have minimal interaction with the central Ni atom. A similar phenomenon is observed for the four 3π orbitals, which are located at significantly higher energy levels, distant from the frontier occupied states. Notably, the 2π orbitals are mixed with Ni d_{xz} and d_{yz} orbitals, suggesting a strong correlation with adsorption and interaction processes involving substrates. Consequently, in our CASSCF simulations, the active space includes the four 2π orbitals from the two Cp rings, rather than the complete set of ten π-orbitals.

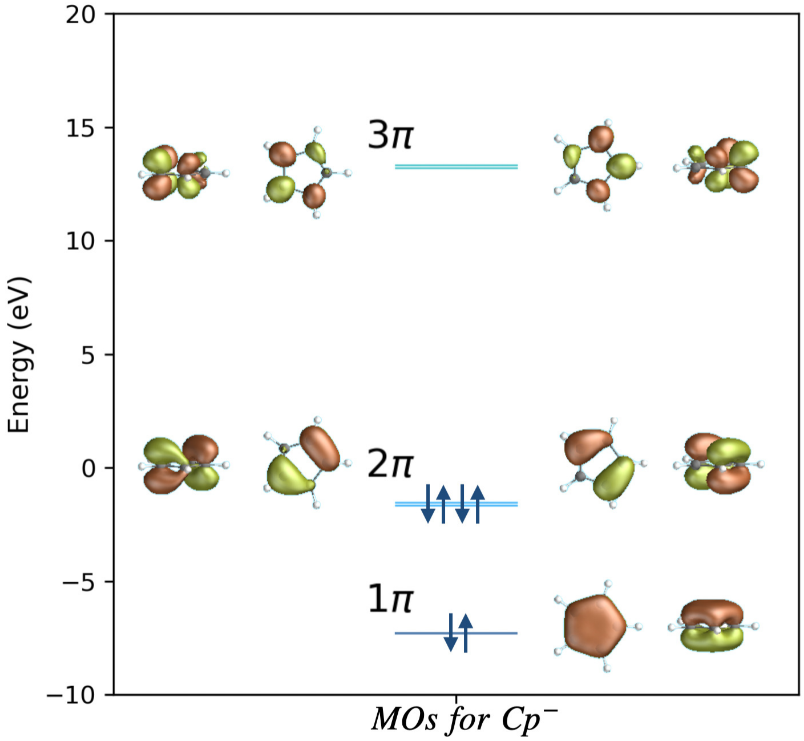

Figure S11: The CASSCF MOs diagram for Cp^- frontier orbitals.

S.3.2 $NiCp_2$ in gas phase optimized at different levels of theory

Table S1 summarizes the structural parameters and simulated D values (in cm^{-1}) for $NiCp_2$ derived from geometries optimized using kinds of methods. These include wavefunction-based approaches such

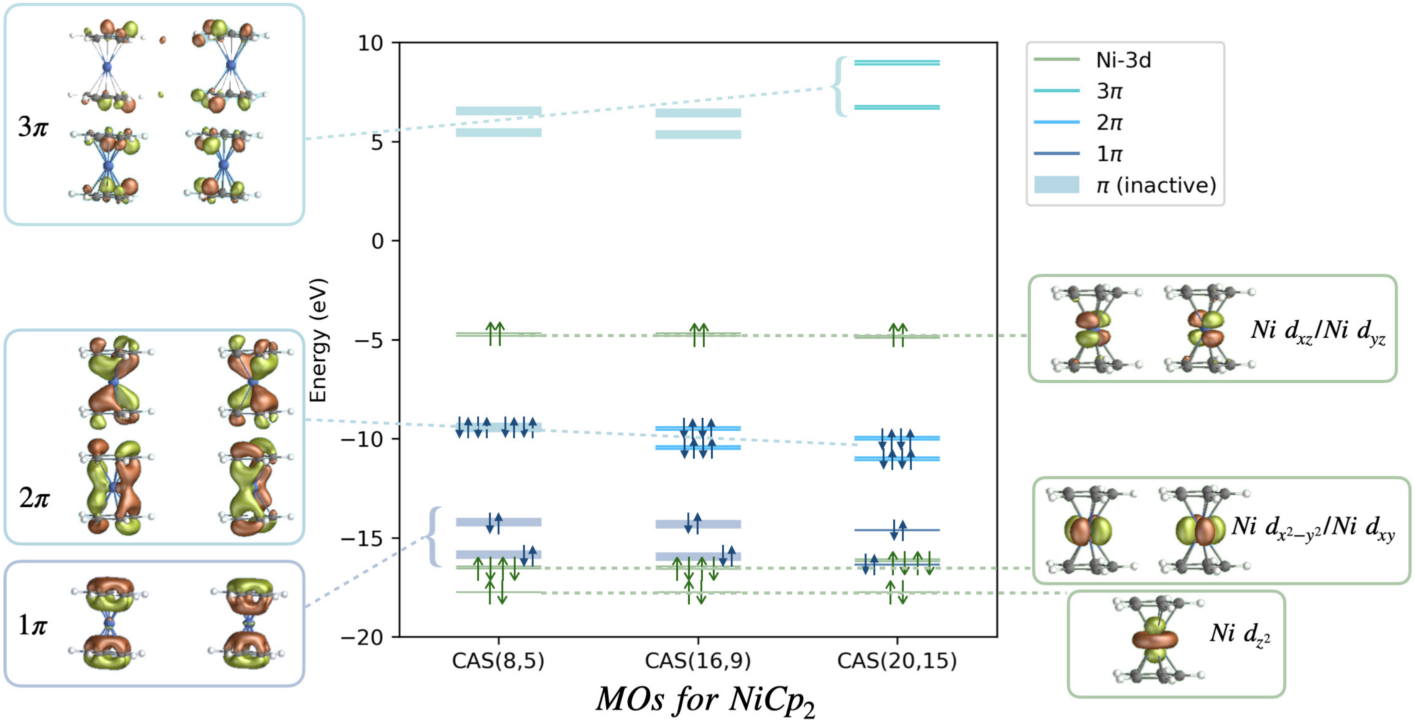

Figure S12: CASSCF MOs diagrams for a single $NiCp_2$ molecule, presented from left to right: CAS(8,5) – comprising eight 3d electrons in five 3d orbitals; CAS(16,9) – additionally incorporating four 2π orbitals with eight electrons distributed among them; CAS(20,15) – encompassing all five π-orbitals from the two Cp rings (ten π-orbitals in total) alongside the five 3d orbitals of Ni.

as Hartree-Fock (HF) and CASSCF, as well as DFT with functionals such as PBE, PBE0, PBE-D3, and PBE0-D3 in ORCA, and PBE-D3 and PBE0-D3 in CP2K.

When compared to experimentally detected data [3], HF and CASSCF methods predict larger Ni-to-Cp distances, making these geometries unreliable. The Ni-C bond lengths are overestimated by approximately $+0.15$ Å, while the C-C and C-H bond lengths are underestimated by -0.21 Å and -0.012 Å, respectively. Moreover, the D parameters computed from these geometries using CASSCF and NEVPT2 are substantially larger than those obtained from other methods in this work and previous simulations [2].

In contrast, DFT-based functionals outperform wavefunction-based methods in accurately reproducing experimental structures of $NiCp_2$. The deviations from experimental bond lengths are consistently less than 0.015 Åacross all DFT results. Dispersion corrections (D3) have minimal impact, slightly favoring shorter Ni-C and C-H bonds and longer C-C bonds. Among DFT methods, PBE provides particularly accurate C-C bond lengths, closer to experimental values compared to PBE0.

The simulated D parameters for DFT-optimized structures fall within 37.21 to 39.04 cm^{-1} using NEVPT2-CAS(8,5), and 64.41 to 67.40 cm^{-1} using CAS(8,5). These values show a clear correlation with the distance between Ni and the Cp planes, as illustrated in Figure S13. The larger distances of Ni-Cp correspond to higher values of D, a trend that is especially evident when comparing optimized geometries using HF and CASSCF.

The perference of PBE0 can be ascribed to its incorporation of HF exchange, which enhances its ability to describe localized 3d electrons in transition metals. Consequently, geometries optimized using PBE0 are expected to perform well in HF-based methods such as CASSCF, which are essential to accurately describe near-degenerate spin states using multireference determinants.

S.3.3 The calculations of D parameters for different $NiCp_2$ geometries

The geometries of $NiCp_2$ were derived from TM-Cu tip models and pyramid models by isolating the $NiCp_2$ component. These geometries were used to calculate D parameters (in cm^{-1}) and to explore the relationship between D parameters and structural variations, as illustrated in Figure S14. The D for

Table S1: Structural details of isolated $NiCp_2$ in the gas phase, optimized at various levels of theory using the ORCA and CP2K packages (CP2K optimizations are indicated). The lower section presents the D parameter values for $NiCp_2$, calculated using CAS(8,5) and CAS(16,9) in ORCA, based on the corresponding optimized geometries listed above.

	Exp.	HF	CAS	PBE	PBE0	PBE-D3	PBE0-D3	PBE-D3(cp2k)	PBE0-D3(cp2k)
Bond length Ni-C (Å)	2.196[1]	2.323	2.336	2.198	2.196	2.183	2.185	2.198	2.194
Bond length C-C (Å)	1.430[1]	1.409	1.409	1.428	1.416	1.429	1.417	1.428	1.416
Bond length C-H (Å)	1.083[1]	1.071	1.071	1.087	1.080	1.086	1.078	1.087	1.080
Distance between Ni to Cp plane (Å)		1.990	2.005	1.832	1.836	1.821	1.834	1.814	1.823
D parameter (cm^{-1})									
CAS(8,5) by NEVPT2	70.1[2]	56.85	58.88	39.04	39.02	37.95	38.74	37.21	37.64
CAS(16,9) by NEVPT2		61.83	64.05	42.36	42.31	41.21	42.03	40.42	40.88
CAS(8,5)	40.1[2]	95.43	98.45	67.40	67.37	65.63	66.91	64.41	65.11
CAS(16,9)		92.90	95.92	65.22	65.18	63.48	64.72	62.29	62.96

[1] The strusture of $NiCp_2$ in gas phase was determined at 110 $°C$ by electron diffraction [3].
[2] Experimental D parameters for $NiCp_2$ in the range 25.6 ± 3.0 ... 33.6± 0.3 cm^{-1} [4–6]. The simulated D parameters are 70.1 by CASSCF and 40.1 by NEVPT2, with the def2-TZVP basis set, using a molecular structure obtained from B2LYP/def2-TZVP. [2]

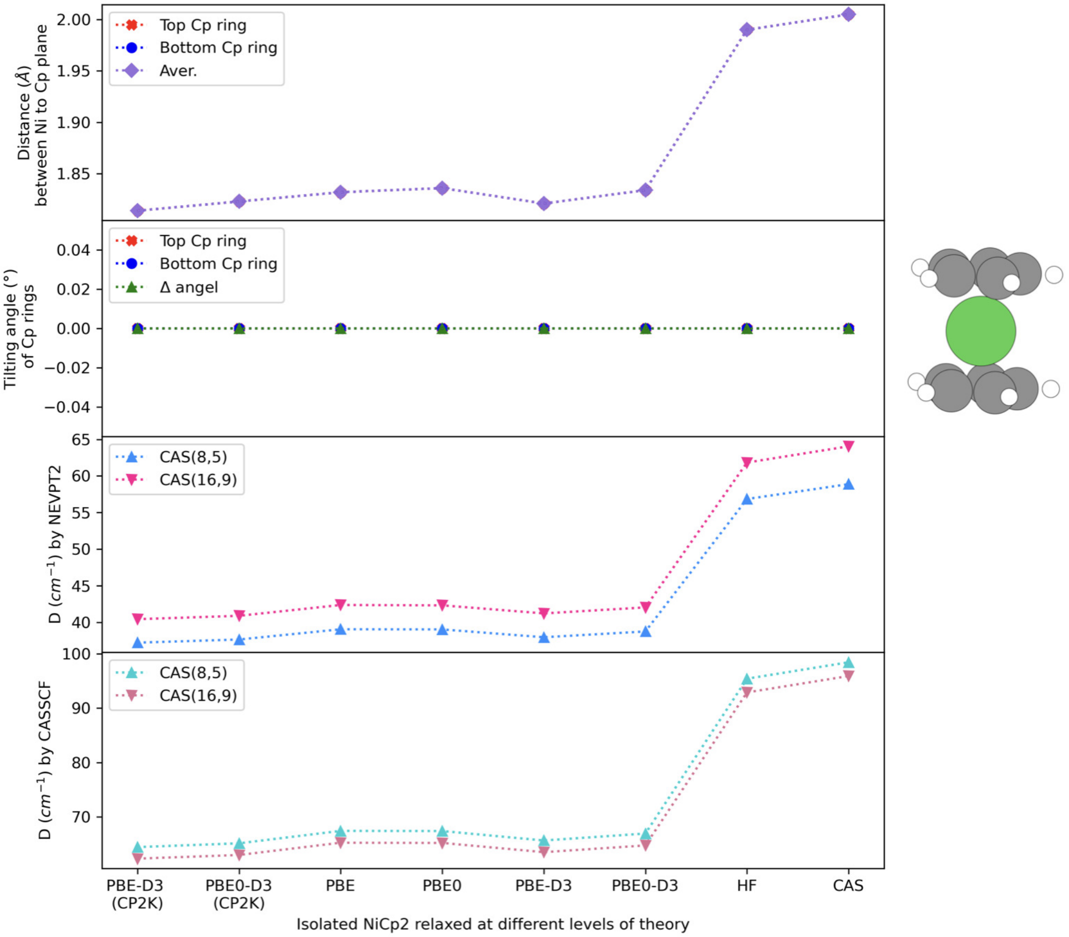

Figure S13: Comparison among the optimized $NiCp_2$ at different levels of theory, regarding the distances between Ni to Cp plane, the tilting angles, and D parameter (cm^{-1}) calculated by NEVPT2 and CASSCF.

various $NiCp_2$ conformations was computed using ab initio wavefunction theory at CASSCF and NEVPT2 levels, with quasidegenerate perturbation theory (QDPT) treatment of the spin-orbital interaction [7], using the def2-TZVP basis set. Significant distortions in the geometry result in a substantial increase in the D parameter for $NiCp_2$, accompanied by a reduction in molecular spin. In contrast, tilted geometrical changes have minimal impact on the D parameter, since the Cp rings remain approximately parallel. In this case, the D parameter is predominantly influenced by the distance between the Ni atom and the Cp plane.

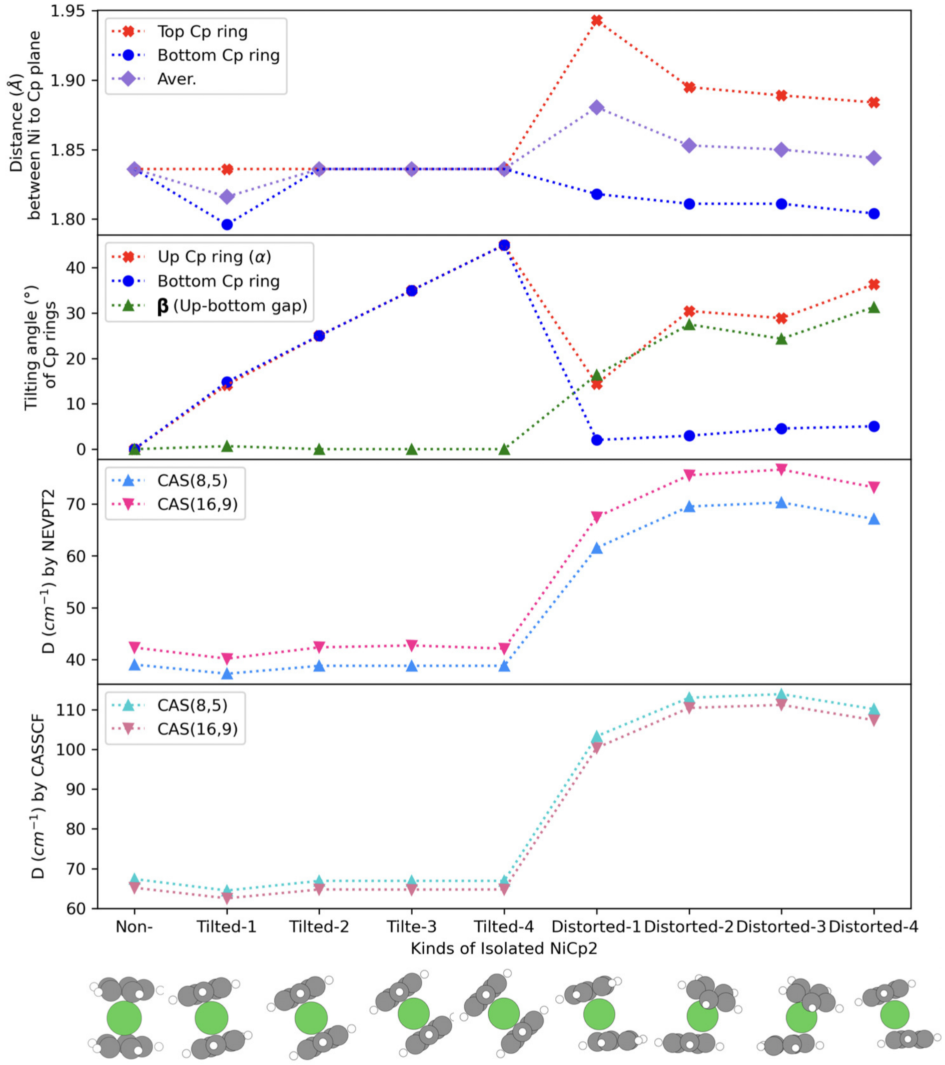

Figure S14: Comparison of various $NiCp_2$ geometries, including the Ni-to-Cp plane distances, tilting angles, D parameters (in cm^{-1}) calculated using NEVPT2, and D parameters (in cm^{-1}) calculated using CASSCF. The reference geometry (first column) represents $NiCp_2$ in the gas phase, optimized at the PBE0 level of theory in ORCA. The tilted-1 is extracted from the optimized $NiCp_2$@Fe@Cu(111) large model. The tilted-2,3,4 are constructed with α = 25, 35 and 45 ° and $\beta = 0°$. Distorted geometries are extracted from the optimized models: distorted-1 from $NiCp_2$@Cr@Cu(100) small model, distorted-2 from $NiCp_2$@V@Cu(111) small model, distorted-3 from $NiCp_2$@Mn@Cu(111) small model, and distorted-4 from $NiCp_2$@Fe@Cu(111) small model. The corresponding $NiCp_2$ structures are shown beneath each plot, and their β values are plotted by green line in the second panel.

S.4 $NiCp_2$@TM@Cu complexes

S.4.1 The structural information for $NiCp_2$@TM@Cu based models

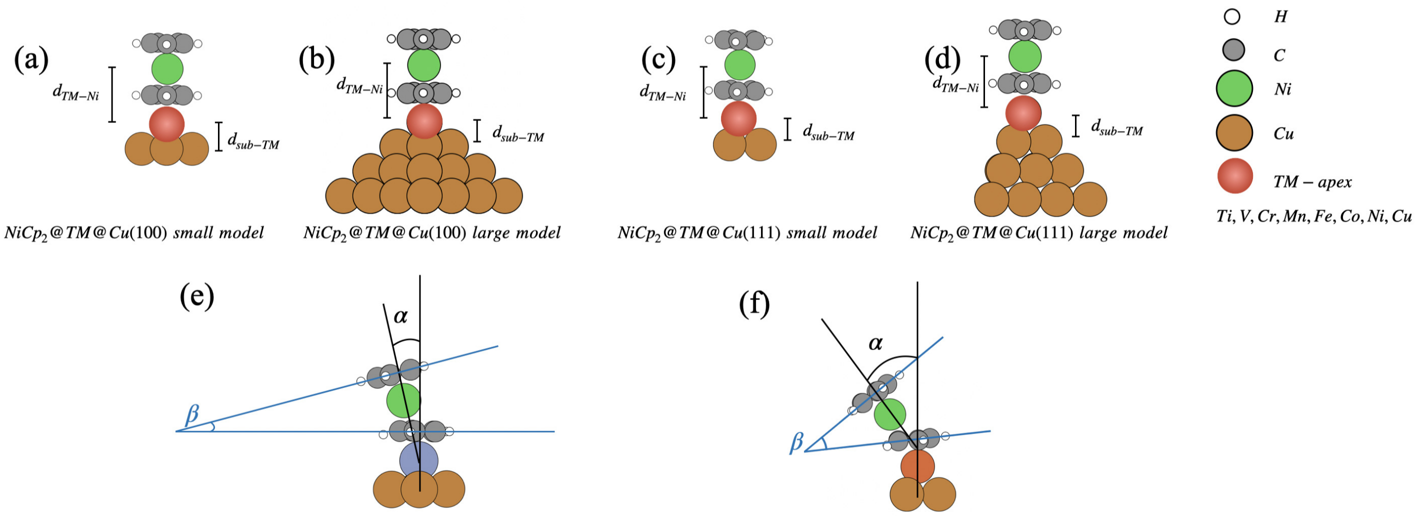

Figure S15: Diagrams for (a)$NiCp_2$@TM@Cu(100) tip models, (b)$NiCp_2$@TM@Cu(100) pyramid models, (c)$NiCp_2$@TM@Cu(111) tip models, and (d)$NiCp_2$@TM@Cu(111) pyramid models. (e) and (f) present the definition of α and β.

Table S2: Structural details for $NiCp_2$@TM@Cu(100) and $NiCp_2$@TM@Cu(111) tip and pyramid models, optimized at PBE0-D3 in ORCA and CP2K, at the spin ground state. The interaction energy, $E_{\rm int}$, is calculated as $E_{\rm int} = E_{\rm tot} - E_{\rm sub} - E_{\rm mol}$, where $E_{\rm sub}$ and $E_{\rm mol}$ represent the respective subsystems at the same coordinates as those in the optimized full junction structure.

Apex Element (TM)	Ti	V	Cr	Mn	Fe	Co	Ni	Cu
$NiCp_2$@TM@Cu(100) tip models (optimized at PBE0-D3, ORCA)								
$d_{\rm TM-Ni}$(Å)	3.80	3.77	3.97	4.04	3.88	3.82	3.66	4.01
$d_{\rm sub-TM}$(Å)	1.81	1.78	1.80	1.72	1.64	1.61	1.58	1.65
α (°)	0.43	1.25	13.03	1.82	4.92	4.04	1.15	2.29
β (°)	0.05	0.26	15.34	0.16	0.57	0.45	0.07	0.11
$E_{\rm int}$(eV)	-1.57	-1.92	-2.15	-1.01	-1.39	-1.24	-1.27	-0.61
$NiCp_2$@TM@Cu(100) pyramid models (optimized at PBE0-D3, CP2K)								
$d_{\rm TM-Ni}$(Å)	3.92	3.80	3.98	4.09	3.88	3.67	3.63	3.79
$d_{\rm sub-TM}$(Å)	1.88	1.90	1.93	1.85	1.75	1.71	1.66	1.63
α (°)	0.63	0.54	0.16	1.65	4.85	0.75	0.66	0.12
β (°)	0.08	0.29	0.70	0.01	0.13	1.81	0.24	0.05
$E_{\rm int}$(eV)	-1.75	-1.96	-1.30	-1.09	-1.28	-1.12	-1.33	-1.02
$NiCp_2$@TM@Cu(111) tip models (optimized at PBE0-D3, ORCA)								
$d_{\rm TM-Ni}$(Å)	3.79	4.06	3.95	4.17	4.07	3.56	3.79	3.86
$d_{\rm sub-TM}$(Å)	2.18	2.13	2.08	2.04	1.96	1.92	1.82	1.88
α (°)	1.45	30.24	1.76	28.85	36.37	4.11	9.35	3.50
β (°)	0.23	27.71	0.20	24.31	31.32	0.87	12.92	0.29
$E_{\rm int}$(eV)	-1.72	-2.97	-1.12	-1.96	-2.36	-1.58	-2.19	-0.86
$NiCp_2$@TM@Cu(111) pyramid models (optimized at PBE0-D3, CP2K)								
$d_{\rm TM-Ni}$(Å)	3.80	4.02	4.07	4.20	3.83	3.79	3.74	3.87
$d_{\rm sub-TM}$(Å)	2.36	2.32	2.23	2.22	2.05	2.06	1.99	1.95
α (°)	1.37	32.02	5.24	30.19	14.16	0.53	4.49	2.88
β (°)	0.23	29.32	0.59	27.37	0.66	0.45	0.41	0.48
$E_{\rm int}$(eV)	-1.72	-2.64	-1.15	-2.18	-1.35	-1.21	-1.11	-0.96

S.4.2 The sum of magnetic and electronic properties for $NiCp_2$@TM@Cu based models

Table S3: The Mulliken spin moment and charge for $NiCp_2$@TM@Cu(100) tip and pyramid models, calculated at CASSCF, PBE0-D3 in ORCA and PBE0-D3 in CP2K, at the spin ground state. The positive value in the Mulliken charge means donating electrons and the negative value means accepting electrons. The Cu_s in Cu(100) models means the sum of four Cu atoms which are arranged as a square, whereas in Cu(100) pyramid models, it means the sum of all the Cu atoms as substrate.

Apex Element (TM)	Ti	V	Cr[1]	Mn	Fe	Co	Ni	Cu
$NiCp_2$@TM@Cu(100) tip models (CASSCF, ORCA)								
Ground State S	2	5/2	2	3/2	1	1/2	0	1/2
Exchange Coupling	Ferro-	Ferro-	Ferro-	Anti-	Anti-	Anti-	Anti-	Anti-
E_{ex} (meV)	-462	-51	-23	107	131	113	211	44
Mulliken Spin moment of $Cu_s(\mu_B)$	-0.02	-0.19	-0.46	0.05	0.05	0.03	-	-0.30
Mulliken Spin moment of TM-apex(μ_B)	1.97	3.13	3.49	4.11	2.90	1.60	-	-0.02
Mulliken Spin moment of Ni(μ_B)	1.83	1.82	0.95	-1.08	-0.90	-0.60	-	1.21
Mulliken Spin moment of $NiCp_2(\mu_B)$	2.05	2.06	0.97	-1.16	-0.95	-0.63	-	1.32
Mulliken Charge on Cu_s δ(e)	-1.40	-1.31	-0.32	-0.67	-0.55	-0.49	-0.30	-0.31
Mulliken Charge on TM-apex δ(e)	0.88	0.80	0.66	0.52	0.31	0.20	0.05	0.16
Mulliken Charge on Ni δ(e)	0.58	0.61	0.56	0.64	0.64	0.66	0.71	0.75
Mulliken Charge on $NiCp_2$ δ(e)	0.53	0.51	-0.34	0.15	0.24	0.29	0.24	0.15
$NiCp_2$@TM@Cu(100) tip models (PBE0-D3, ORCA)								
Ground state S	2	5/2	2	3/2	1	1/2	0	1/2
Exchange Coupling	Ferro-	Ferro-	Ferro-	Anti-	Anti-	Anti-	Anti-	Anti-
E_{ex} (meV)	-122	-155	-55	370	478	501	585	91
Mulliken Spin moment of $Cu_s(\mu_B)$	-0.24	-0.43	-1.08	0.05	0.28	0.05	0.94	-0.84
Mulliken Spin moment of TM(μ_B)	2.04	3.42	4.30	4.77	3.44	2.23	0.70	-0.03
Mulliken Spin moment of Ni(μ_B)	1.26	1.24	0.86	-1.14	-1.16	-1.10	-1.09	1.17
Mulliken Spin moment of $NiCp_2(\mu_B)$	2.20	2.00	0.78	-1.82	-1.73	-1.68	-1.64	1.87
Mulliken Charge on Cu_s δ(e)	-1.54	-1.66	-0.93	-0.61	-0.49	-0.38	-0.31	-0.27
Mulliken Charge on TM δ(e)	1.20	1.12	1.03	0.49	0.30	0.17	0.07	0.07
Mulliken Charge on Ni δ(e)	0.24	0.25	0.25	0.26	0.27	0.28	0.31	0.37
Mulliken Charge on $NiCp_2$ δ(e)	0.34	0.54	-0.09	0.12	0.19	0.22	0.24	0.20
$NiCp_2$@TM@Cu(100) pyramid models (PBE0-D3, CP2K)								
Ground state S	3/2	3	1/2	1	1/2	1	1/2	0
Exchange Coupling	Ferro-	Ferro-	Anti-	Anti-	Anti-	Anti-	Anti-	Anti-
E_{ex} (meV)	-68	-515	63	135	217	223	75	60
Mulliken Spin moment of $Cu_s(\mu_B)$	-1.15	0.30	-1.77	-0.88	-0.55	1.88	-0.12	-1.88
Mulliken Spin moment of TM(μ_B)	2.15	3.67	4.65	4.76	3.38	-1.78	-0.67	-0.23
Mulliken Spin moment of Ni(μ_B)	1.21	1.21	-1.16	-1.16	-1.16	1.17	1.12	1.14
Mulliken Spin moment of $NiCp_2(\mu_B)$	2.00	2.03	-1.88	-1.88	-1.83	1.91	1.80	1.85
Mulliken Charge on Cu_s δ(e)	-0.50	-0.71	-0.60	-0.68	-0.62	-0.50	-0.20	-0.28
Mulliken Charge on TM δ(e)	0.11	0.39	0.34	0.43	0.23	0.17	-0.20	0.06
Mulliken Charge on Ni δ(e)	0.14	0.15	0.14	0.14	0.14	0.16	0.14	0.12
Mulliken Charge on $NiCp_2$ δ(e)	0.39	0.32	0.26	0.25	0.39	0.34	0.39	0.22

[1] A size-dependent discrepancy arises in this case: while the distorted $NiCp_2$ configuration is stabilized in the small tip model, the non-distorted geometry becomes energetically preferred in the larger pyramid model.

Table S4: The Mulliken spin moment and charge for $NiCp_2$@TM@Cu(111) tip and pyramid models, calculated at CASSCF, PBE0-D3 in ORCA and PBE0-D3 in CP2K, at the spin ground state. The positive value in the Mulliken charge means donating electrons and the negative value means accepting electrons. The Cu_s in Cu(111) models means the sum of three Cu atoms which are arranged as a triangle, whereas in Cu(111) pyramid models, it means the sum of all the Cu atoms as substrate.

Apex Element (TM)	Ti	V[1]	Cr	Mn[1]	Fe[1,2]	Co	Ni [2]	Cu
$NiCp_2$@TM@Cu(111) tip models (CASSCF, ORCA)								
Ground State S	7/2	3	1/2	1	1/2	1	1/2	0
Exchange Coupling	Ferro-	Ferro-	Anti-	Anti-	Anti-	Anti-	Anti-	Anti-
E_{ex} (meV)	-52	-4	7	1	6	402	1129	58
Mulliken Spin moment of Cu_s(μ_B)	1.65	1.37	-0.59	-0.68	-0.40	0.00	0.01	-
Mulliken Spin moment of TM-apex(μ_B)	3.08	3.53	2.23	3.18	1.73	0.01	0.00	-
Mulliken Spin moment of Ni(μ_B)	1.84	0.96	-0.61	-0.48	-0.32	1.82	0.96	-
Mulliken Spin moment of $NiCp_2$(μ_B)	2.27	1.10	-0.64	-0.50	-0.33	1.99	0.99	-
Mulliken Charge on Cu_s δ(e)	-0.36	0.05	-0.74	0.03	0.08	-0.28	0.23	-0.35
Mulliken Charge on TM-apex δ(e)	0.14	0.39	0.57	0.44	0.35	0.01	0.14	0.10
Mulliken Charge on Ni δ(e)	0.66	0.58	0.64	0.61	0.61	0.73	0.63	0.74
Mulliken Charge on $NiCp_2$ δ(e)	0.21	-0.44	0.17	-0.47	-0.43	0.27	-0.37	0.25
$NiCp_2$@TM@Cu(111) tip models (PBE0-D3, ORCA)								
Ground state S	7/2	3	1/2	2	3/2	1	1/2	0
Exchange Coupling	Ferro-	Ferro-	Anti-	Ferro-	Ferro-	Anti-	Anti-	Anti-
E_{ex} (meV)	-98	-7	117	-4	-15	527	725	242
Mulliken Spin moment of Cu_s (μ_B)	1.66	1.40	-1.87	-1.53	-1.36	1.54	0.73	1.70
Mulliken Spin moment of TM (μ_B)	3.05	3.67	4.74	4.53	3.28	-1.49	-0.57	0.08
Mulliken Spin moment of Ni (μ_B)	1.25	0.93	-1.20	0.94	0.95	1.22	0.85	-1.13
Mulliken Spin moment of $NiCp_2$ (μ_B)	2.29	0.93	-1.87	1.00	1.08	1.95	0.84	-1.78
Mulliken Charge on Cu_s δ(e)	-0.52	-0.16	-0.95	-0.13	-0.06	-0.26	0.11	-0.14
Mulliken Charge on TM δ(e)	0.34	0.35	0.80	0.38	0.27	-0.10	0.03	-0.19
Mulliken Charge on Ni δ(e)	0.30	0.31	0.25	0.31	0.31	0.36	0.32	0.37
Mulliken Charge on $NiCp_2$ δ(e)	0.18	-0.19	0.16	-0.25	-0.21	0.36	-0.15	0.33
$NiCp_2$@TM@Cu(111) pyramid models (PBE0-D3, CP2K)								
Ground state S	5/2	2	3/2	3	1/2	0	1/2	1
Exchange Coupling	Ferro-	Ferro-	Anti-	Ferro-	Anti[2]	Anti-	Anti-	Para-
E_{ex} (meV)	-154	-14	118	-7	143	130	91	-
Mulliken Spin moment of Cu_s (μ_B)	0.01	-0.18	-0.02	-0.05	-0.18	-0.05	0.03	0.01
Mulliken Spin moment of TM(μ_B)	2.70	3.23	4.90	4.97	3.12	1.97	-0.92	0.06
Mulliken Spin moment of Ni(μ_B)	1.22	0.94	-1.15	0.94	-1.17	-1.18	1.17	1.18
Mulliken Spin moment of $NiCp_2$(μ_B)	2.28	0.95	-1.88	1.08	-1.94	-1.92	1.89	1.93
Mulliken Charge on Cu_s δ(e)	-0.43	-0.34	-0.56	-0.25	-0.51	-0.54	-0.30	-0.27
Mulliken Charge on TM δ(e)	0.24	0.53	0.41	0.46	0.25	0.27	-0.01	0.11
Mulliken Charge on Ni δ(e)	0.17	0.21	0.16	0.21	0.16	0.17	0.16	0.14
Mulliken Charge on $NiCp_2$ δ(e)	0.19	-0.19	0.15	-0.22	0.26	0.27	0.31	0.16

[1] There are three distorted $NiCp_2$ configuration. This results in discrepancies between CAS and DFT predictions regarding the ground spin state for Mn and Fe, even when evaluated on the same geometry caused by the limitation in DFT method. A detailed comparison of these near-degenerate states is provided in Table S6 (V), Table S7 (Mn), and Table S8 (Fe).

[2] A size-dependent discrepancy arises in this case: in the small tip model, $NiCp_2$ adopts a distorted geometry with spin reduction, whereas in the larger pyramid model, it reverts to a non-distorted configuration (titling) with spin retaining. The size effect leads to inconsistencies between the two model predictions.

Table S5: The J values for $NiCp_2$@TM@Cu-based small and large pyramid models, calculated at CASSCF, PBE0-D3 in ORCA and PBE0-D3 in CP2K. The positive J indicates the anti-ferromagnetic coupling between and Ni of $NiCp_2$, whereas the negative J means ferromagnetic coupling. The reason for some cases have no J values is either TM or Ni does no spin moment, $s_i = 0$.

Apex Element (TM)	Ti	V	Cr	Mn	Fe	Co	Ni	Cu
J values for $NiCp_2$@TM@Cu(100) models								
(small)CASSCF, ORCA (cm^{-1})	-116.56	-8.50	-5.80	27.04	43.97	57.10	–	–
(small)PBE0-D3, ORCA (cm^{-1})	-61.72	-52.18	-13.82	74.58	160.58	252.52	589.86	–
(large)PBE0-D3, CP2K (cm^{-1})	-34.26	-129.83	12.70	27.27	73.01	112.54	75.39	–
J values for$NiCp_2$@TM@Cu(111) models								
(small)CASSCF, ORCA (cm^{-1})	-8.71	-1.29	3.38	0.48	3.22	–	–	–
(small)PBE0-D3, ORCA (cm^{-1})	-32.94	-1.67	23.54	-0.81	-4.97	265.48	730.63	–
(large)PBE0-D3, CP2K (cm^{-1})	-51.76	-4.70	23.79	-1.41	48.06	65.47	91.72	–

S.4.3 The orbital energy levels for NiCp$_2$@TM@Cu(100) tip models at PBE0-D3 level of theory

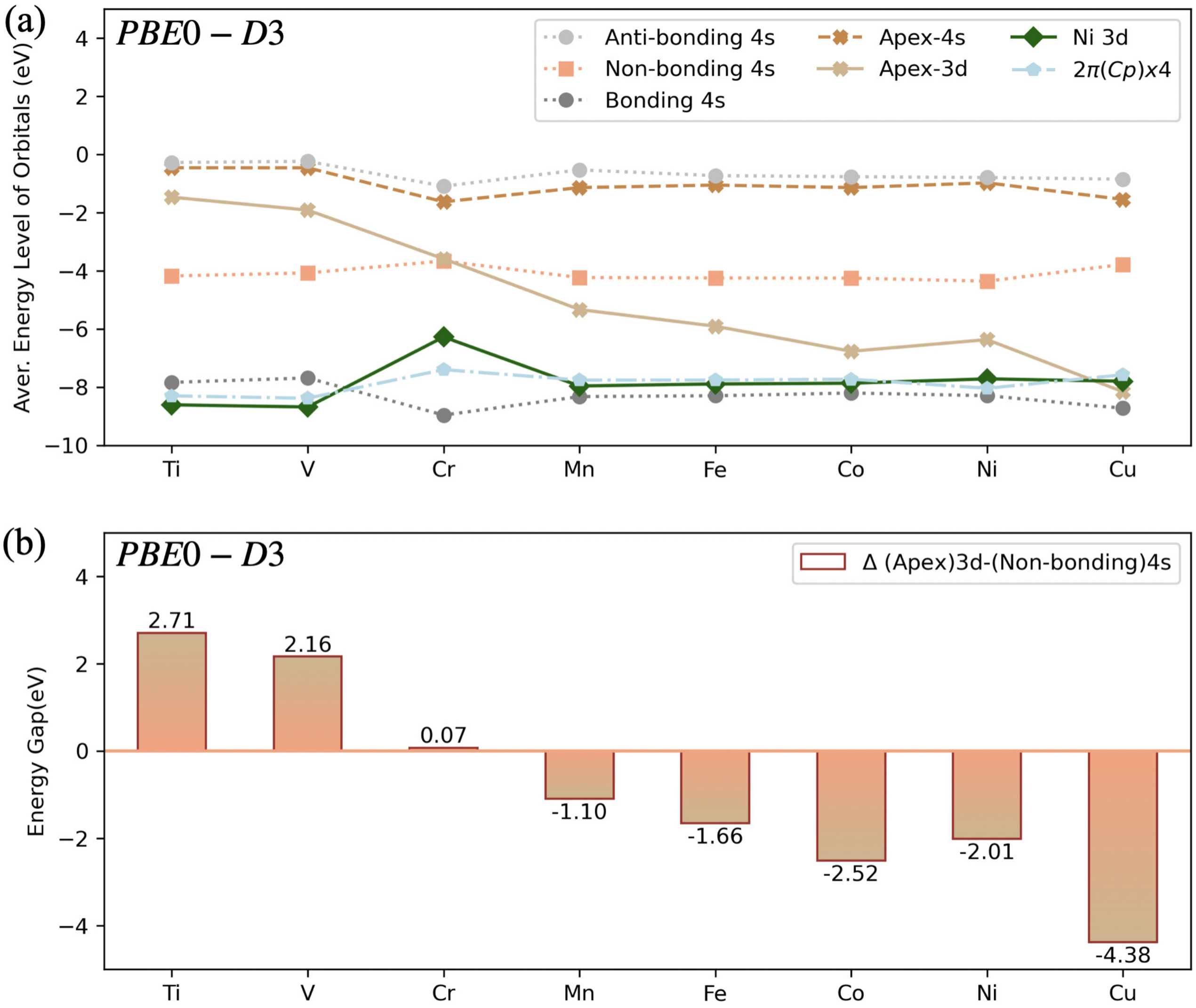

Figure S16: The energy levels (at PBE0-D3 level of theory) of 4s and 3d orbitals for NiCp$_2$@TM@Cu(100) tip models (TM: Ti, V, Cr, Mn, Fe, Co, Ni, Cu).

S.4.4 The orbital energy levels for NiCp$_2$@TM@Cu(111) tip models at PBE0-D3 level of theory

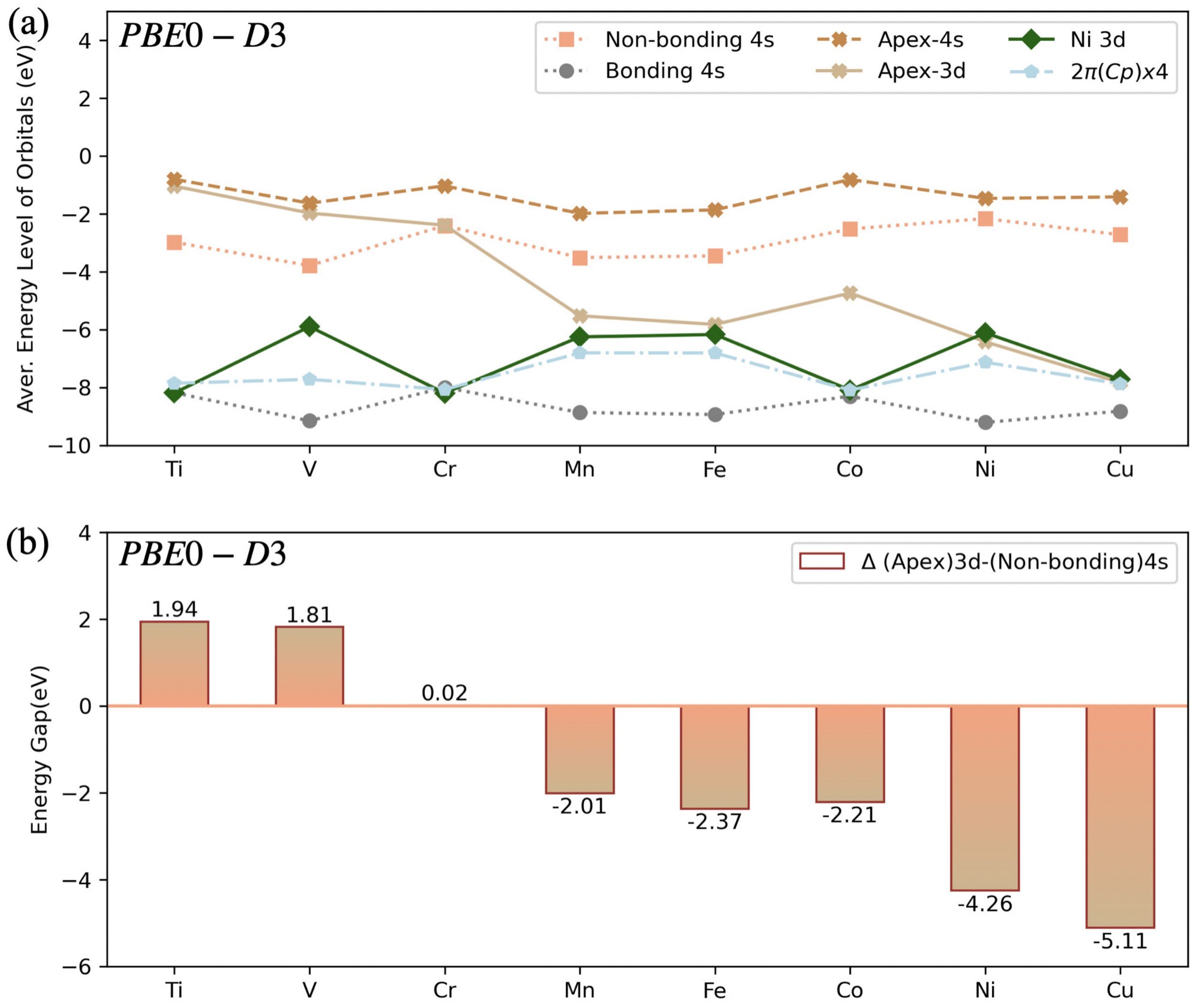

Figure S17: The energy levels (at PBE0-D3 level of theory) of 4s and 3d orbitals for NiCp$_2$@TM@Cu(111) tip models (TM: Ti, V, Cr, Mn, Fe, Co, Ni, Cu).

S.4.5 CASSCF MOs for NiCp$_2$@TM@Cu tip models

The atomic structures of the NiCp$_2$@TM@Cu(100) tip models are illustrated in the first row of Figure 3 in the main part. The electronic structures at CAS level are described by the occupancy in the left panel of Figure 4 corresponding to CAS MOs illustrated from Figure S21 to S34, separately, and the energy levels of orbitals at PBE0-D3 level are plotted in Figure S16. Table S3 summarizes the Mulliken spin moments and charge distributions at their ground state. Across all structures, there is one doubly occupied bonding orbital (shown in black) and two unoccupied 4s orbitals at higher energy levels (shown in grey). These unoccupied orbitals consist of one 4s orbital from the apex atom and an anti-bonding 4s orbital.

Two primary types of hybridization are identified within the active space. The first type involves 4s and 3d (d_{xz} and d_{yz}) orbital hybridization, observed in systems with apex atoms Ti, V, Cr (illustrated in rosy-brown lines of Figure S21, S22, S33), as well as that in previous structure without NiCp$_2$, which will minimize with the atomic number increasing. The second type features hybridization between the d_{xz} and d_{yz} orbitals of the Ni atom in NiCp$_2$ and those of the apex atoms (Mn, Fe, Co, and Ni), as shown in khaki lines of Figure S34, S23, S24, S25, forming antiferromagnetic coupling between NiCp$_2$ and the apex elements.

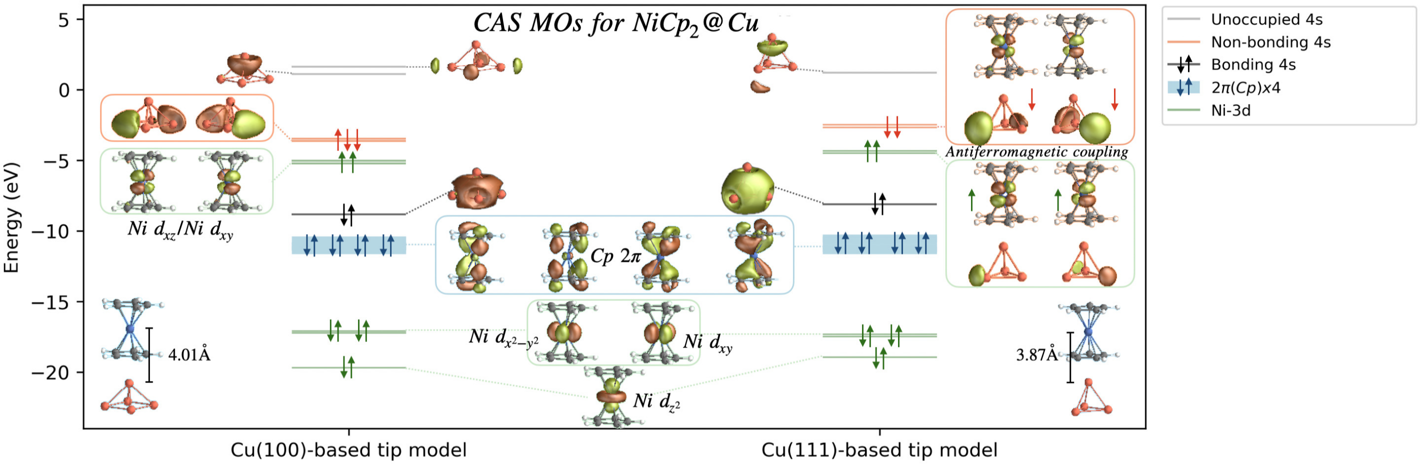

Figure S18: CASSCF MOs diagrams for NiCp$_2$-@u(100) small model on the left side, and NiCp$_2$@Cu(111) on the right side. Due to Cu of $3d^{10}4s^1$, the 5 doubly occupied 3d orbitals of Cu apex do not presented here.

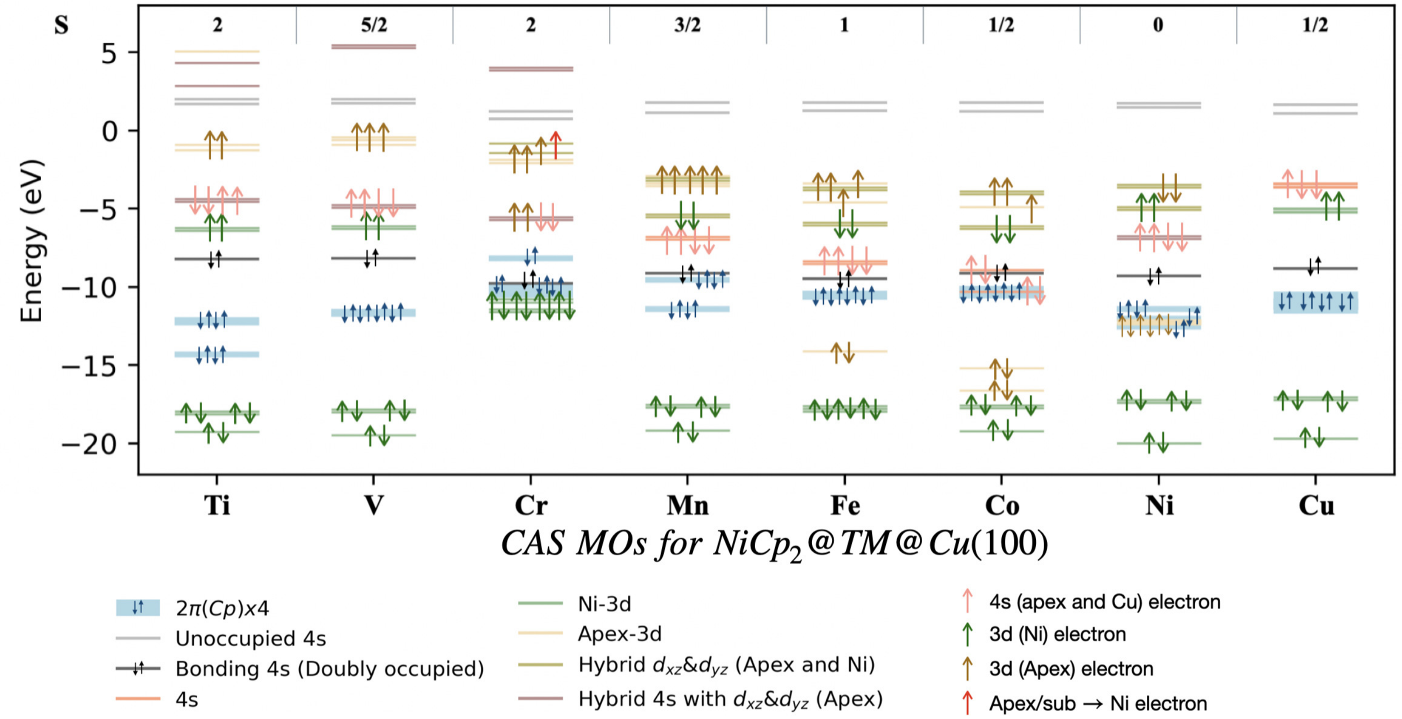

Figure S19: CASSCF MOs diagrams for $NiCp_2$@TM@Cu(100) small models, corresponding to the occupancy map in the left panel of Figure 4.

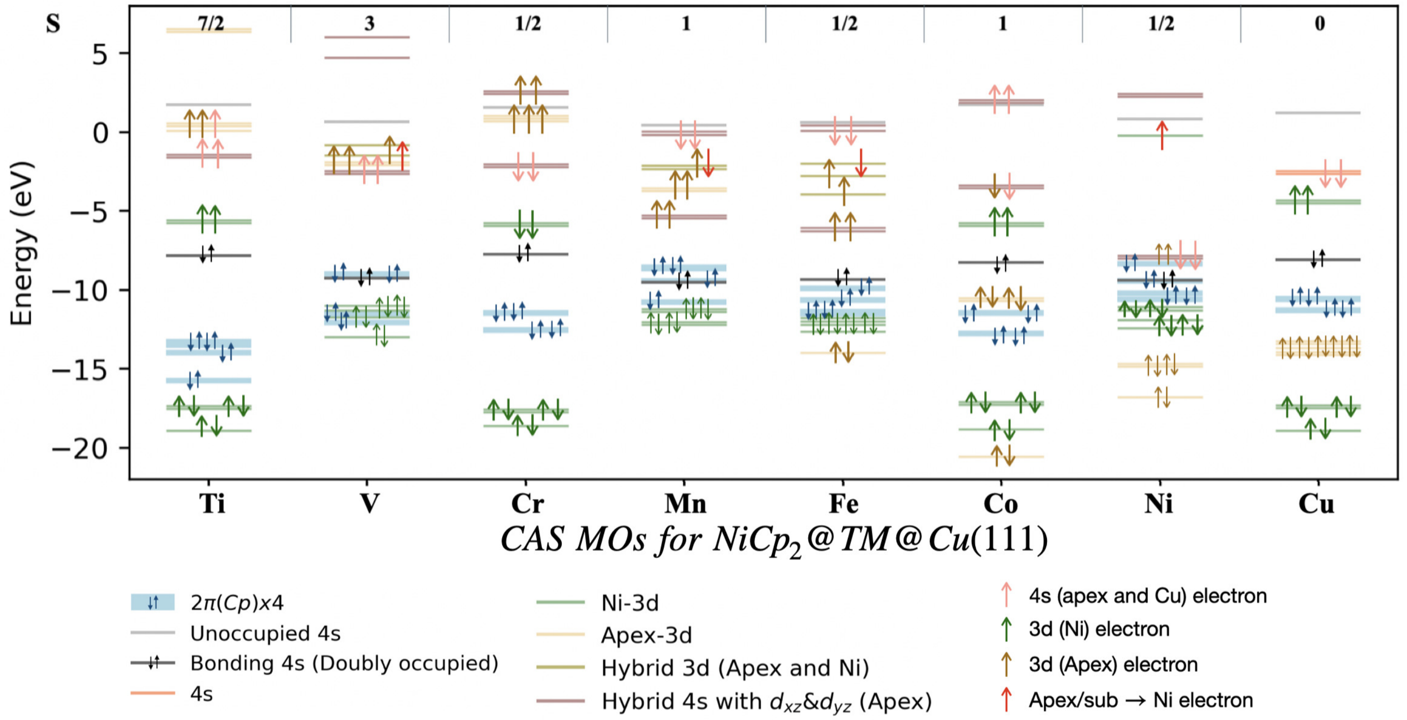

Figure S20: CASSCF MOs diagrams for $NiCp_2$@TM@Cu(111) small models, , corresponding to the occupancy map in the right panel of Figure 4.

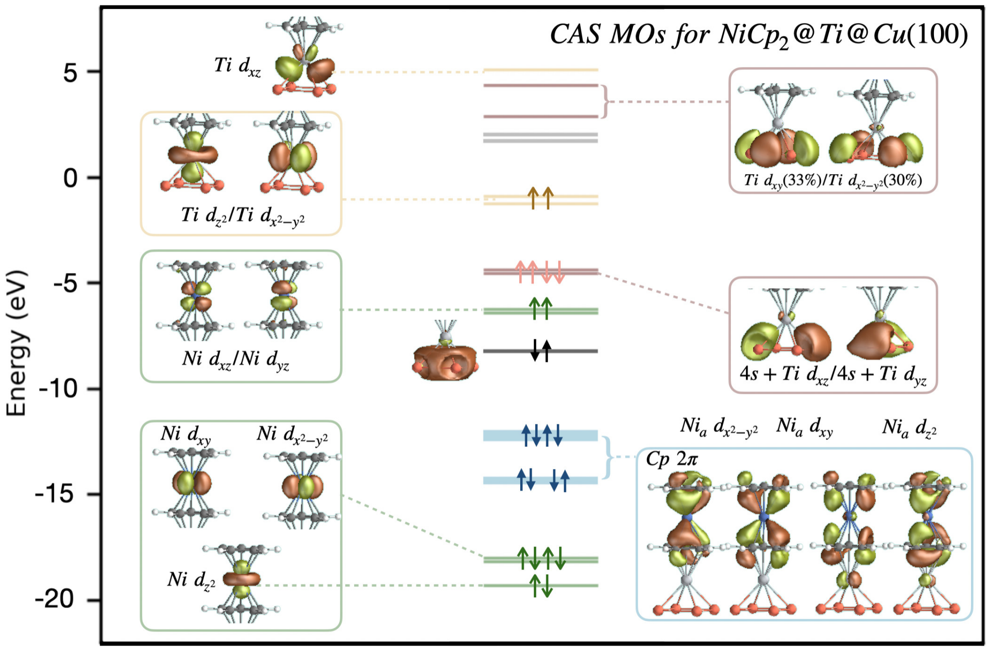

Figure S21: CASSCF MOs diagrams for $NiCp_2$@Ti@Cu(100) small model.

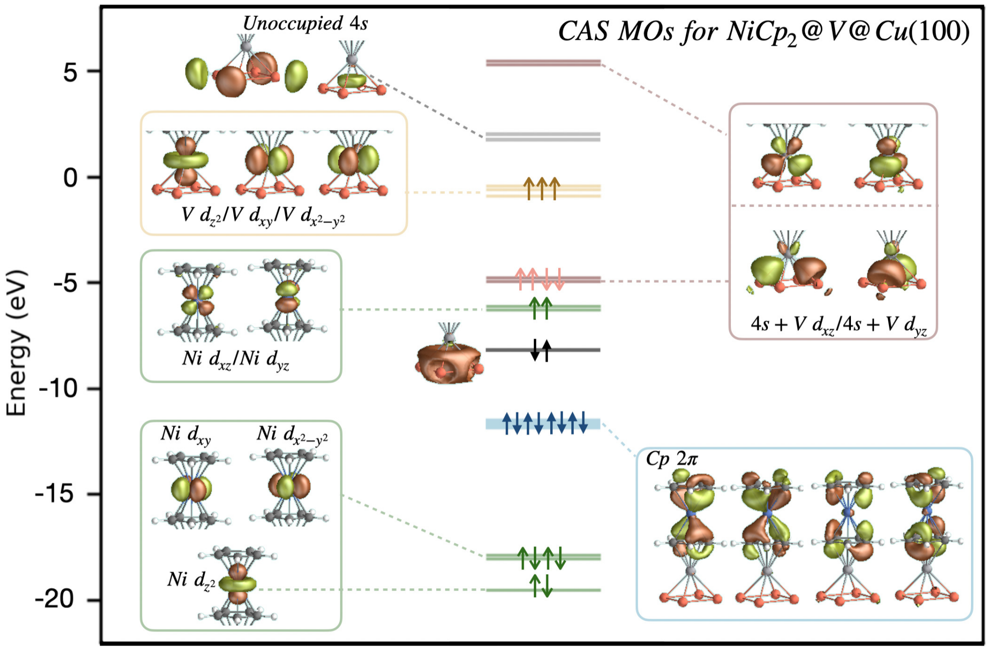

Figure S22: CASSCF MOs diagrams for $NiCp_2$-V-Cu(100) tip model.

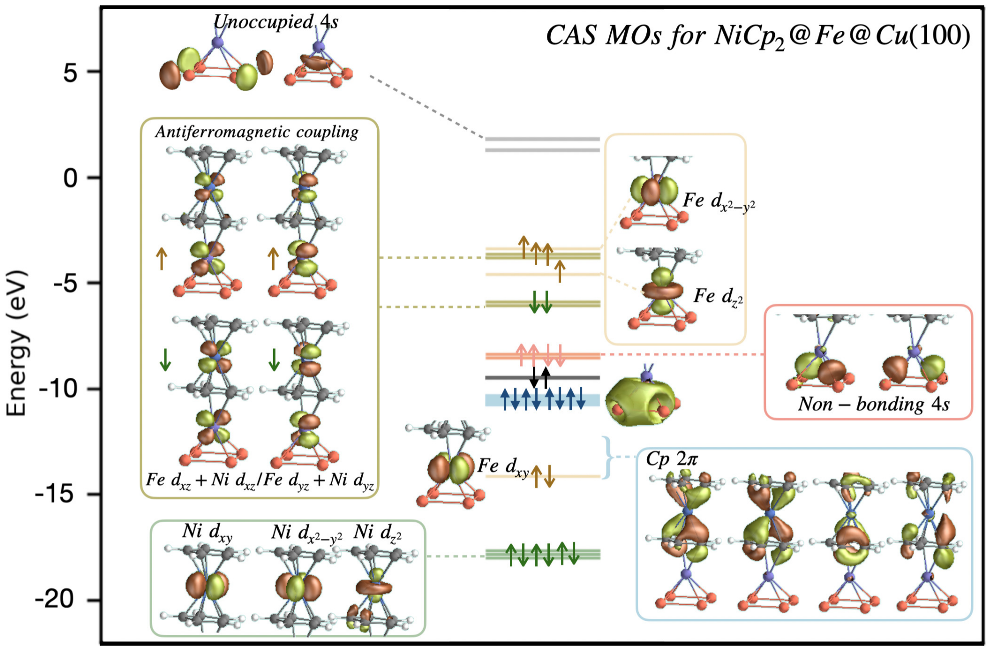

Figure S23: CASSCF MOs diagrams for $NiCp_2$@Fe@Cu(100) small model.

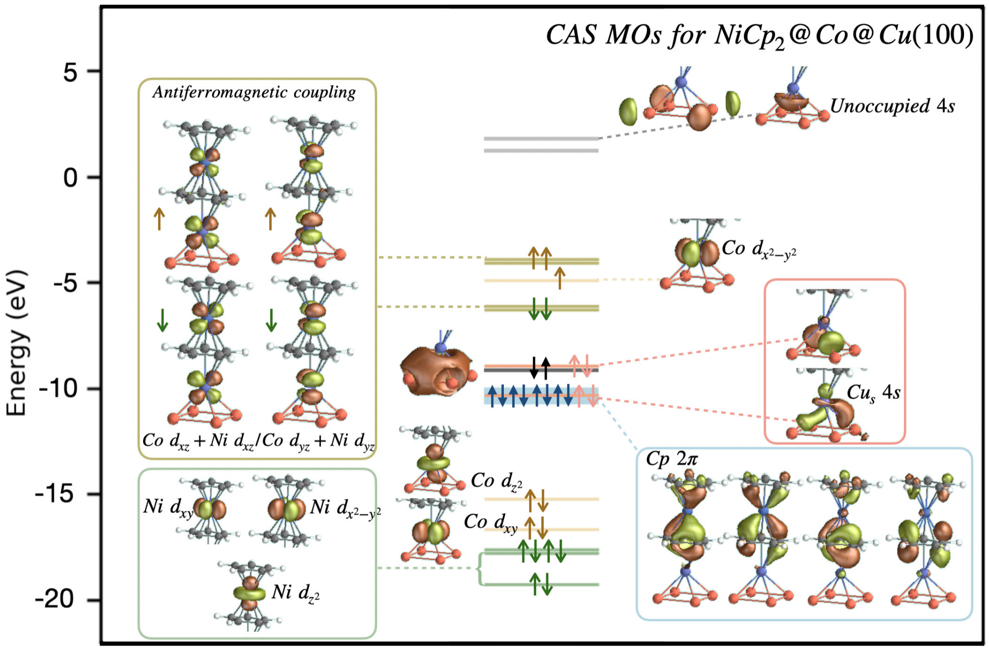

Figure S24: CASSCF MOs diagrams for $NiCp_2$@Co@Cu(100) small model.

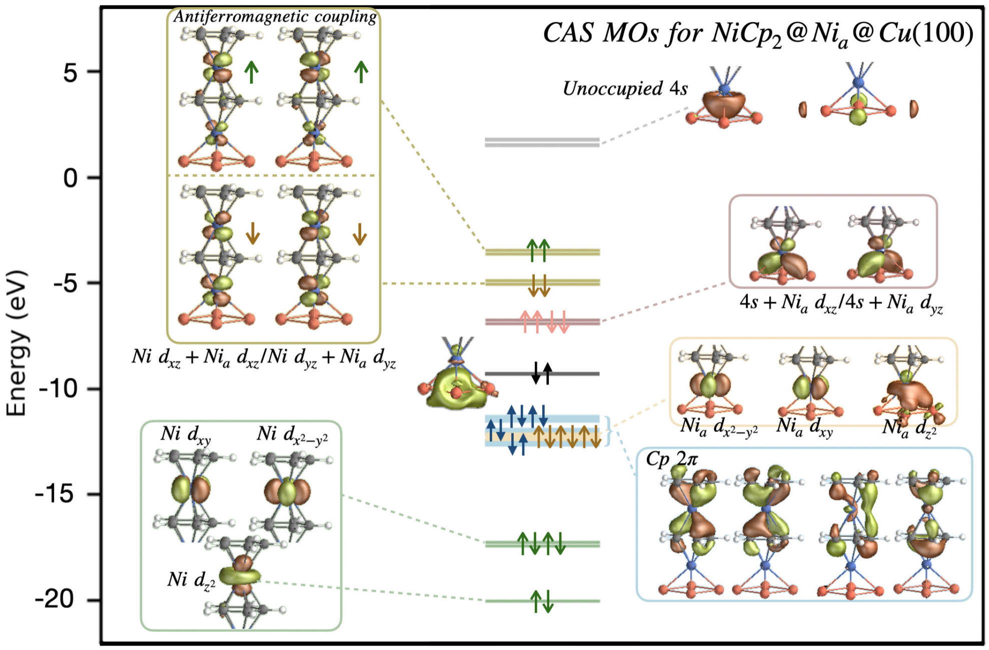

Figure S25: CASSCF MOs diagrams for NiCp$_2$@Ni@Cu(100) small model.

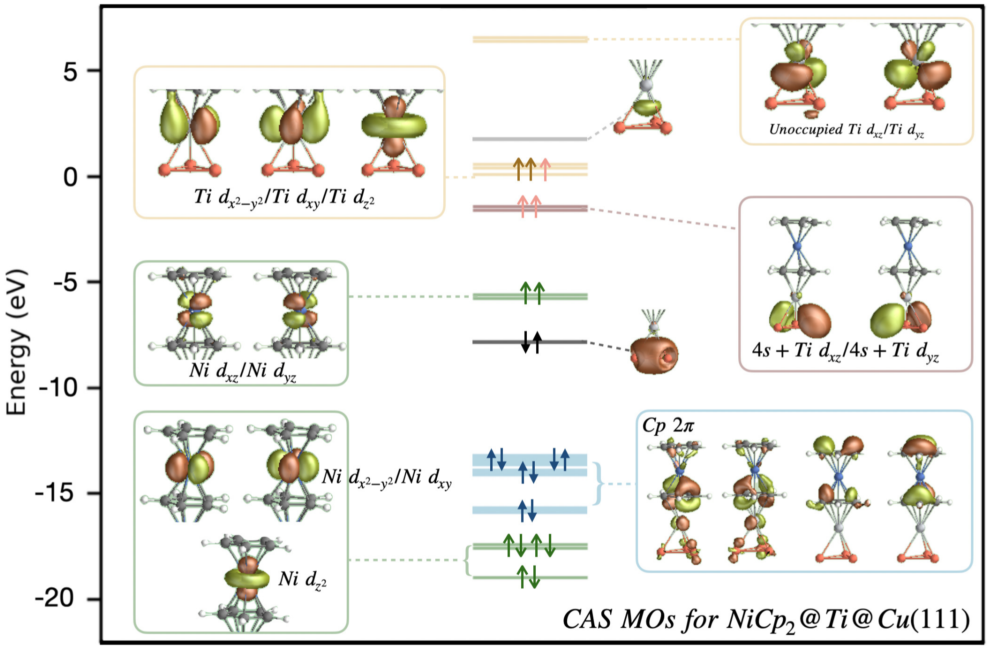

Figure S26: CASSCF MOs diagrams for NiCp$_2$@Ti@Cu(111) small model.

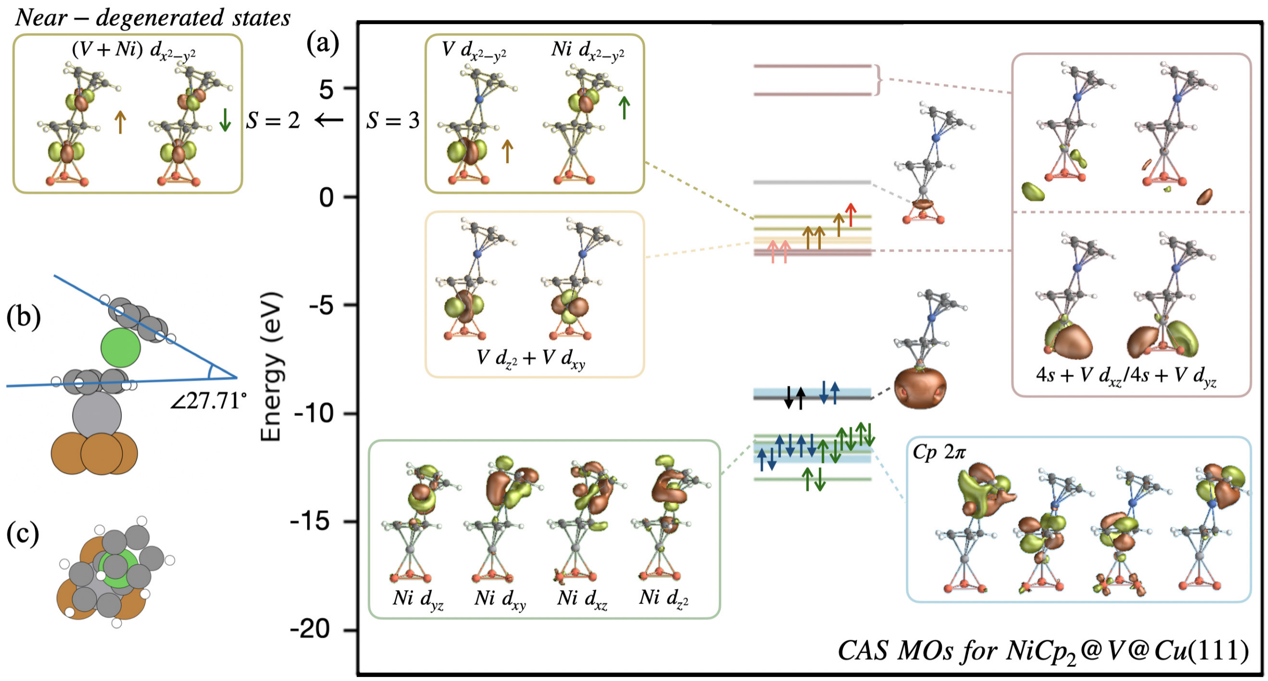

Figure S27: CASSCF MOs diagrams for $NiCp_2$@V@Cu(111) small model.

Table S6: Comparison of two degenerate spin states in the $NiCp_2$@V@Cu(111) small model, shown in Figure S27. Mulliken spin and charge populations, calculated at the CASSCF level of theory, are provided. Positive Mulliken charges δ(e) indicate charge transfer from the V-Cu substrate to $NiCp_2$, designating $NiCp_2$ as an electron acceptor.

Spin State	S = 2	S = 3
Exchange coupling	Anti-ferromagnetic	Ferromagnetic
Mulliken Spin moment of V (μ_B)	3.28	3.52
Mulliken Spin moment of Ni (μ_B)	-0.64	0.96
Mulliken Spin moment of $NiCp_2$ (μ_B)	-0.57	1.10
Mulliken Charge on $NiCp_2$ δ(e)	-0.44	-0.44
$Transition\ Energy(cm^{-1})$ by CASSCF	+33.4	0.0
$Transition\ Energy(cm^{-1})$ by NEVPT2	+12.8	0.0
$Transition\ Energy(cm^{-1})$by PBE0-D3	+48.0	0.0

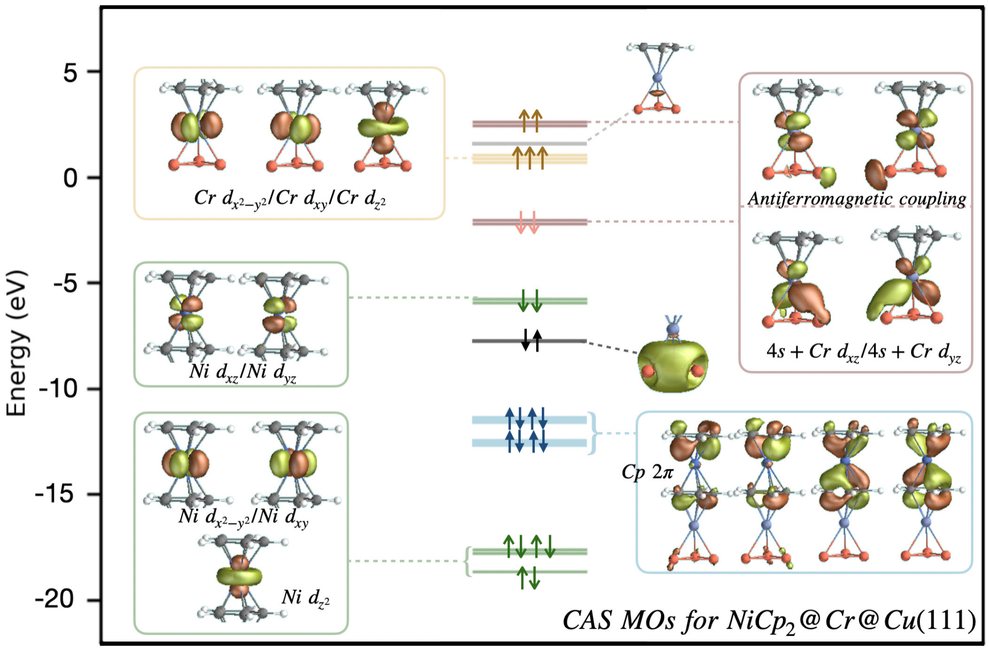

Figure S28: CASSCF MOs diagrams for $NiCp_2$@Cr@Cu(111) small model.

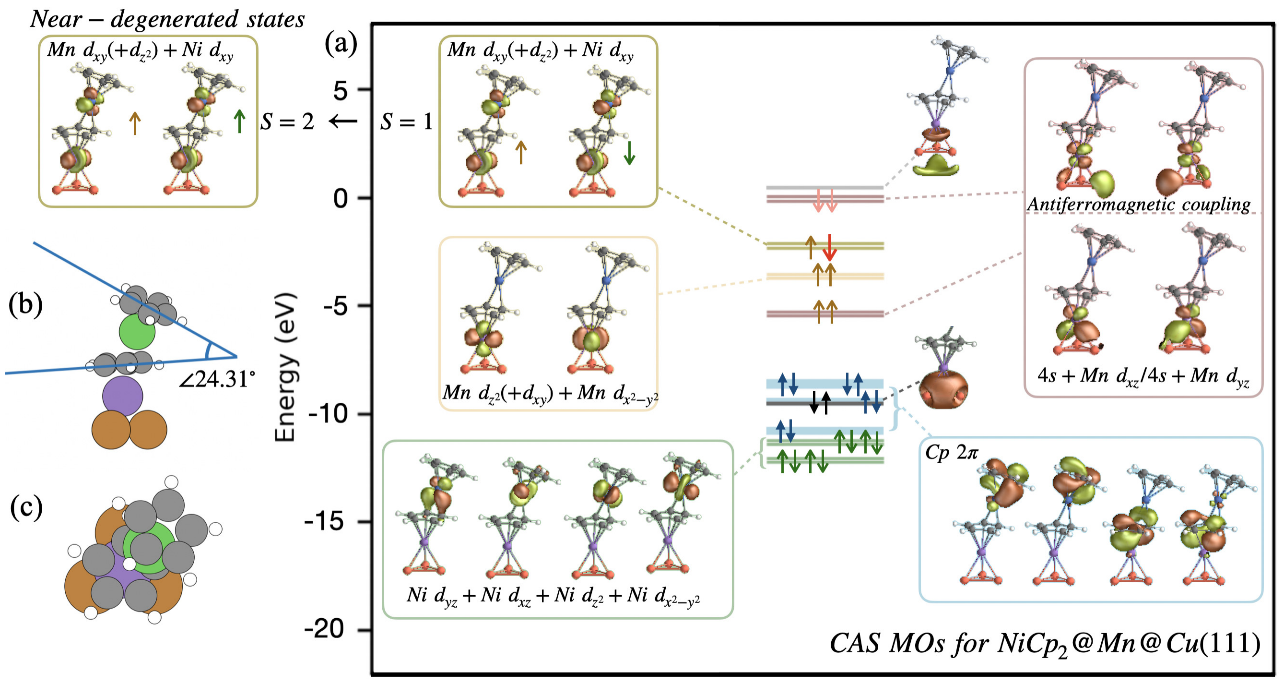

Figure S29: CASSCF MOs diagrams for NiCp$_2$@Mn@Cu(111) small model.

Table S7: The comparison between two degenerated states in NiCp$_2$@Mn@Cu(111) small model, shown in Figure S29. The Mulliken spin and charge distribution are simulated at CAS level.

Spin State	S = 1	S = 2
Exchange coupling	Anti-ferromagnetic	Ferromagnetic
Mulliken Spin moment of Mn (μ_B)	3.18	3.82
Mulliken Spin moment of Ni (μ_B)	-0.48	0.97
Mulliken Spin moment of NiCp$_2$ (μ_B)	-0.50	0.99
Mulliken Charge on NiCp$_2$ δ(e)	-0.47	-0.47
$Transition\ Energy(cm^{-1})$ by CASSCF	0.0	+11.9
$Transition\ Energy(cm^{-1})$ by NEVPT2	0.0	+27.0
$Transition\ Energy(cm^{-1})$by PBE0-D3	+35.4	0.0

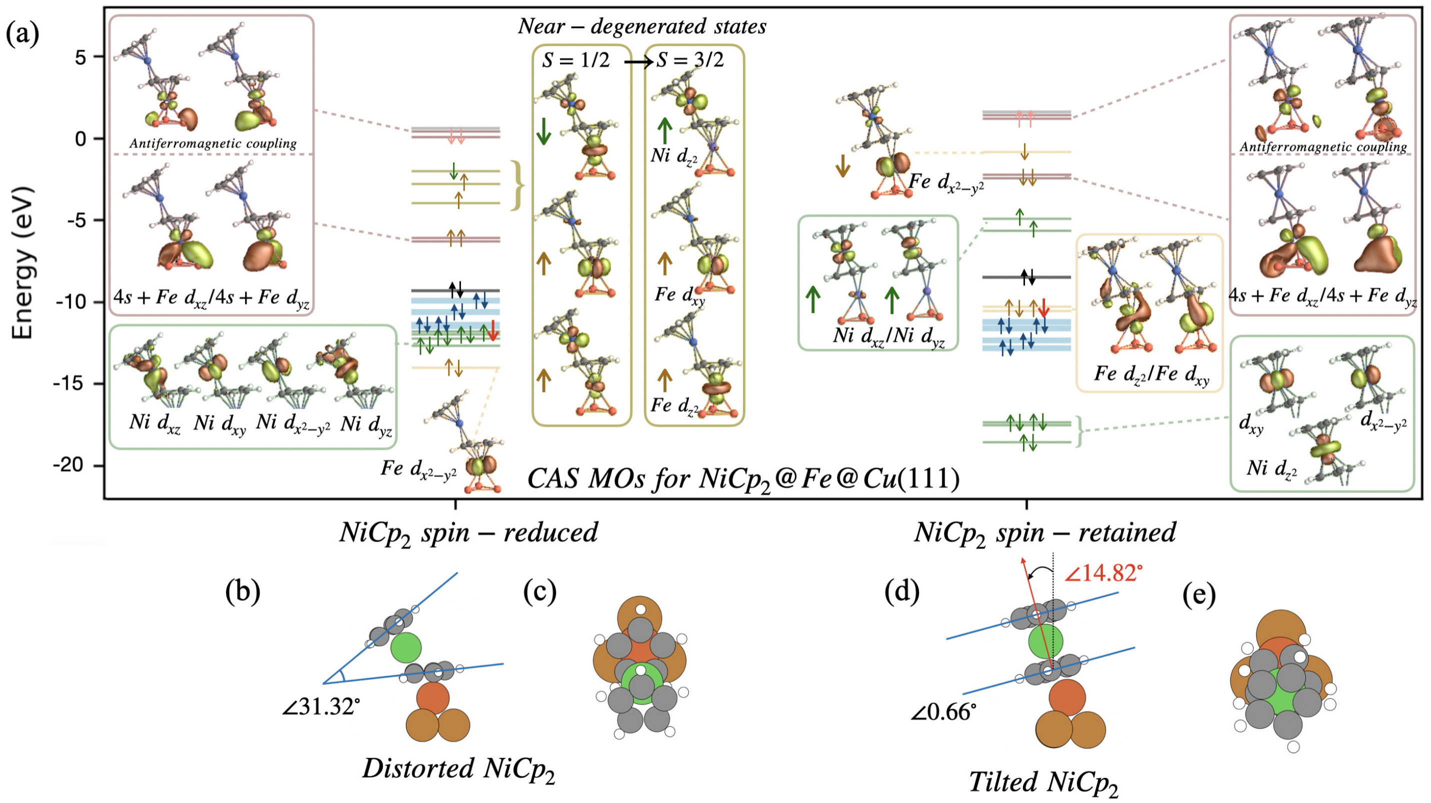

Figure S30: CASSCF MOs diagrams for $NiCp_2$@Fe@Cu(111) small models. The right structure is extracted from the corresponding optimized pyramid model. In spin-retained case, Fe adopts a $3d^7$ configuration, while Ni retains a $3d^8$ configuration between parallel Cp rings, preserving its spin state of $S = 1$.

Table S8: The comparison between two degenerated states in spin-reduced $NiCp_2$@Fe@Cu(111) small model, shown in the left part of Figure S30(a). The Mulliken spin and charge distribution are simulated at CAS level.

Spin State	S = 1/2	S = 3/2
Exchange coupling	Anti-ferromagnetic	Ferromagnetic
Mulliken Spin moment of Fe (μ_B)	1.73	2.59
Mulliken Spin moment of Ni (μ_B)	-0.32	0.97
Mulliken Spin moment of $NiCp_2$ (μ_B)	-0.33	1.00
Mulliken Charge on $NiCp_2$ δ(e)	-0.43	-0.43
Transition Energy(cm^{-1}) by CASSCF	0.0	+5.6
Transition Energy(cm^{-1}) by NEVPT2	0.0	+14.7
Transition Energy(cm^{-1})by PBE0-D3	+97.9	0.0

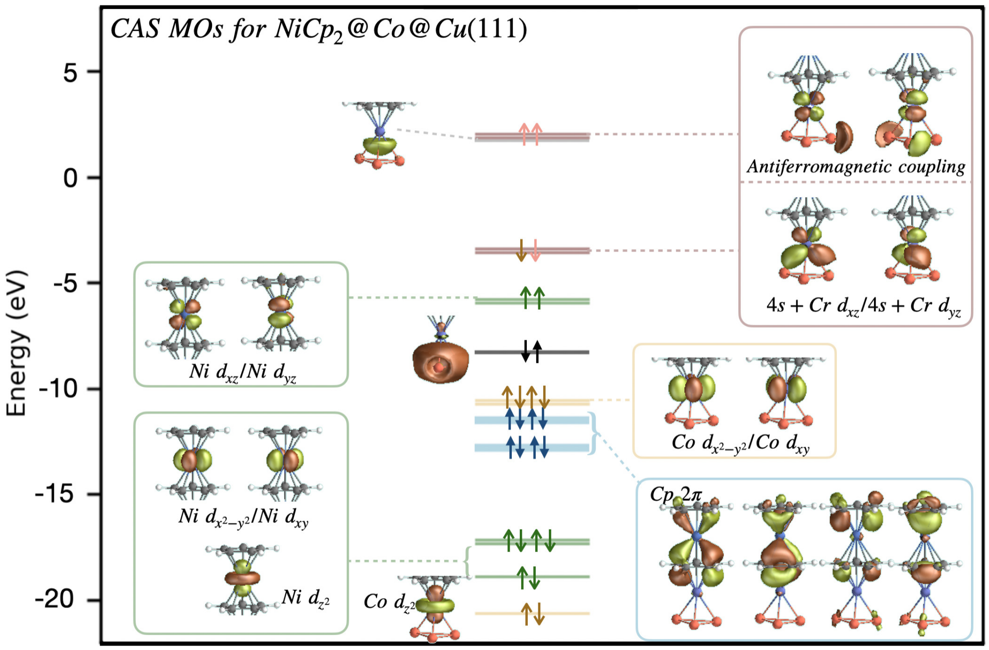

Figure S31: CASSCF MOs diagrams for NiCp$_2$@Co@Cu(111) small model.

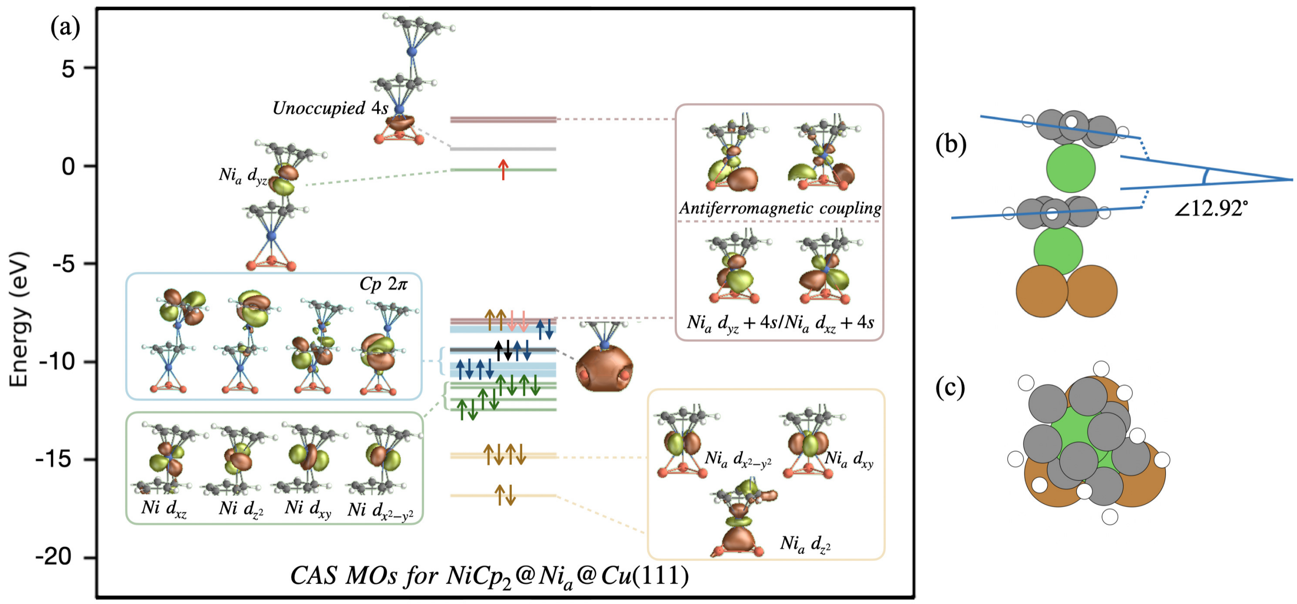

Figure S32: CASSCF MOs diagrams for NiCp$_2$@Ni@Cu(111) small model.

S.4.6 Comparisons between non-distorted and distorted geometries and electronic structure

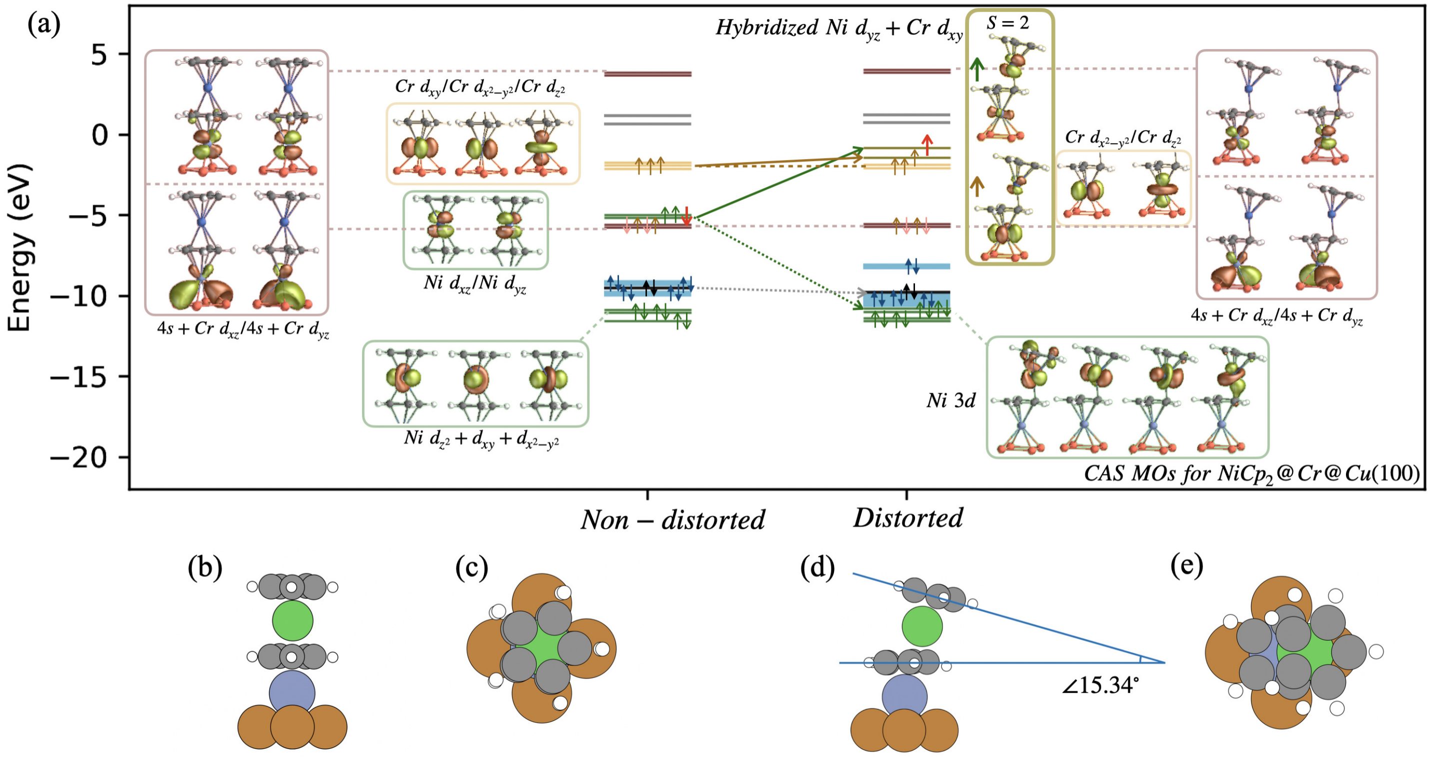

Figure S33: (a) CASSCF MOs diagrams for $NiCp_2$@Cr@Cu(100) small models. (b) Side view and (c) top view of the non-tilted configuration, optimized at the $S = 1$ spin state. (d) Side view and (e) top view of the distorted configuration, optimized at the $S = 2$ spin state. All geometries are optimized at the PBE0-D3 level of theory in ORCA. $S = 2$ is always the ground spin state for both configurations.

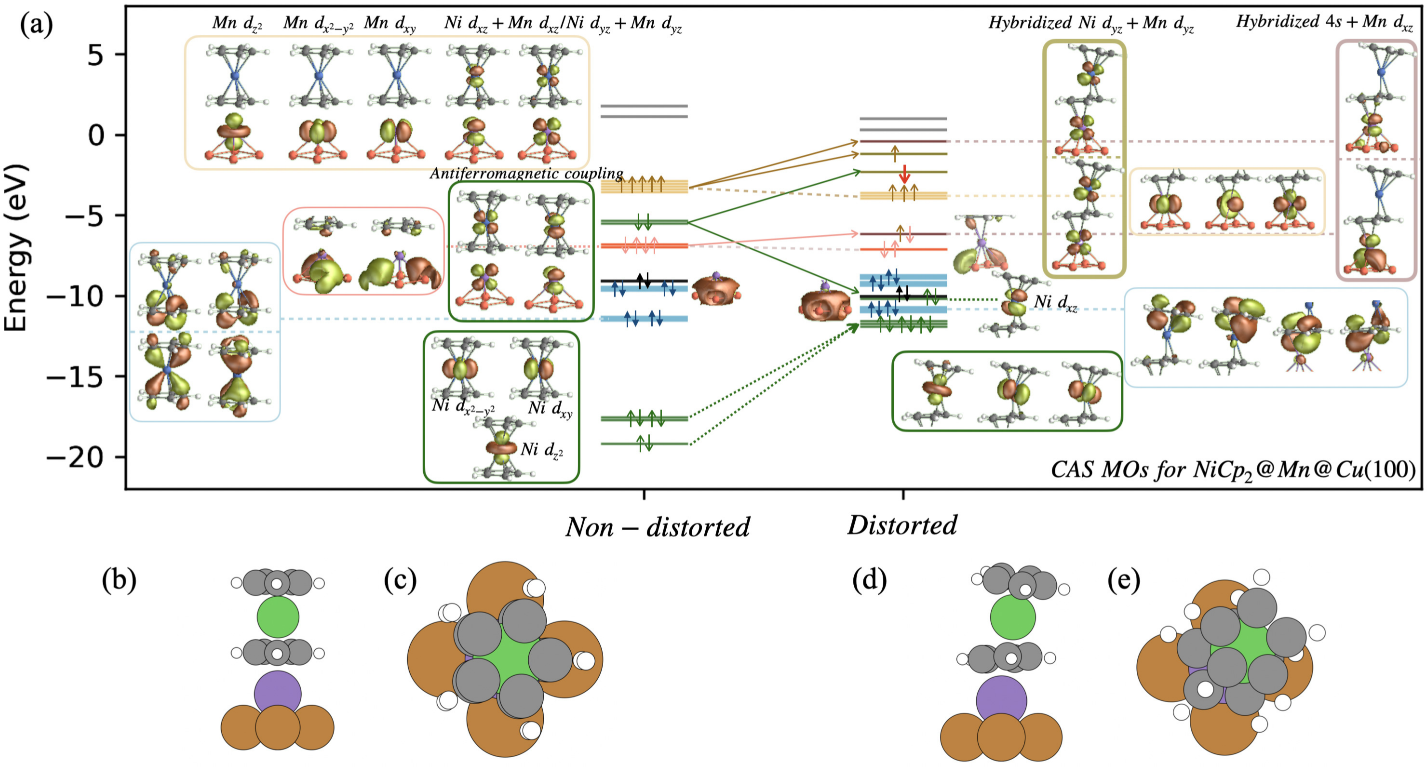

Figure S34: (a) CASSCF molecular orbital (MO) diagrams for $NiCp_2$@Mn@Cu(100) small models. (b) Side view and (c) top view of the non-tilted configuration, optimized at the $S = 3/2$ spin state. (d) Side view and (e) top view of the distorted configuration, optimized at the $S = 5/2$ spin state. All geometries are optimized at the PBE0-D3 level of theory in ORCA. Despite the distorted configuration being obtained at $S = 5/2$, the $S = 3/2$ spin state (antiferromagnetic coupling) remains the ground state for both configurations, as consistently predicted by PBE0-D3 and CASSCF(NEVPT2) calculations.

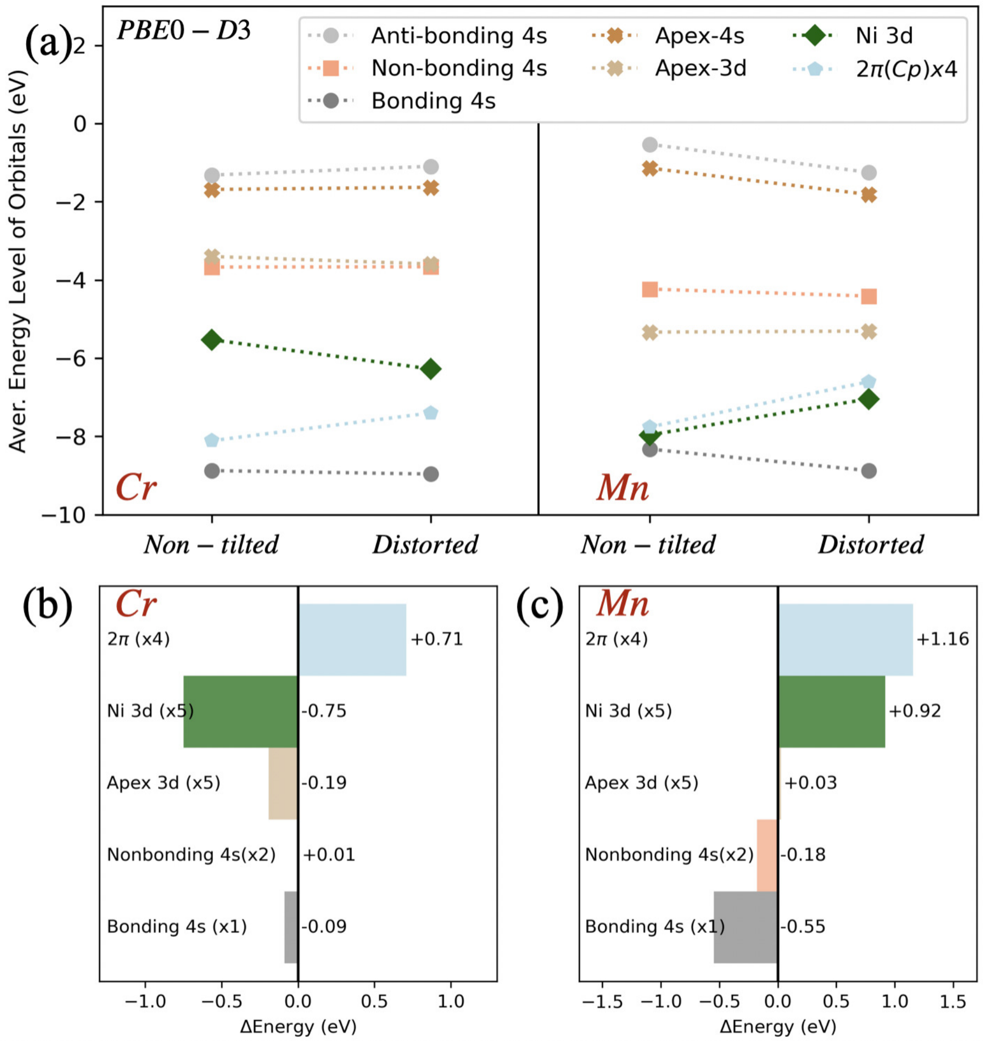

Figure S35: (a) The average energy levels of 3d and 4s orbitals for $NiCp_2$@Cr@Cu(100) and $NiCp_2$@Mn@Cu(100) small models. In the Cr@Cu(100) case, the distorted $NiCp_2$ configuration is the ground state, whereas in the Mn@Cu(100) case, the non-distorted configuration is energetically favored. (b) Energy level shifts of Ni 3d orbitals during the transition from the non-tilted to the distorted $NiCp_2$ configuration in the Cr@Cu(100) system. (c) The corresponding energy level changes for the Mn-Cu(100) system. All calculations were conducted at the PBE0-D3 level of theory.

Table S9: The comparison of the $NiCp_2$@Cr@Cu(100) small model optimized at different spin states ($S = 1$ and $S = 2$) using the PBE0-D3 level of theory in ORCA, resulting in two distinct geometries. The data is divided into two sections: the upper part presents results from PBE0-D3 calculations, while the lower part displays results obtained at the CASSCF level of theory. At PBE0-D3 level, the non-distorted case has a $S = 1$ ground state, while the distorted one has a $S = 2$ ground state. At CAS(NEVPT2)-level, the $S = 2$ state with a ferromagnetic coupling is recognized as the ground state in both configuration. The corresponding geometries are illustrated in Figure S33.

Optimized at Different Spin State	$S = 1$	$S = 2$
Optimized geometry	Non-distorted $NiCp_2$	Distorted $NiCp_2$
PBE0-D3, ORCA		
$E_{S=1}(a.u.)$	-9500.31986757	-9500.32545233
$E_{S=2}(a.u.)$	-9500.31893360	-9500.32746547
$E_{S=3}(a.u.)$	-9500.29827858	-9500.29108627
Ground Spin State	S = 1	S = 2
ΔE_{GS} (eV)	+0.21	0
Exchange coupling	Antiferromagnetic	Ferromagnetic
Mulliken Spin moment of Cr (μ_B)	4.44	4.30
Mulliken Spin moment of Ni (μ_B)	-1.00	0.86
Mulliken Spin moment of NiCp2(μ_B)	-1.47	0.78
Mulliken Charge on $NiCp_2$ δ(e)	0.01	-0.10
CASSCF, ORCA		
$E_{S=1}(a.u.)$ CASSCF	-9490.451766	-9490.451242
$E_{S=2}(a.u.)$ CASSCF	-9490.452954	-9490.452088
$Transition\ Energy(cm^{-1})$ by CASSCF	255.5 (Ferro-)	145.5 (Ferro-)
$E_{S=1}(a.u.)$ NEVPT2	-9495.933672	-9495.956094
$E_{S=2}(a.u.)$ NEVPT2	-9495.935770	-9495.957451
$Transition\ Energy(cm^{-1})$ by NEVPT2	204.9 (Ferro-)	152.3 (Ferro-)
Mulliken Spin moment of Cr (μ_B)	3.52	3.49
Mulliken Spin moment of Ni (μ_B)	0.94	0.95
Mulliken Spin moment of NiCp2(μ_B)	0.95	0.97
Mulliken Charge on $NiCp_2$ δ(e)	-0.33	-0.34

S.4.7 The PDOS for the pyramid models

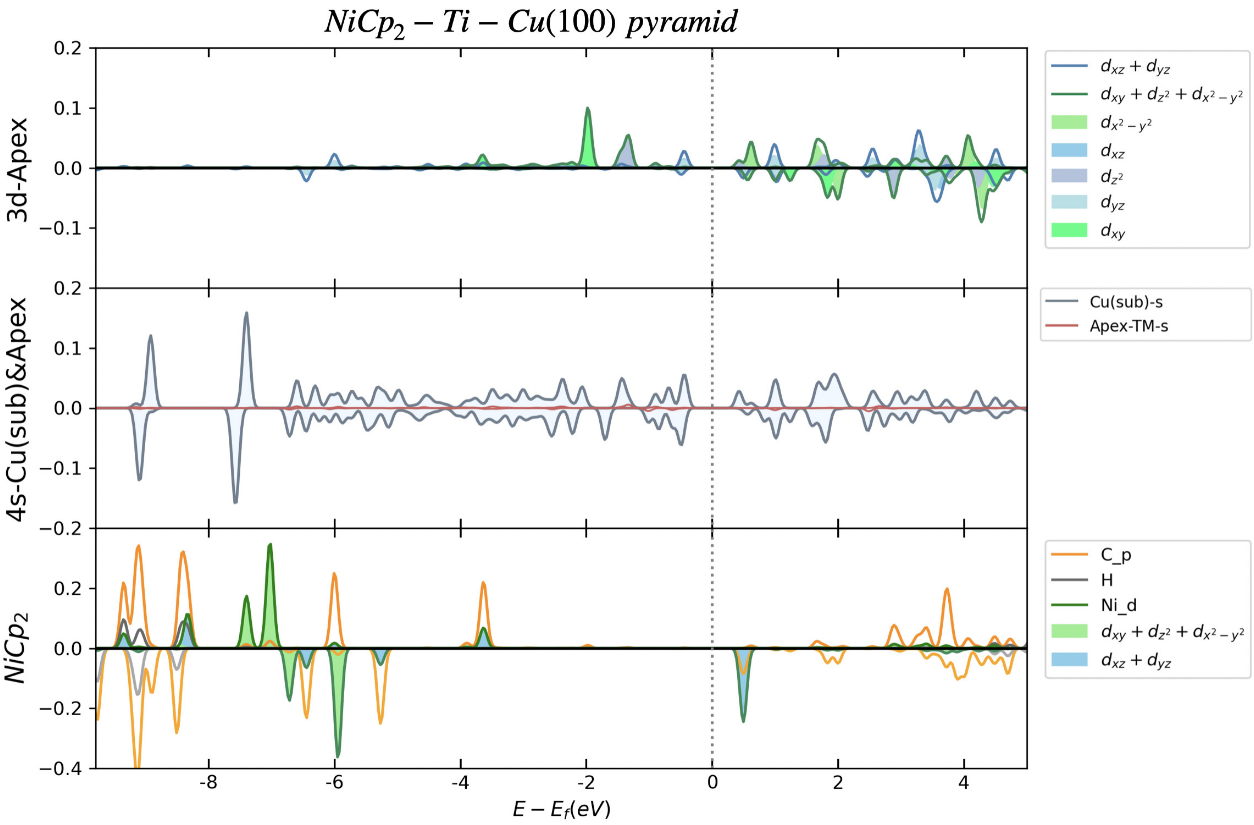

Figure S36: The Projected DOS for $NiCp_2$@Ti@Cu(100) large model: (a) the 3d states of the apex element, (b) the 4s states for the apex element (red) and the Cu substrate (blue), (d) Ni-3d, C-2p and H-s states of $NiCp_2$ molecule.

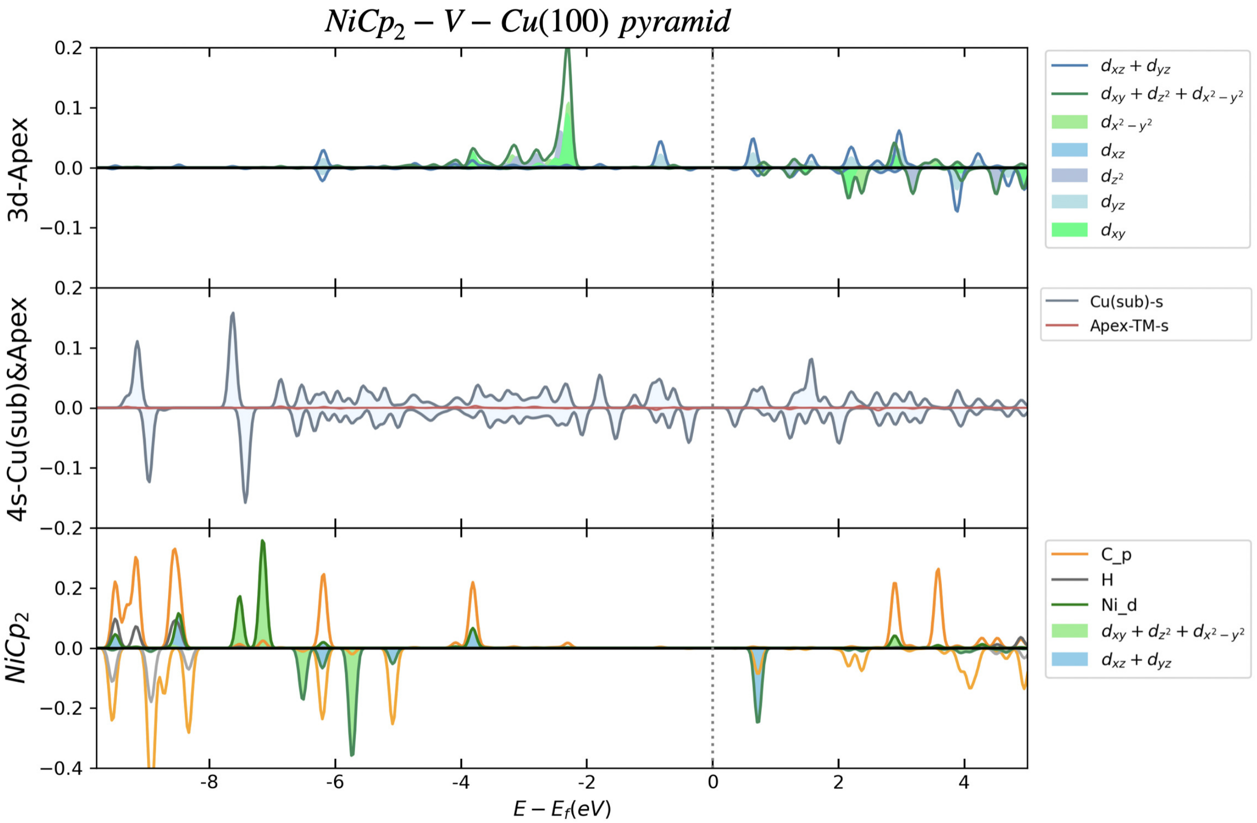

Figure S37: The Projected DOS for $NiCp_2$@V@Cu(100) large model: (a) the 3d states of the apex element, (b) the 4s states for the apex element (red) and the Cu substrate (blue), (d) Ni-3d, C-2p and H-s states of $NiCp_2$ molecule.

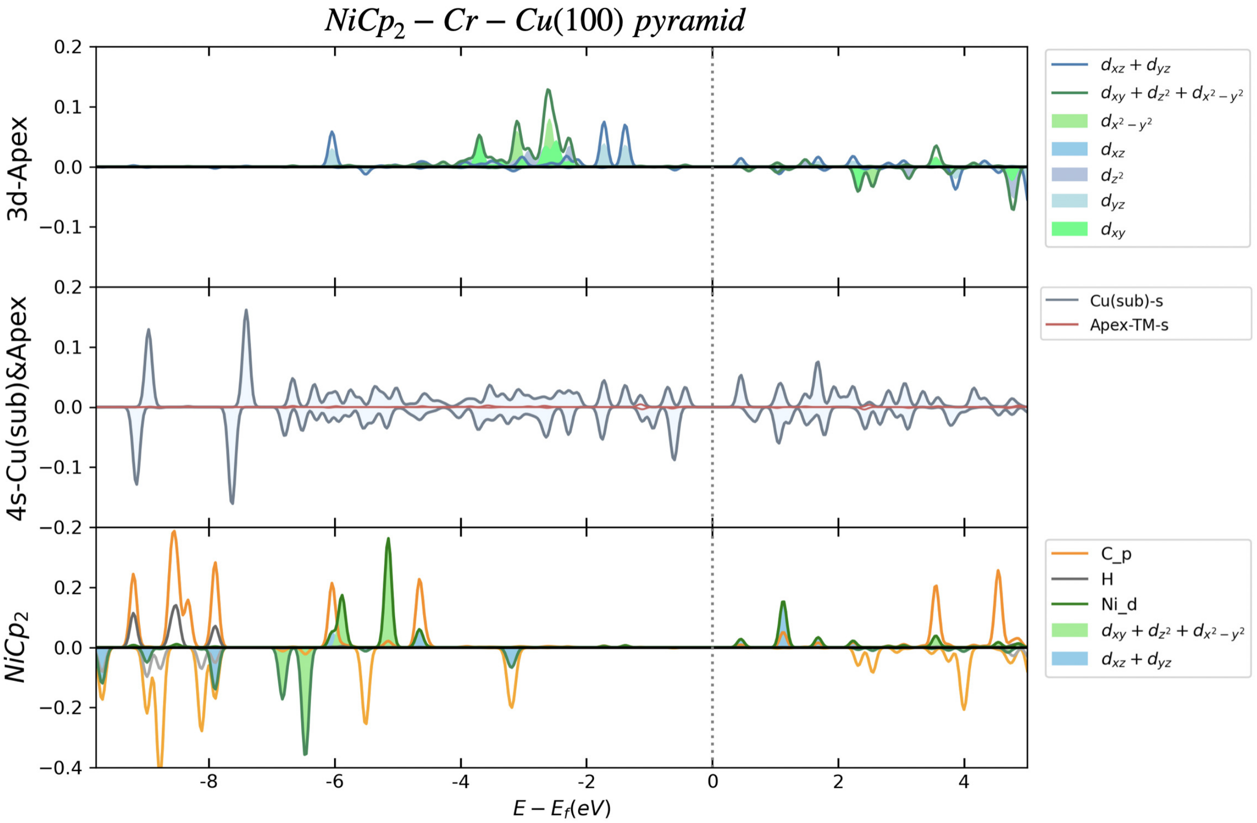

Figure S38: The Projected DOS for $NiCp_2$@Cr@Cu(100) large model: (a) the 3d states of the apex element, (b) the 4s states for the apex element (red) and the Cu substrate (blue), (d) Ni-3d, C-2p and H-s states of $NiCp_2$ molecule.

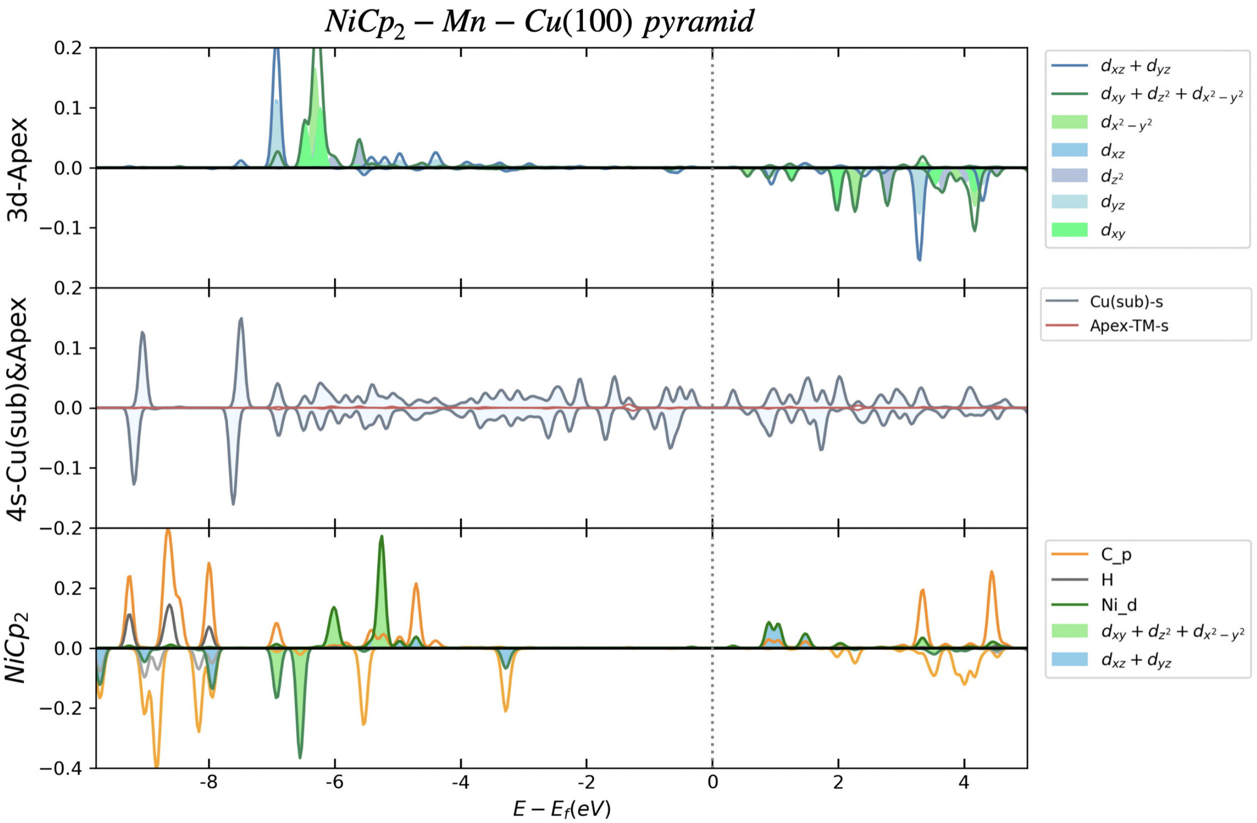

Figure S39: The Projected DOS for $NiCp_2$@Mn@Cu(100) large model: (a) the 3d states of the apex element, (b) the 4s states for the apex element (red) and the Cu substrate (blue), (d) Ni-3d, C-2p and H-s states of $NiCp_2$ molecule.

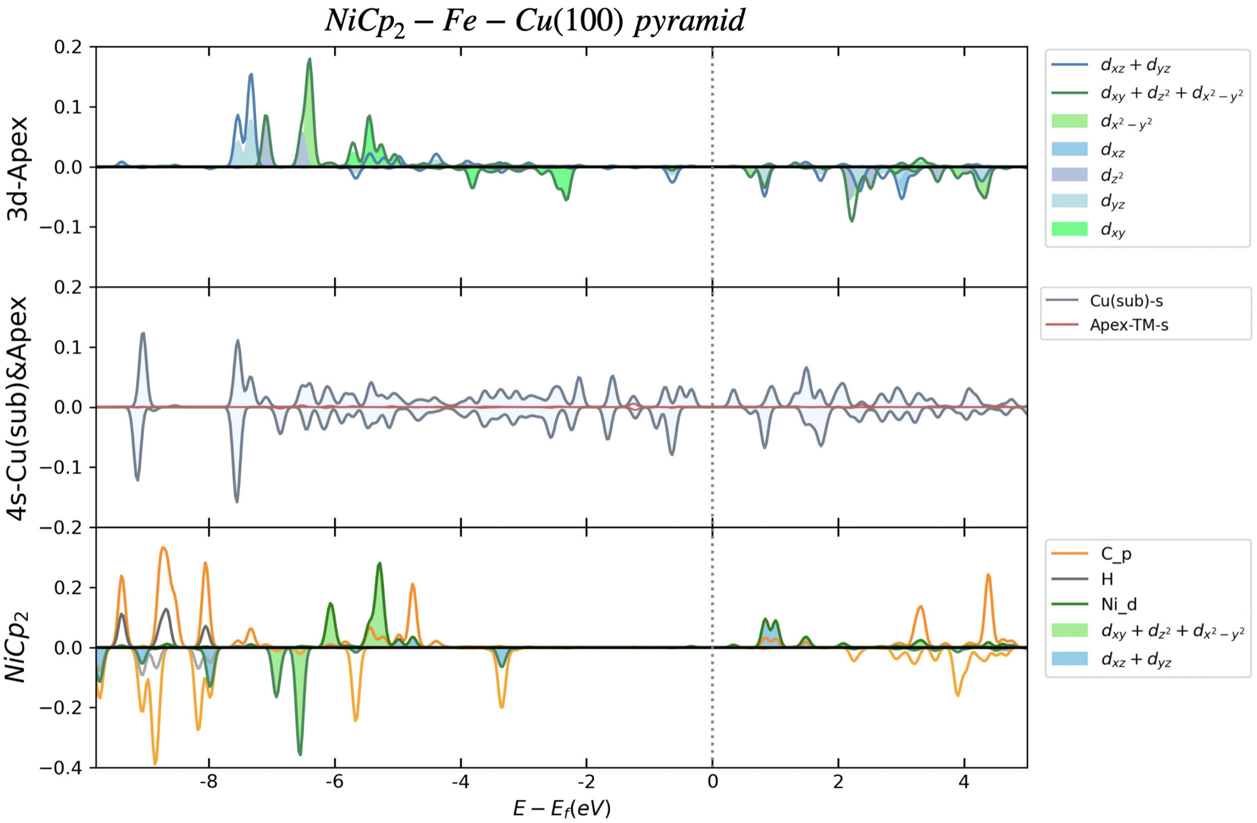

Figure S40: The Projected DOS for $NiCp_2$@Fe@Cu(100) large model: (a) the 3d states of the apex element, (b) the 4s states for the apex element (red) and the Cu substrate (blue), (d) Ni-3d, C-2p and H-s states of $NiCp_2$ molecule.

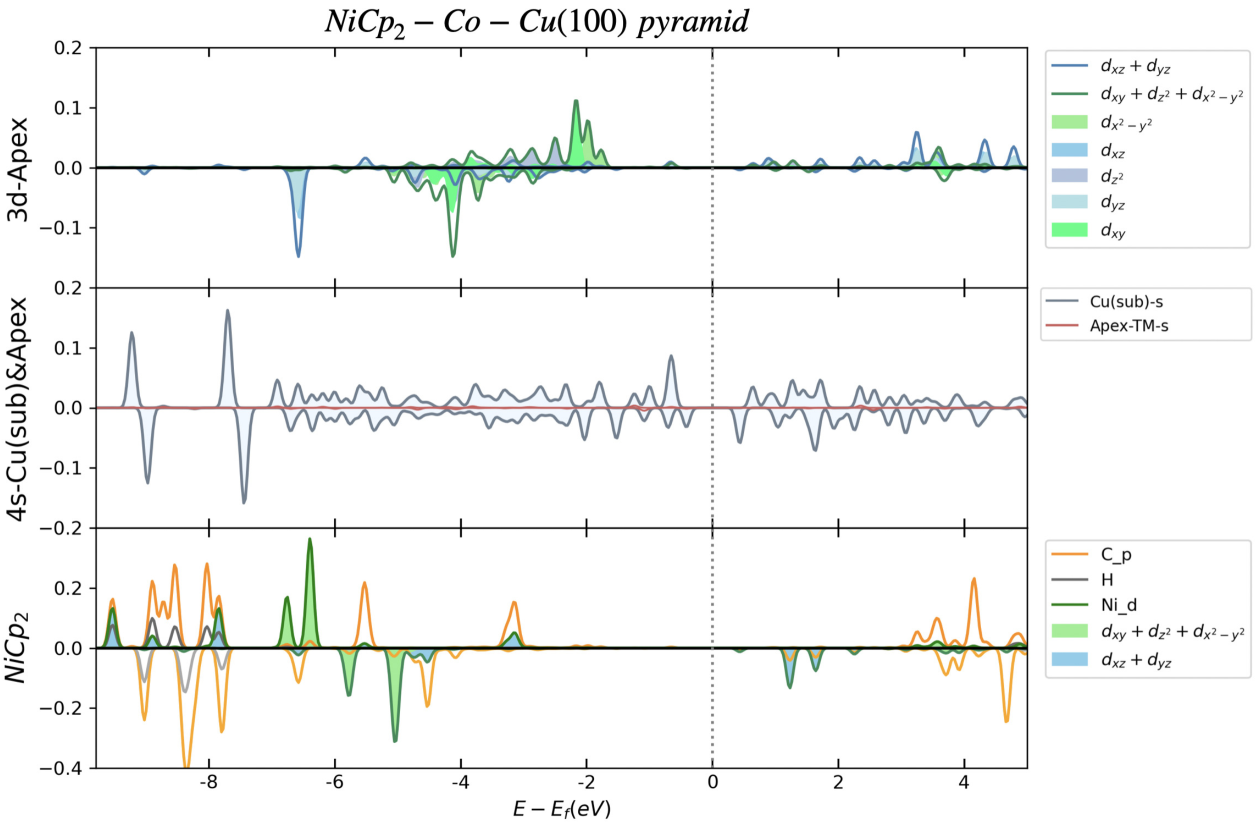

Figure S41: The Projected DOS for $NiCp_2$@Co@Cu(100) large model: (a) the 3d states of the apex element, (b) the 4s states for the apex element (red) and the Cu substrate (blue), (d) Ni-3d, C-2p and H-s states of $NiCp_2$ molecule.

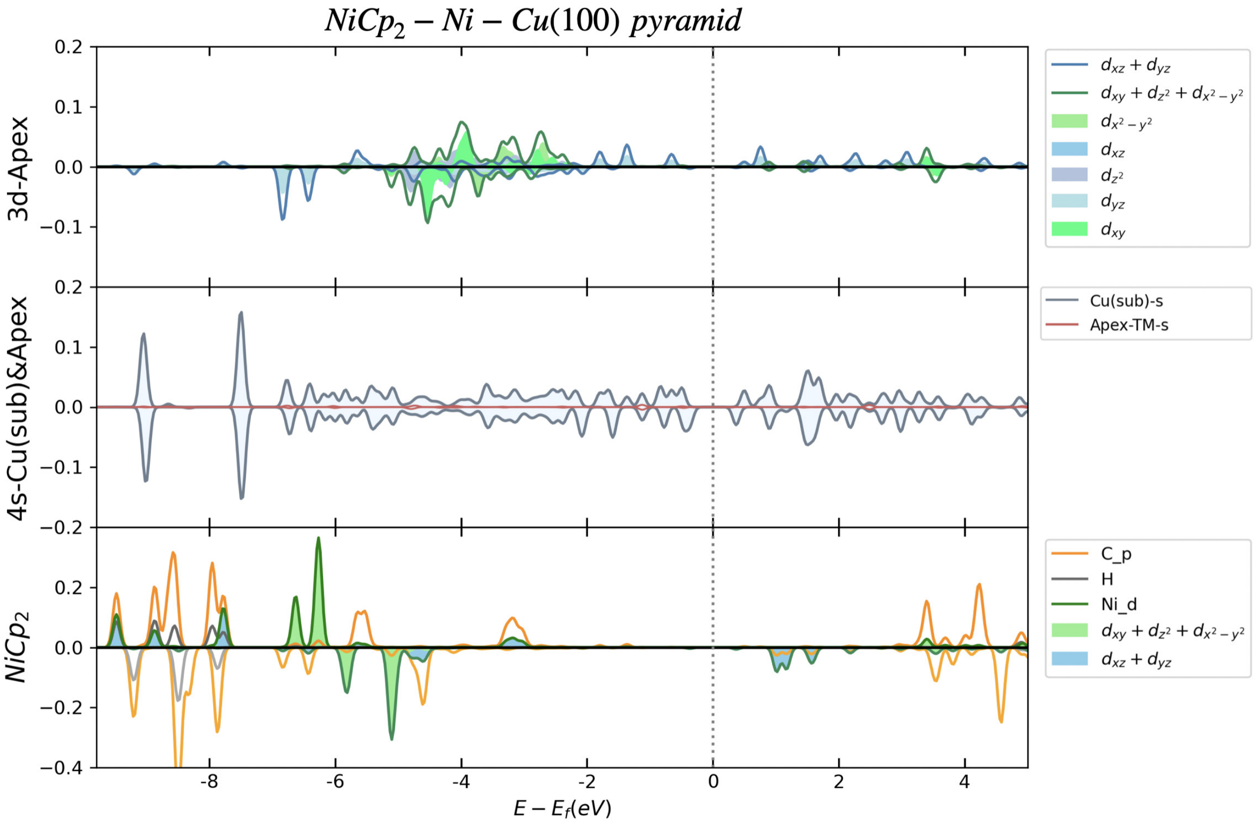

Figure S42: The Projected DOS for $NiCp_2$@Ni@Cu(100) large model: (a) the 3d states of the apex element, (b) the 4s states for the apex element (red) and the Cu substrate (blue), (d) Ni-3d, C-2p and H-s states of $NiCp_2$ molecule.

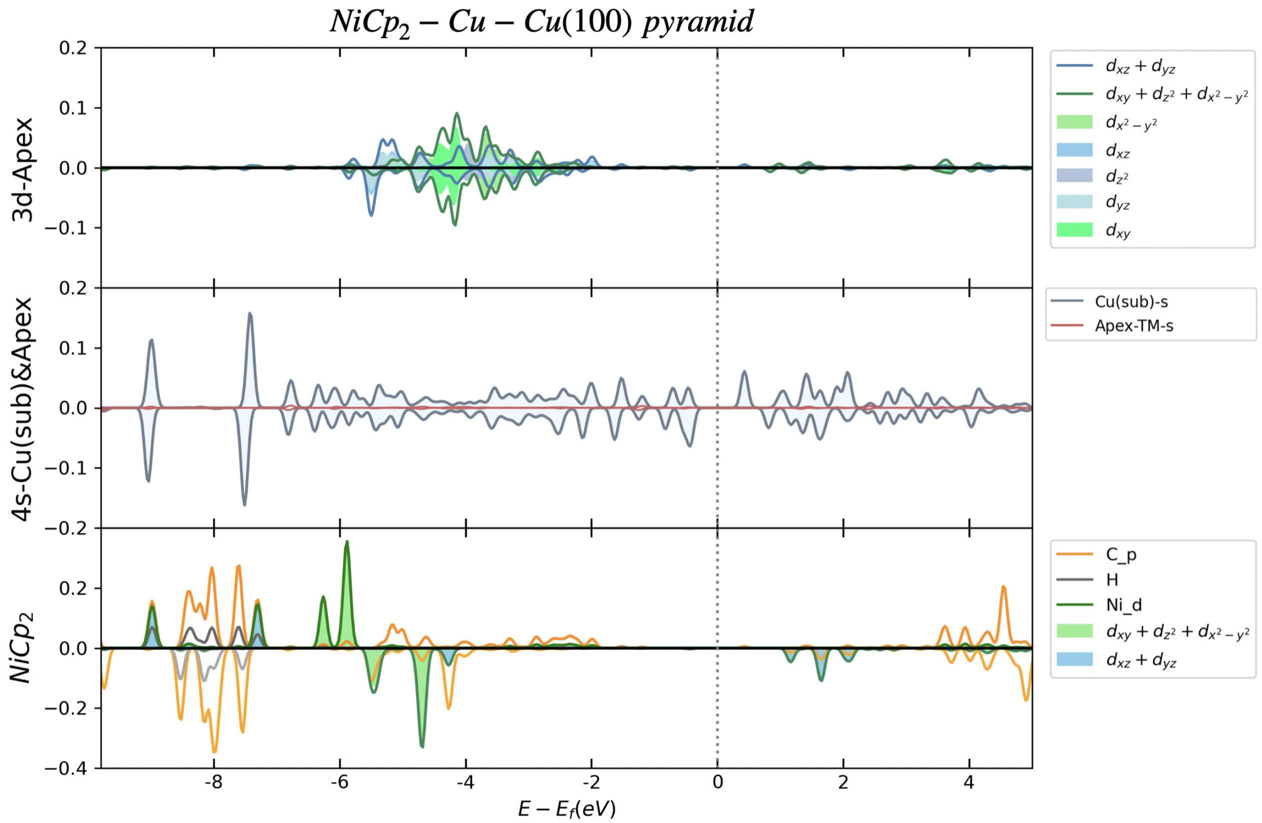

Figure S43: The Projected DOS for $NiCp_2$@Cu@Cu(100) large model: (a) the 3d states of the apex element, (b) the 4s states for the apex element (red) and the Cu substrate (blue), (d) Ni-3d, C-2p and H-s states of $NiCp_2$ molecule.

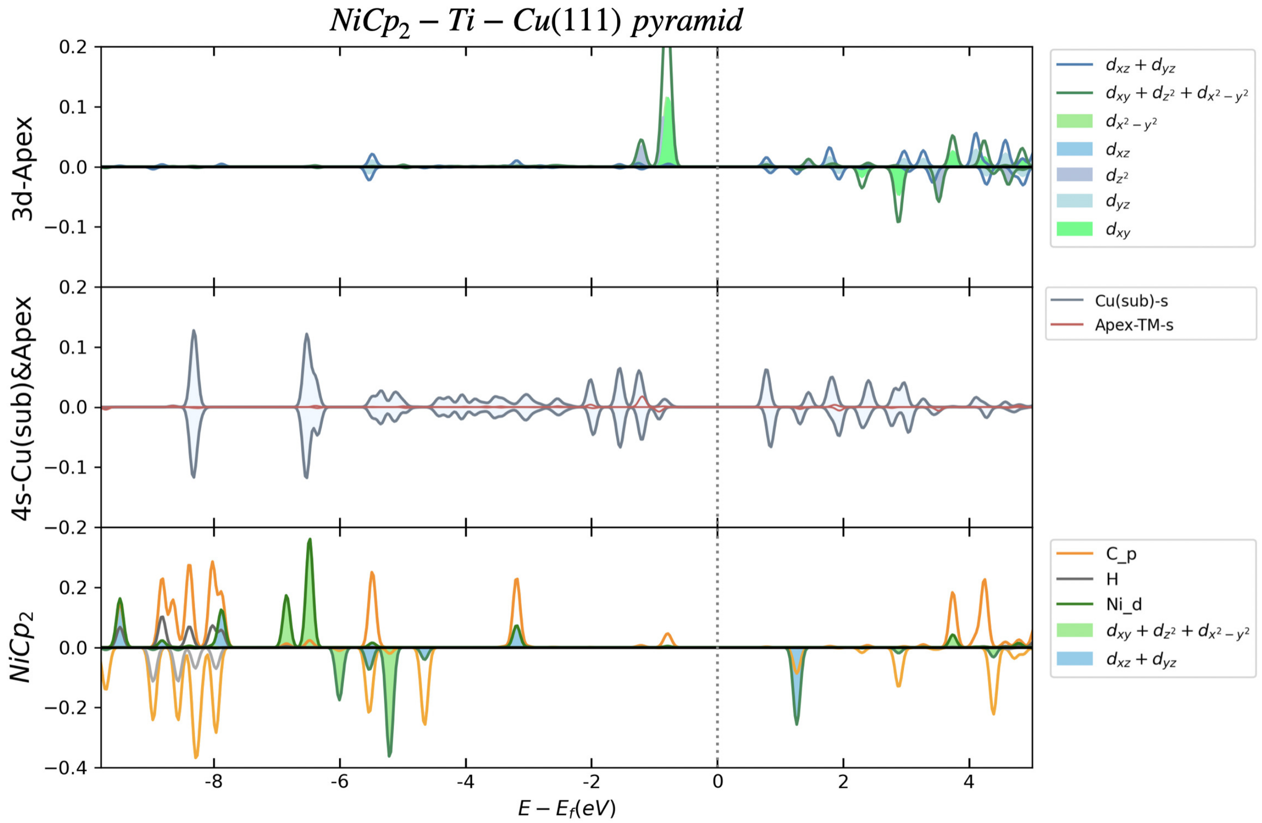

Figure S44: The Projected DOS for $NiCp_2$@Ti@Cu(111) large model: (a) the 3d states of the apex element, (b) the 4s states for the apex element (red) and the Cu substrate (blue), (d) Ni-3d, C-2p and H-s states of $NiCp_2$ molecule.

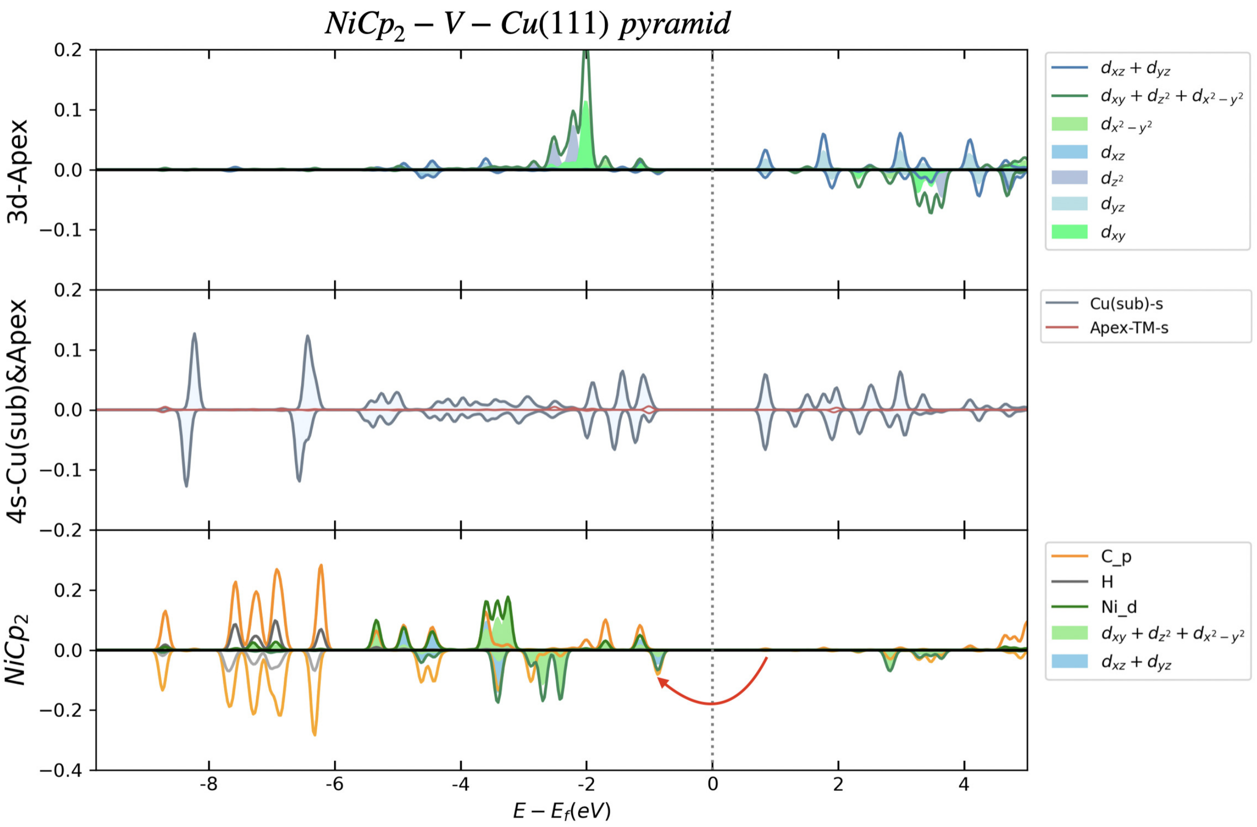

Figure S45: The Projected DOS for $NiCp_2$@V@Cu(111) large model: (a) the 3d states of the apex element, (b) the 4s states for the apex element (red) and the Cu substrate (blue), (d) Ni-3d, C-2p and H-s states of $NiCp_2$ molecule. The red arrow indicates one electron is relocated into the Ni-3d, reducing the spin moment of Ni.

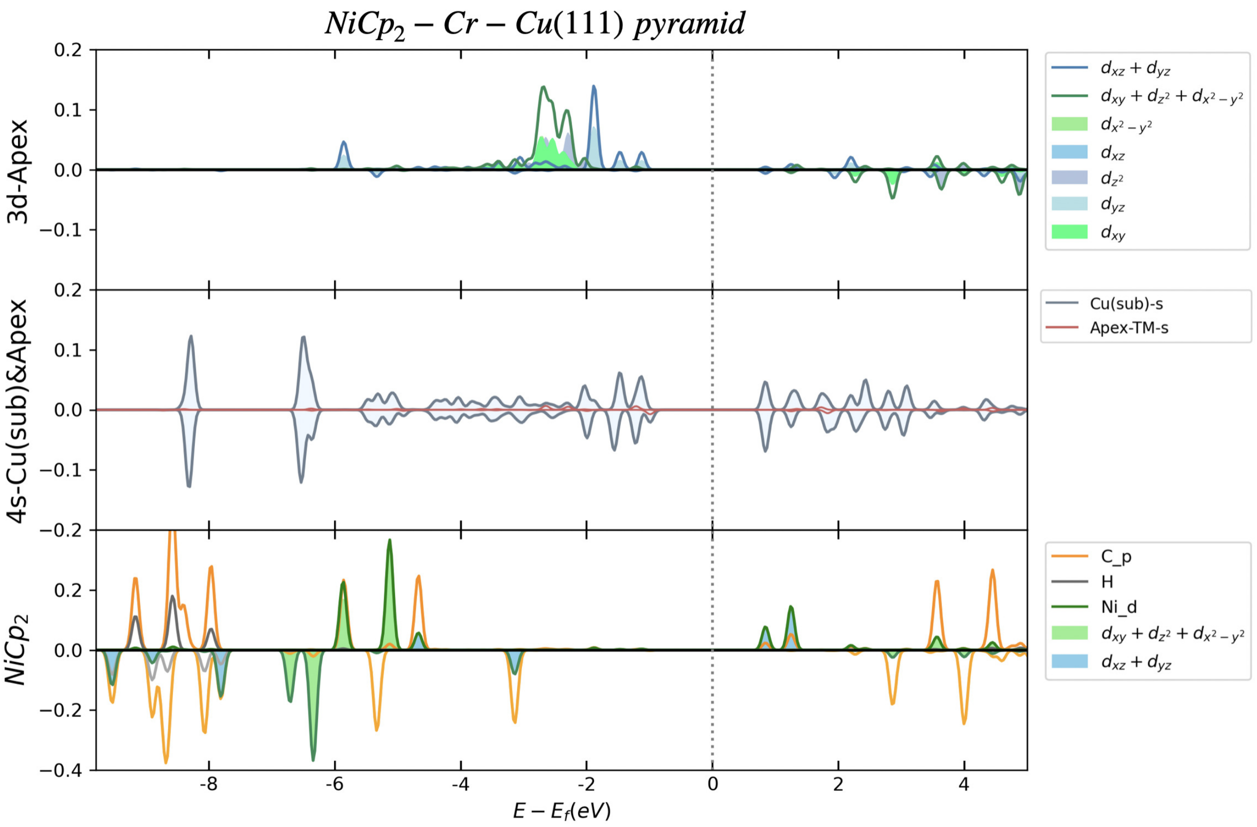

Figure S46: The Projected DOS for $NiCp_2$@Cr@Cu(111) large model: (a) the 3d states of the apex element, (b) the 4s states for the apex element (red) and the Cu substrate (blue), (d) Ni-3d, C-2p and H-s states of $NiCp_2$ molecule.

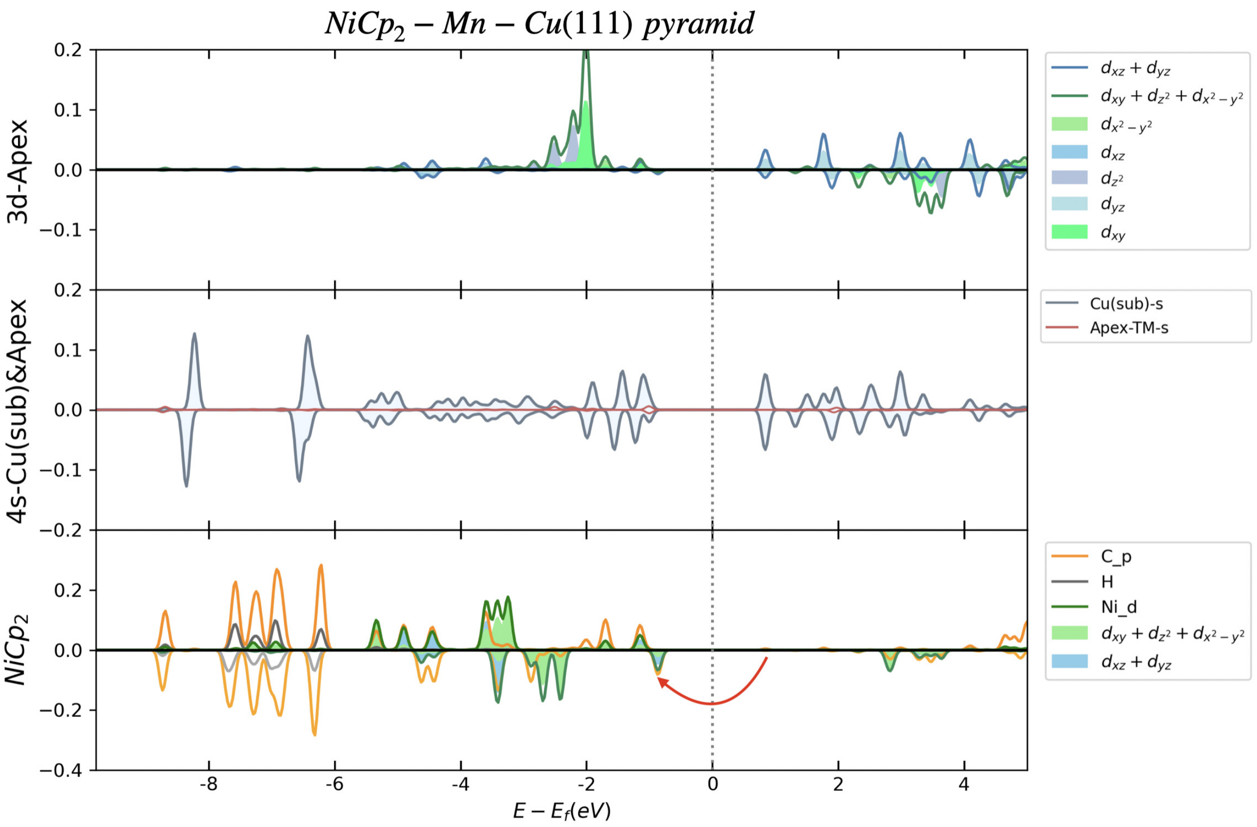

Figure S47: The Projected DOS for $NiCp_2$@Mn@Cu(111) large model: (a) the 3d states of the apex element, (b) the 4s states for the apex element (red) and the Cu substrate (blue), (d) Ni-3d, C-2p and H-s states of $NiCp_2$ molecule. The red arrow indicates one more electron is relocated into the Ni-3d, reducing the spin moment of Ni.

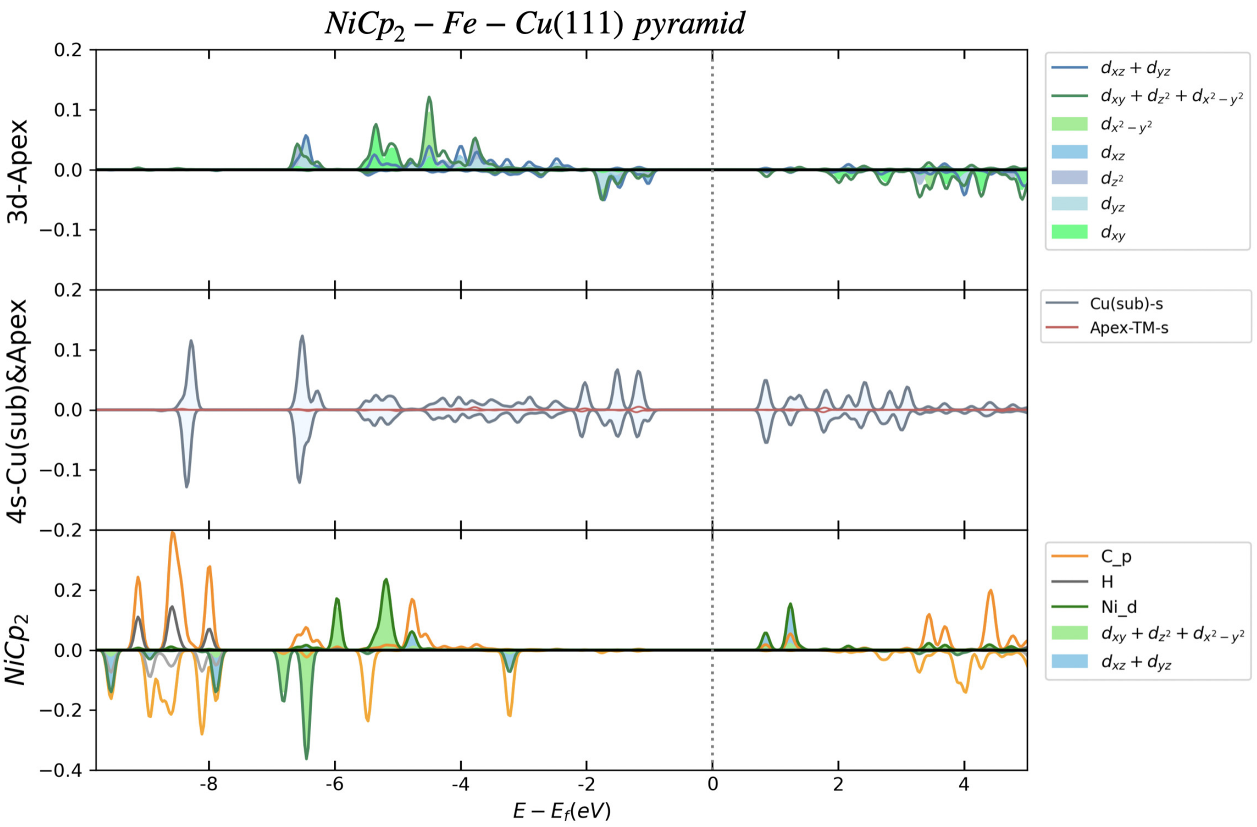

Figure S48: The Projected DOS for $NiCp_2$@Fe@Cu(111) large model: (a) the 3d states of the apex element, (b) the 4s states for the apex element (red) and the Cu substrate (blue), (d) Ni-3d, C-2p and H-s states of $NiCp_2$ molecule.

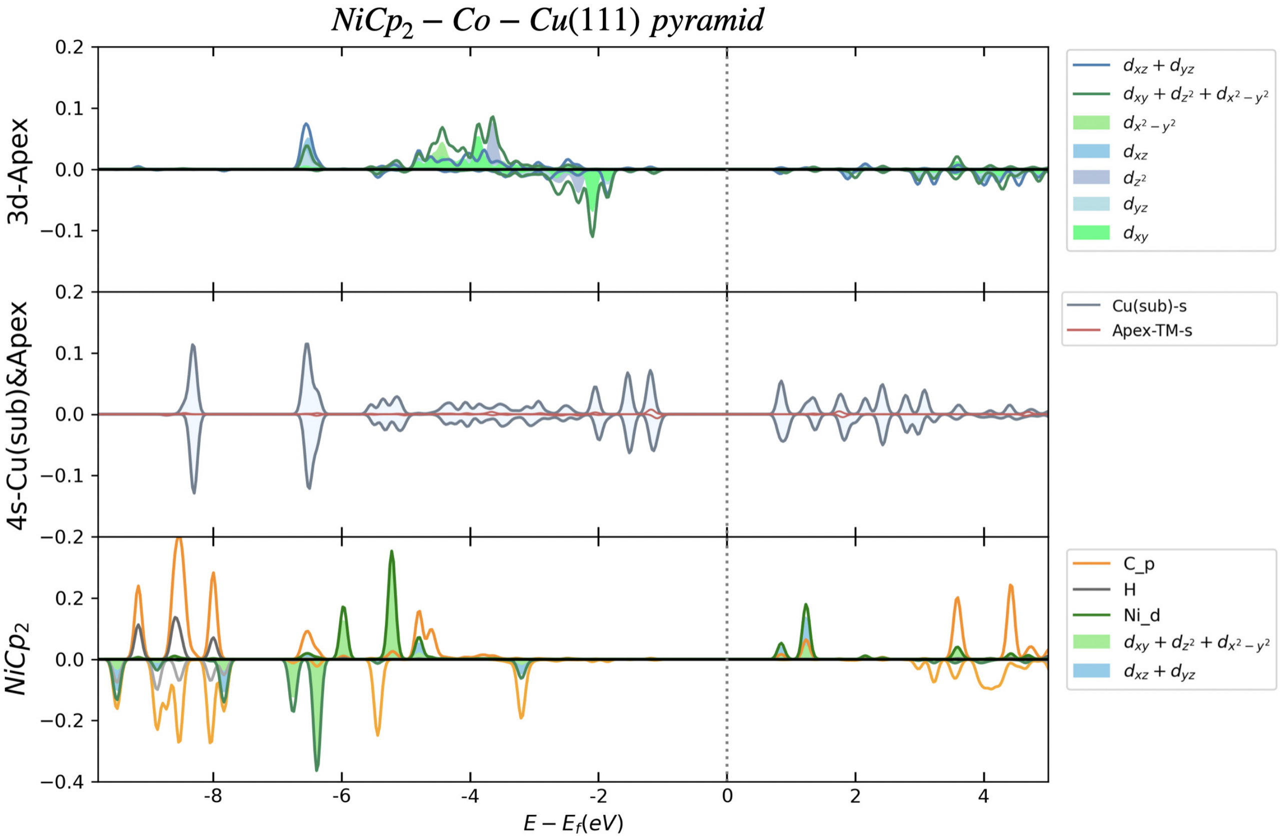

Figure S49: The Projected DOS for $NiCp_2$@Co@Cu(111) large model: (a) the 3d states of the apex element, (b) the 4s states for the apex element (red) and the Cu substrate (blue), (d) Ni-3d, C-2p and H-s states of $NiCp_2$ molecule.

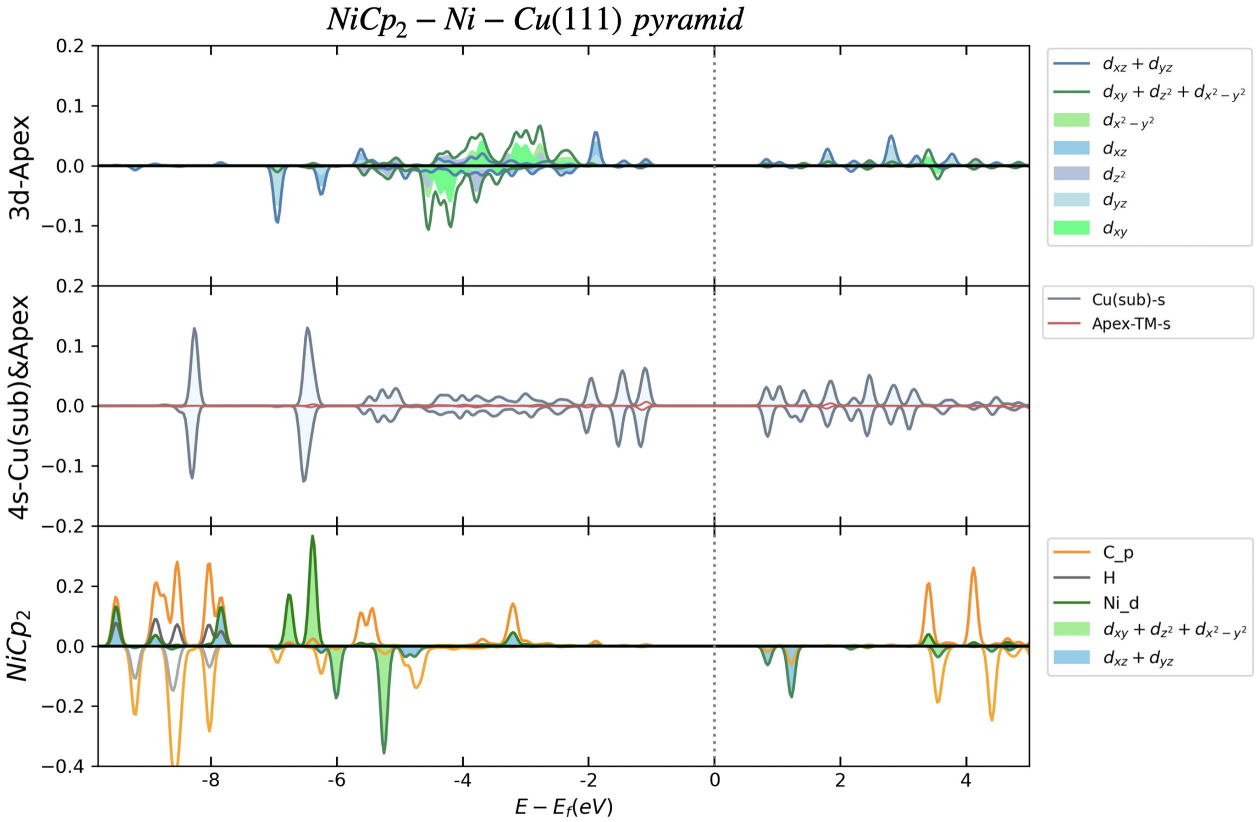

Figure S50: The Projected DOS for $NiCp_2$@Ni@Cu(111) large model: (a) the 3d states of the apex element, (b) the 4s states for the apex element (red) and the Cu substrate (blue), (d) Ni-3d, C-2p and H-s states of $NiCp_2$ molecule.

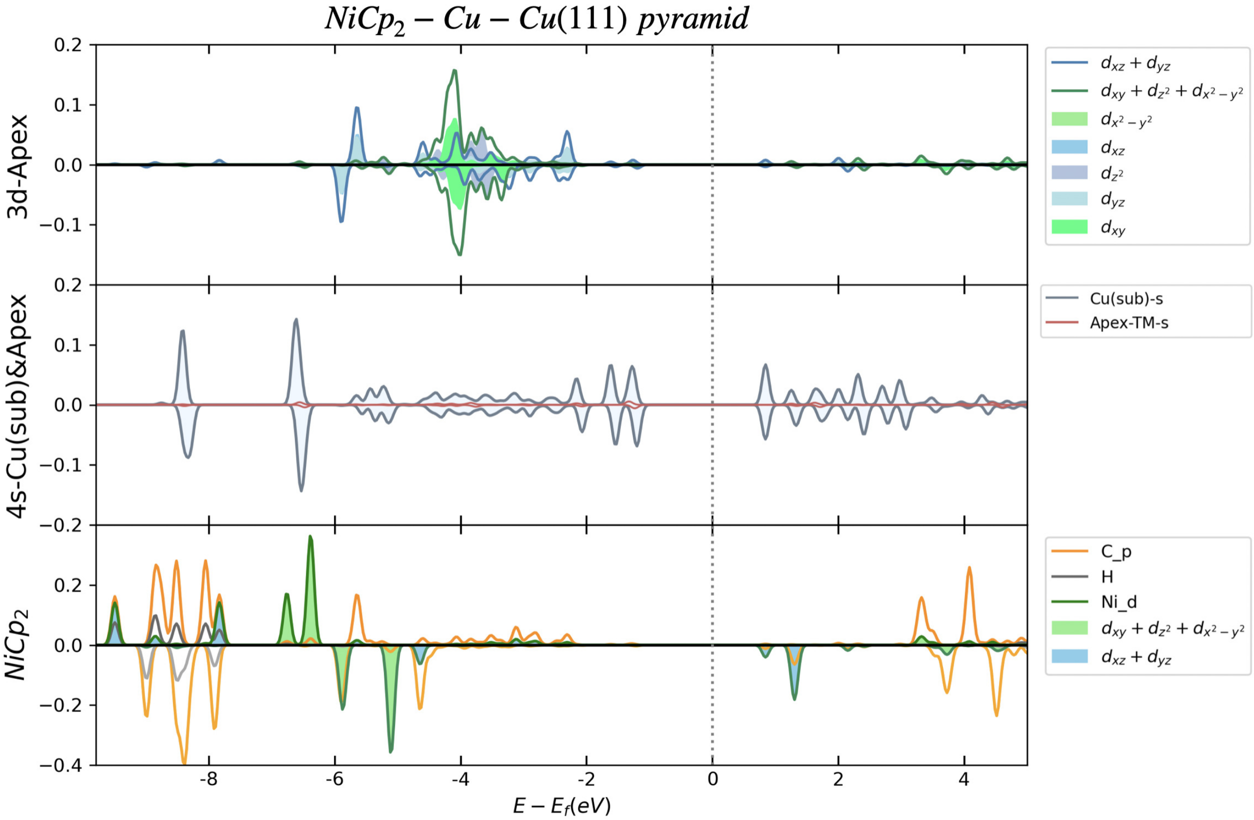

Figure S51: The Projected DOS for $NiCp_2$@Cu@Cu(111) large model: (a) the 3d states of the apex element, (b) the 4s states for the apex element (red) and the Cu substrate (blue), (d) Ni-3d, C-2p and H-s states of $NiCp_2$ molecule.

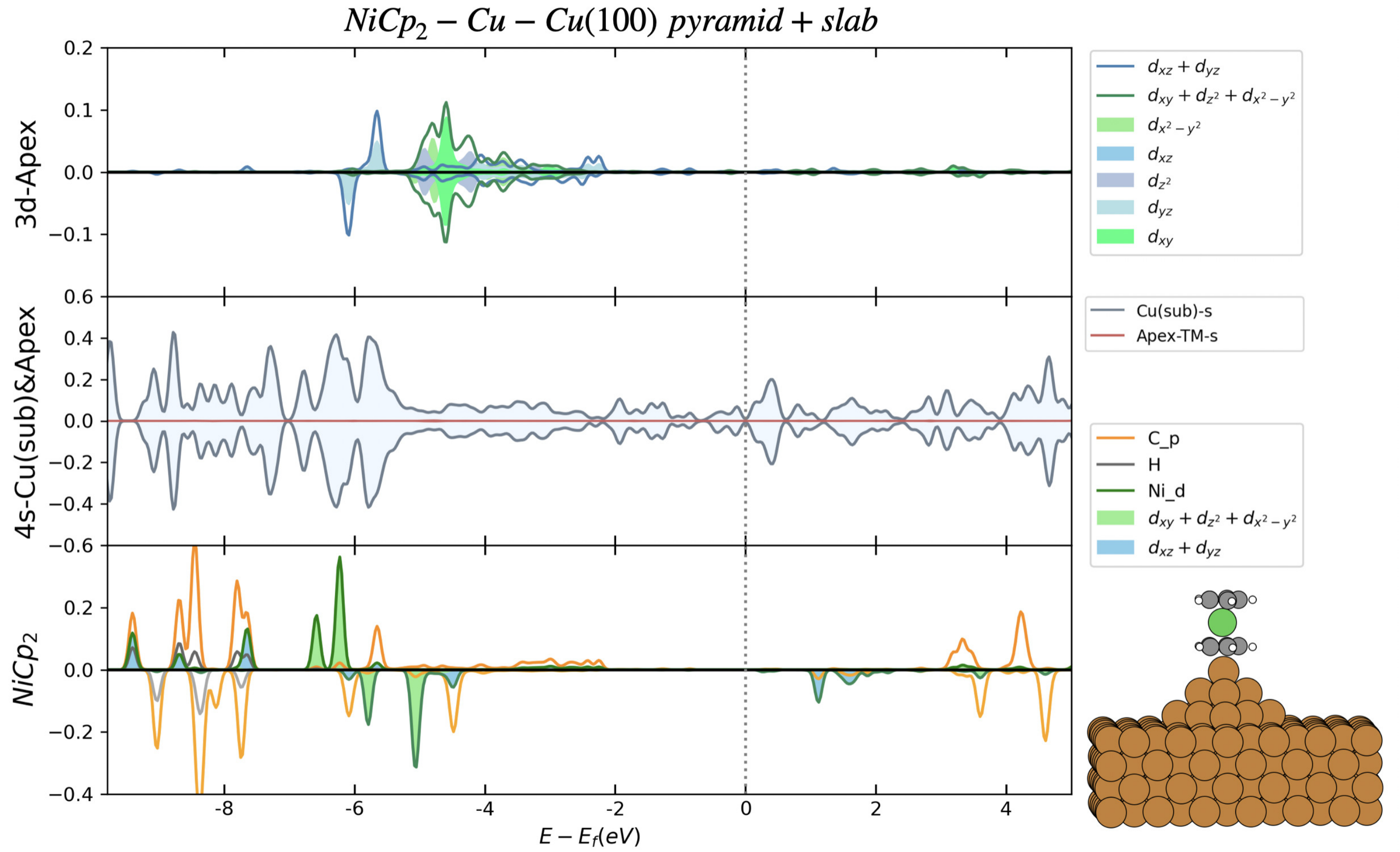

Figure S52: The Projected DOS for $NiCp_2$@Cu@Cu(100) large model with a slab: (a) the 3d states of the apex element, (b) the 4s states for the apex element (red) and the Cu substrate (blue), (d) Ni-3d, C-2p and H-s states of $NiCp_2$ molecule.

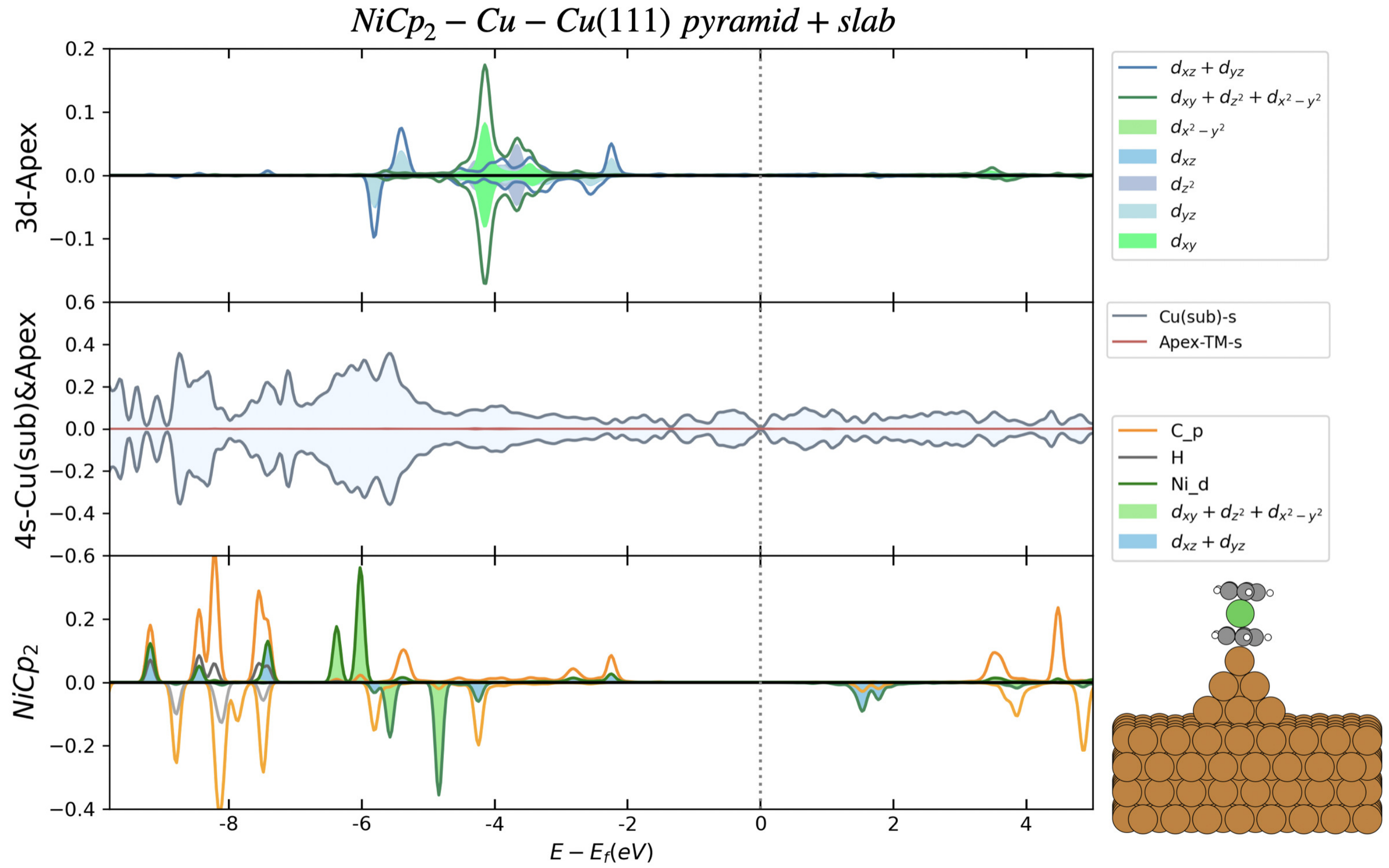

Figure S53: The Projected DOS for $NiCp_2$@Cu@Cu(111) large model with a slab: (a) the 3d states of the apex element, (b) the 4s states for the apex element (red) and the Cu substrate (blue), (d) Ni-3d, C-2p and H-s states of $NiCp_2$ molecule.

S.4.8 The relationship between β values and molecular spin

We computed a series of intermediate geometries along the optimization pathway of the $NiCp_2$@V@Cu(111) system and analyzed the relationship between the β angle and the total energy, molecular charge, and spin moment, comparing with the final optimization geometry. These results are shown in Figure S54(a), with the corresponding geometries illustrated in panel (e).

From the initial, non-distorted configuration, $NiCp_2$ is already reduced due to 3d orbital hybridization between V and Ni, as previously discussed. This interaction drives the system toward distortion, which lowers the total energy. As β increases, the total energy decreases, the charge on $NiCp_2$ drops, and the spin moment stabilizes around 0.9 μ_B. A similar trend is observed when Mn is used as the apex atom (Figure S54(b)). In this case, we retained the same geometries as in the V system (and applied the same approach to the other transition metals considered, as discussed below), simply replacing V with Mn along the scanning path. The optimized geometry for each system was obtained independently via full geometry optimization. As a result, the structures sampled along the scanning path may deviate from the final optimized geometry. It still indicates that $NiCp_2$@Mn@Cu(111) (panel(b)) prefers a distorted geometry.

On the other hand, for the Cu apex case (Figure S54(c)) by directly replacing V with Cu, the molecule does not favor a distorted geometry, and the optimized structure corresponds to a β angle close to zero. When increasing β, the total energy rises, accompanied by electron transfer to $NiCp_2$, resulting in partial reduction when β exceeds 15°. Another example is $NiCp_2$@V@Cu(100), shown in Figure S54(d), where the substrate is switched from Cu(111) to Cu(100) using the same scanning geometries (see panel (e)), and it favors a non-distorted geometry. As β increases, the spin moment of the molecule decreases from $S = 1$ to $S = 1/2$, with a sharp transition occurring around $\beta = 5°$. Beyond $\beta > 10°$, the spin remains nearly constant at $S = 1/2$.

Additionally, we extended the calculations to two large pyramidal systems, based on both Cu(111) and Cu(100) substrates, by adding two additional Cu layers beneath the small model (no further relaxation). The results for these extended structures are shown in Figure S55. The observed trends closely mirror those of the small models, confirming the consistency of this relationship across different model sizes and β values.

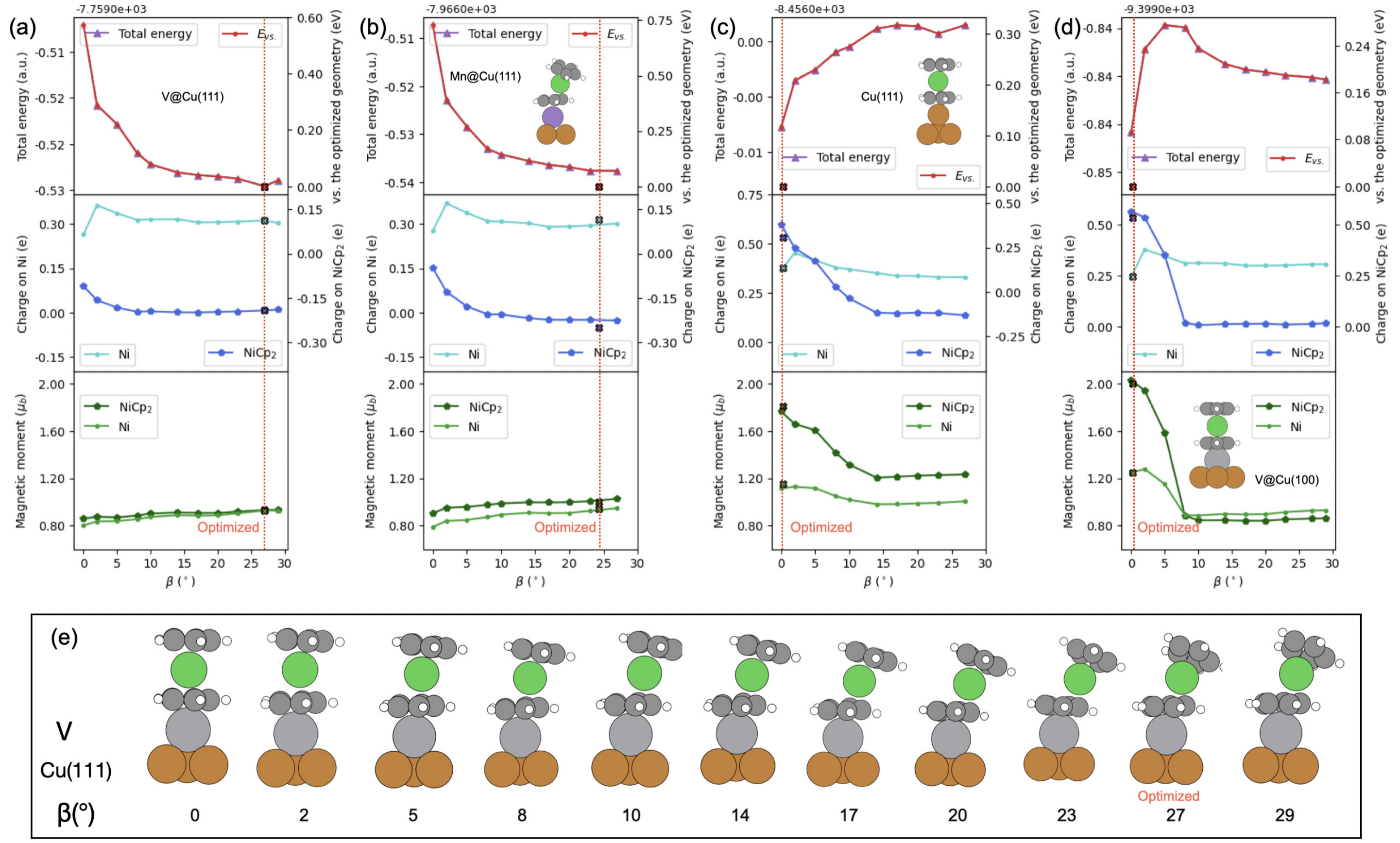

Figure S54: Energy, charge, and spin moment of $NiCp_2$ vs. β for (a) V, (b) Mn, (c) Cu apex on Cu(111), and (d) V on Cu(100). The corresponding geometries for panel (a) are shown in panel (e). Properties of the optimized geometries are indicated by crosses with black edges.

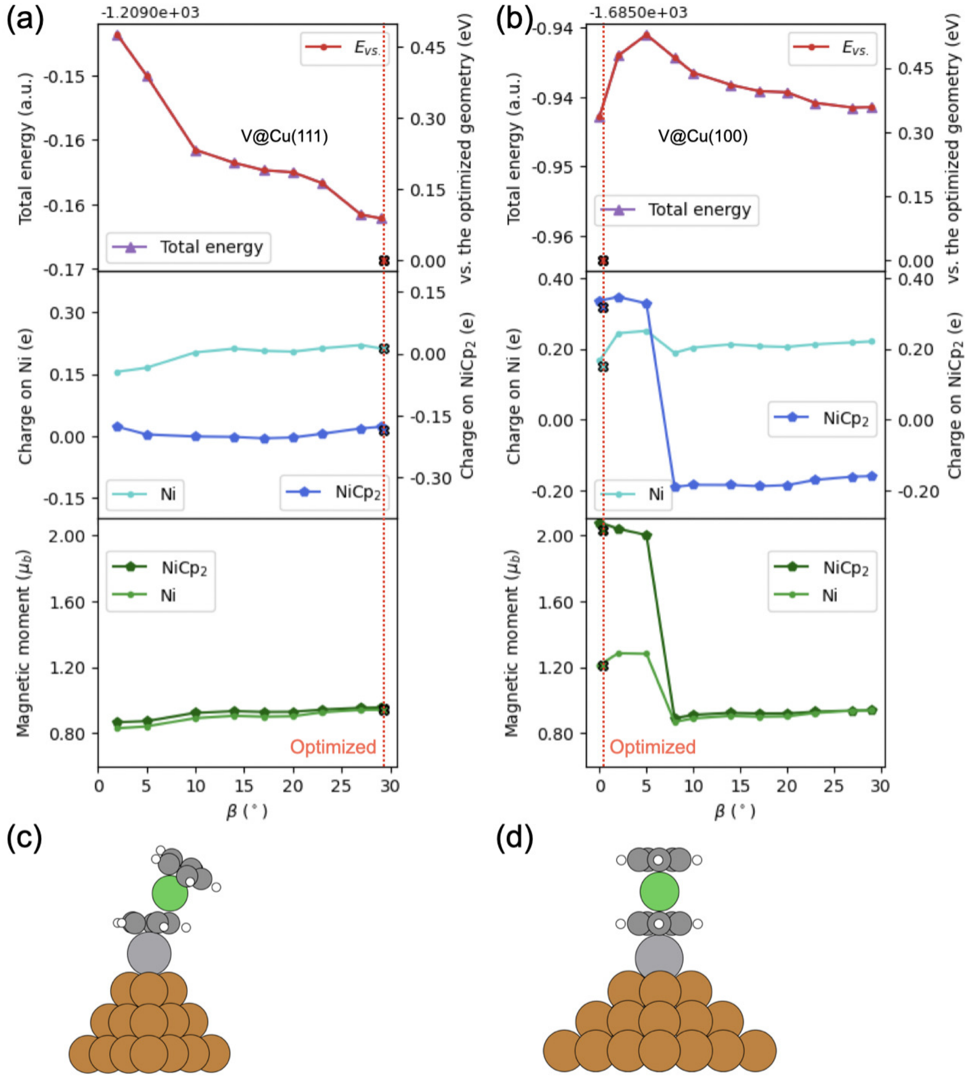

Figure S55: Energy, charge, and spin moment of $NiCp_2$ vs. β for (a) V on Cu(111), and (b) V on Cu(100). The corresponding optimized geometries are shown below panel (c) and (d). Properties of the optimized geometries are indicated by crosses with black edges.

S.5 The tilted behavior for $NiCp_2$@Cu(100)-terminated tip at different DFT-based functionals

To explain the observed tilted behavior of $NiCp_2$@Cu(100) terminated STM tip, we simulated two experimental processes involving an Fe@Cu(100) substrate and a $NiCp_2$@Cu(100)-terminated tip. In these simulations, the distance between the outermost layers of the two parts was manually adjusted, while allowing the intermediate atoms to freely optimize their positions (Figure S56). The exchange energy (E_{ex}) was calculated by $E_p - E_{ap}$, where P (AP) refers to the ferromagnetic (anti-ferromagnetic) exchange coupling between $NiCp_2$ and Fe. This energy serves as a metric for the exchange-coupling interaction between the two components.

In the first process (Figure S56(a)), the two parts started at a large separation, and the distance was gradually decreased until $NiCp_2$ became compressed. In the second process (Figure S56(b)), the distance was incrementally increased, starting from the compressed configuration, until the exchange coupling between the Fe adatom and Ni approached zero.

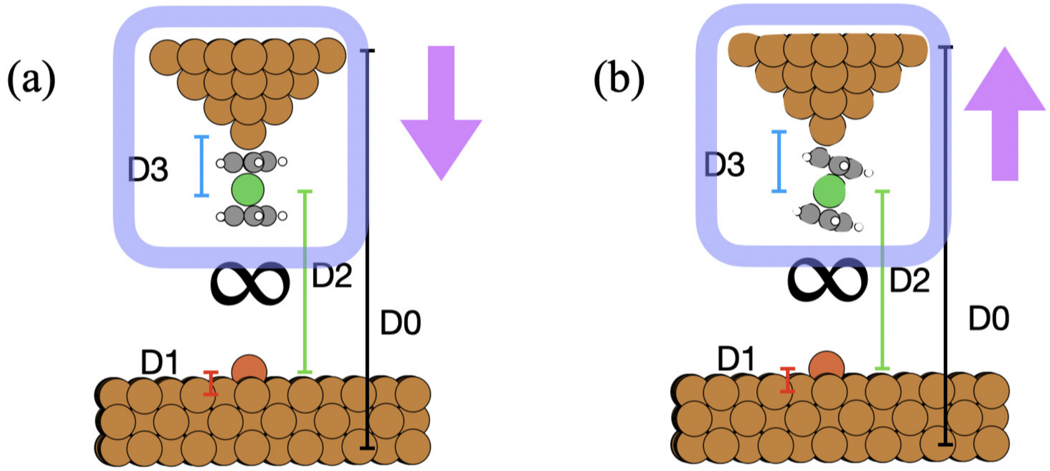

Figure S56: Motion diagrams illustrating the $NiCp_2$@Cu(100) tip interacting with an Fe adatom on a Cu(100) substrate. (a) The tip approaches the Fe adatom, representing the initial stage. (b) The tip retracts from the substrate, showing the final stage where the tip and substrate are completely separated.

During the first process, as shown in Figure S57, a "mutation point" is observed at the boundary between the blue and green lines, where the geometry of $NiCp_2$ begins to tilt. This is accompanied by a sudden reduction in the molecular spin of $NiCp_2$ to a half from $S = 1$. This point corresponds to the transition between the contact and tunnel regions (in STM), as also observed in M. Ormaza's work, which reported similar sharp transformations in the geometry and magnetic properties of $NiCp_2$ [8]. Once the tip comes into close contact with the substrate, the $NiCp_2$ molecule reverts to an upright configuration.

In the second process, starting from the compressed structure, the distance between the $NiCp_2$@Cu@Cu(100) tip and the Fe@Cu(100) substrate is progressively increased. The $NiCp_2$ remains tilted throughout this process, and the total energy increases gradually. Once the two components are sufficiently far apart, the spin of $NiCp_2$ returns to $S = 1$,and the two Cp rings become parallel again but retain a tilt angle of approximately 15°. The tilted configuration of $NiCp_2$ on the Cu tip can be considered a metastable state, with a total energy 0.02 eV higher than the upright configuration (as calculated at the PBE-D3 level of theory).

The tilted $NiCp_2$@Cu substrates is consistent with simulated models based on Cu(100) orientation with tilted $NiCp_2$ reported by M. Mohr, M. Ormaza, and B. Verlhac et al. [8–10]. In their simulations, the PBE-D2 functional was used with the projected augmented wave (PAW) method in VASP, which differs from this work.

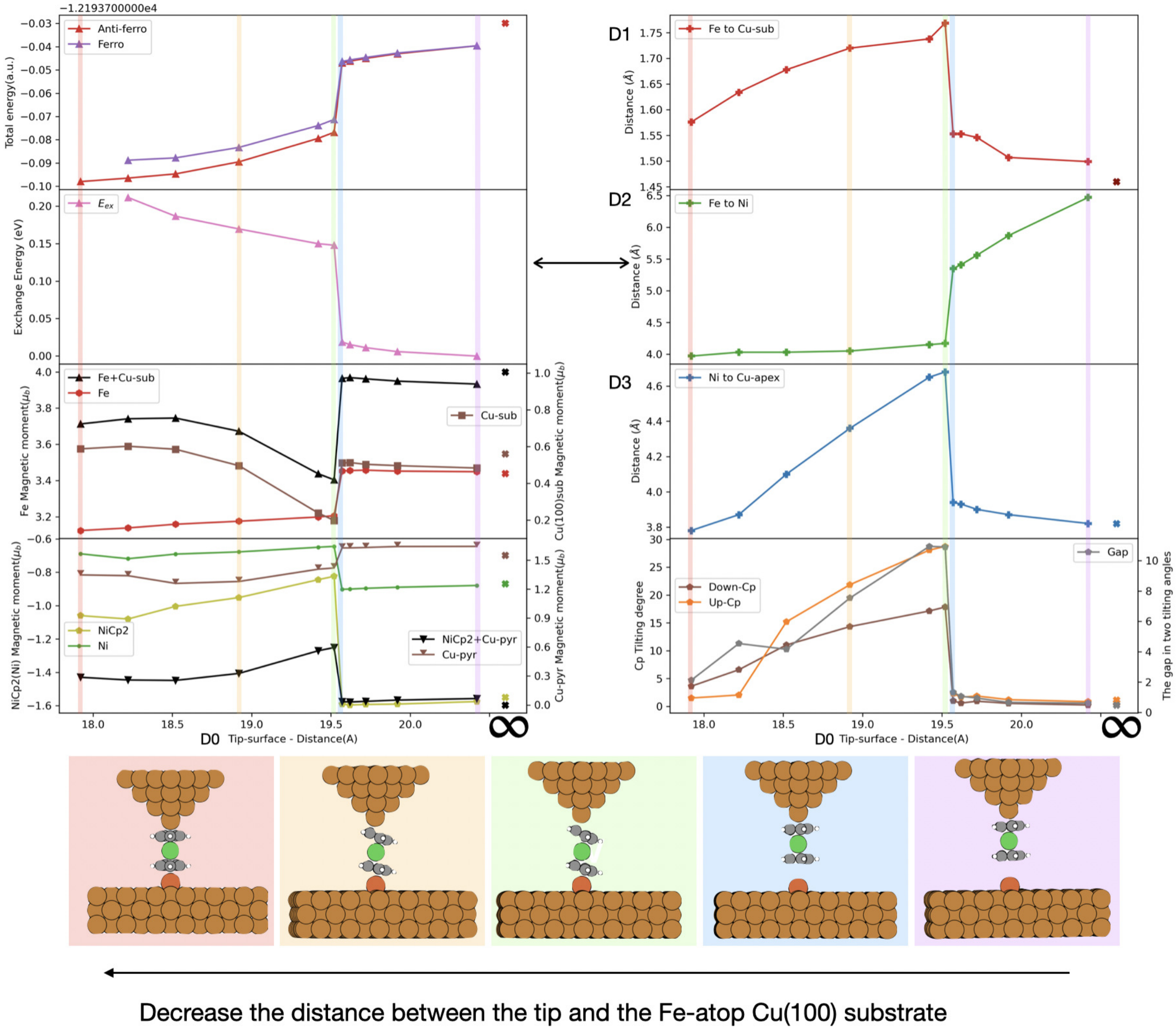

Figure S57: The plots for the total energy, the exchange energy, spin moment on Fe and $NiCp_2$, and the distances between atoms and substrates or atoms, changing with the decreasing distance between two outside layers (D0), during the first process.

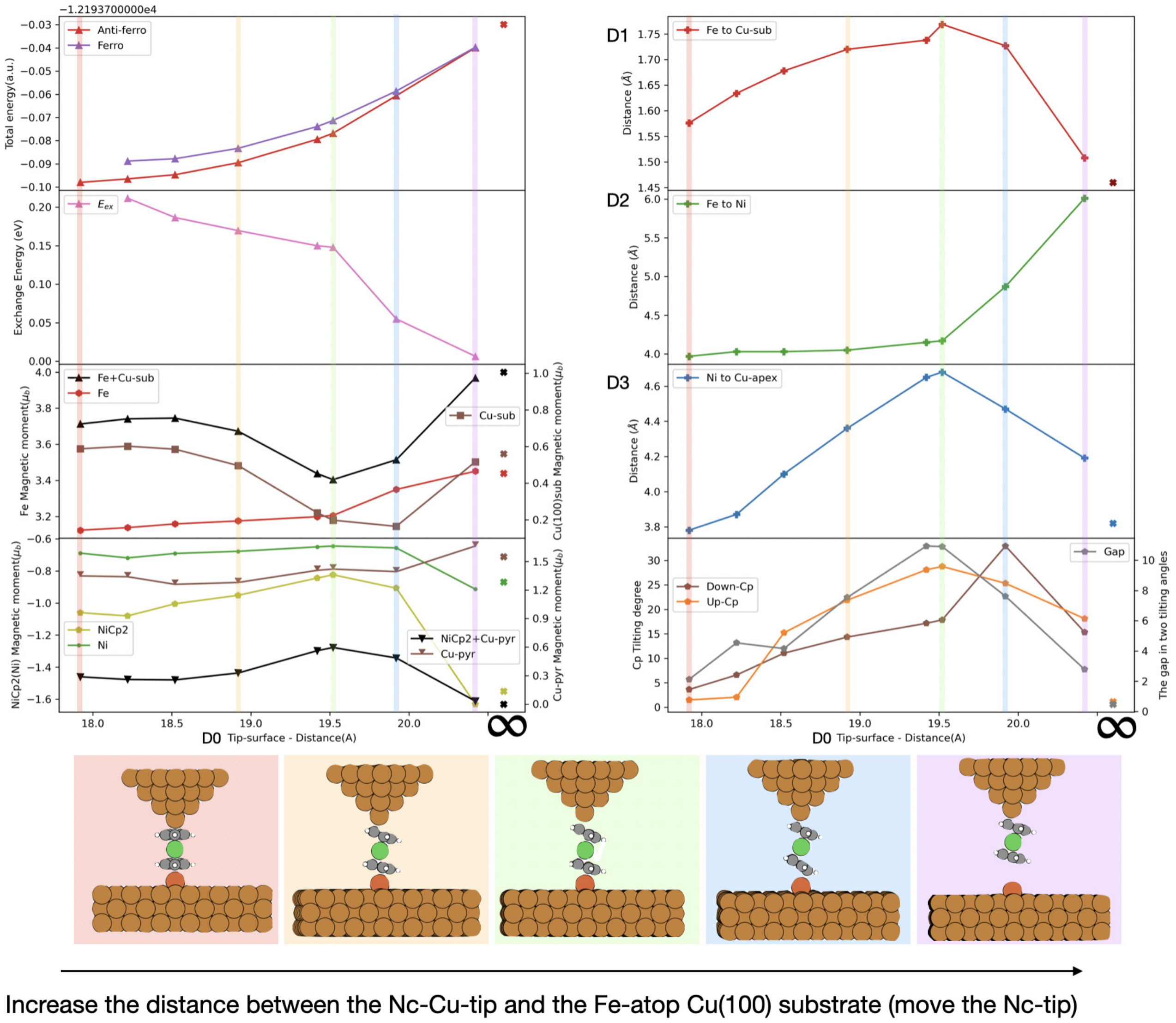

Figure S58: Plots for the total energy, the exchange energy, spin moment on Fe and $NiCp_2$, and the distances between atoms and substrates or atoms, changing with the increasing distance between two outside layers (D0), during the second process.

S.5.1 Comparison between the tilted and non-tilted $NiCp_2$@Cu(100) tips at different levels of theory

Table S10: Comparison of the total energy (a.u.) for tilted and non-tilted $NiCp_2$ configurations on a Cu(100)-based pyramid structure. Geometries were optimized at the PBE-D3, PBE-D2, and PBE0-D3 levels of theory, followed by single-point calculations. Red text highlights the lower energy value, and ΔE represents the energy difference, calculated as $E_t - E_n$.

Functionals	Tilted $NiCp_2$	Non-tilted $NiCp_2$	$\Delta E(eV)$
Optimized at PBE-D3, CP2K	(a.u.)	(a.u.)	
PBE-D3	-1675.05550076	-1675.05635411	0.023
D3 (Dispersion Correction)	-0.22437100	-0.22670509	0.064
Optimized at PBE-D2, CP2K			
PBE-D2	-1674.98254604	-1674.98279901	0.007
D2(Dispersion Correction)	-0.15149627	-0.15289555	0.038
Optimized at PBE0-D3, CP2K			
PBE0-D3	-1662.15949208	-1662.16038408	0.024
D3(Dispersion Correction)	-0.22620109	-0.22950401	0.090

According to Table S10, the energy difference between the tilted and non-tilted structures, optimized using the same functional with either D2 or D3 dispersion correction, consistently shows a slight preference for the non-tilted geometry. The stability difference is less than 0.03 eV in all cases. Both D2 and D3 dispersion corrections favor the non-tilted configuration of $NiCp_2$.

Table S11 presents the geometric information for the optimized structures. For the tilted configurations, the tilting angles range between 12 ° and 16°, with a discrepancy of less than 3° between the two Cp rings. This indicates that the Cp rings remain nearly parallel, despite the overall tilt of the molecule. Using D2 dispersion, the tilted $NiCp_2$@Cu-terminated tip tends to adopt slightly larger tilting angles compared to the geometries optimized with PBE-D3 and PBE0-D3.

Table S11: Structural details of the tilted and non-tilted $NiCp_2$@Cu-terminated tips optimized at PBE-D3, PBE-D2, and PBE0-D3 levels of theory.

	Tilted			Non-tilted		
	PBE-D3	PBE-D2	PBE0-D3	PBE-D3	PBE-D2	PBE0-D3
d_{Ni-Cu}(Å)	4.10	4.11	4.08	3.82	3.81	3.79
d_{Cu-sub}(Å)	1.68	1.67	1.68	1.65	1.66	1.63
$\alpha(°)$	15.87	16.37	13.43	1.12	1.64	0.12
$\beta(°)$	1.92	2.50	1.70	0.49	0.98	0.05

S.5.2 Computational details

The Fe@Cu(100) sample was modeled as a three-layer Cu(100) slab with lateral dimensions of 21.73 × 21.73 Å^2, corresponding to a 6 × 6 unit cell replicas. A single Fe atom was positioned at the hollow site of the Cu(100) surface to serve as the magnetic center. The $NiCp_2$@Cu(100) terminated tip was placed directly above the Fe atom, with Fe, Ni, and the Cu apex atom aligned along a vertical axis. To simulate the compression and stretching processes, the distance between the tip and the sample was systematically adjusted, and the geometry of the entire system was optimized at the PBE-D3 level of theory. During optimization, the two bottom layers of the Cu slab were kept fixed to mimic substrate constraints. Single-point energy calculations were also performed at the PBE-D3 level, with the electronic structure and magnetic properties analyzed using Mulliken population analysis.

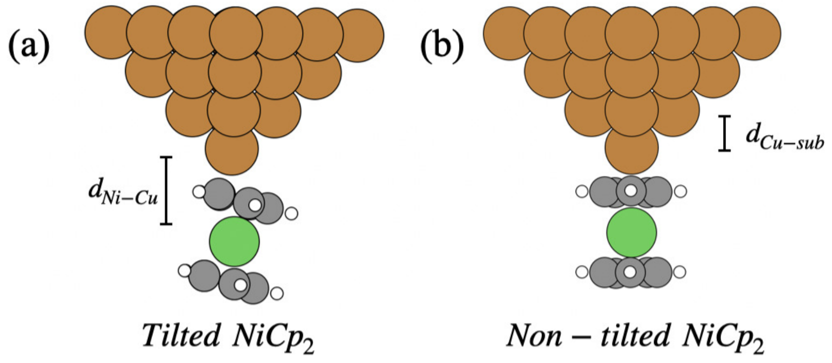

Figure S59: Diagrams for the (a) tilted and (b) non-tilted $NiCp_2$@Cu-terminated tips. The details for their distances and angles are listed in Table S11

For assessing the relative stability of non-tilted and tilted $NiCp_2$-terminated tips, the tip structure was relaxed separately with both the substrate and tip bottom layers fixed. These calculations were carried out at various levels of theory, including PBE-D3, PBE-D2, and PBE0-D3. Energy convergence for single-point calculations was achieved to an accuracy of 1×10^{-7} Hartree.

References

[1] Y. J. Franzke, F. Bruder, S. Gillhuber, C. Holzer, F. Weigend, *J. Phys. Chem. A* **2024**, *128*, 3 670.

[2] J. Vaara, S. A. Rouf, J. Mareš, *J. Chem. Theory Comput.* **2015**, *11*, 10 4840.

[3] L. Hedberg, K. Hedberg, *J. Chem. Phys.* **1970**, *53*, 3 1228.

[4] S. Li, Y. M. Hamrick, R. J. Van Zee, W. Weltner, *J. Am. Chem. Soc.* **1992**, *114*, 11 4433.

[5] R. Prins, J. D. W. van Voorst, C. J. Schinkel, *Chem. Phys. Lett.* **1967**, *1*, 2 54.

[6] P. Baltzer, A. Furrer, J. Hulliger, A. Stebler, *Inorg. Chem.* **1988**, *27*, 9 1543.

[7] F. Neese, *J. Chem. Phys.* **2007**, *127*, 16 164112.

[8] M. Ormaza, P. Abufager, B. Verlhac, N. Bachellier, M.-L. Bocquet, N. Lorente, L. Limot, *Nat. Commun.* **2017**, *8*, 1 1974.

[9] M. Mohr, M. Gruber, A. Weismann, D. Jacob, P. Abufager, N. Lorente, R. Berndt, *Phys. Rev. B* **2020**, *101*, 7 075414.

[10] B. Verlhac, N. Bachellier, L. Garnier, M. Ormaza, P. Abufager, R. Robles, M.-L. Bocquet, M. Ternes, N. Lorente, L. Limot, *Science* **2019**, *366*, 6465 623.
